# Microwave-Assisted Synthesis of Imidazole-Based Chalcones: Modulating Antimicrobial Activity Through Alkoxy Substitutions

**DOI:** 10.3390/antibiotics15030310

**Published:** 2026-03-18

**Authors:** Elnar Mammadov, Nilüfer Bayrak, Neslihan Beyazit, Emel Mataraci-Kara, Amaç Fatih TuYuN

**Affiliations:** 1 Department of Chemistry, Faculty of Science, Istanbul University, Fatih, 34134 Istanbul, Türkiye; 2 Institute of Graduate Studies in Science, Istanbul University, Fatih, 34134 Istanbul, Türkiye; 3 Department of Chemistry, Faculty of Arts and Sciences, Hatay Mustafa Kemal University, 31040 Hatay, Türkiye; 4 Department of Pharmaceutical Microbiology, Pharmacy Faculty, Istanbul University, Beyazit, 34116 Istanbul, Türkiye

**Keywords:** antibacterial activity, chalcones, biofilm formation, bactericidal effect, imidazole, heterocycles

## Abstract

**Background/Objectives**: The emergence of antimicrobial resistance necessitates the development of new and effective antimicrobial agents. In this study, three different series of imidazole-based chalcones (**IBC1-25**) were designed and synthesised using a sustainable approach, with the aim of identifying compounds with enhanced antimicrobial activity. **Methods**: A series of monoalkoxy, dialkoxy, and trialkoxy imidazole-based chalcones (**IBC1–25**) were synthesised and evaluated for their antimicrobial and antifungal activities against a range of microbial strains. Structure-activity relationships were analysed, and molecular docking studies were performed to investigate potential binding interactions with biofilm-associated regulatory proteins. In addition, ADME properties were predicted to assess drug-likeness. **Results**: Among the monoalkoxy derivatives (**IBC1-14**), **IBC5** exhibited the broadest spectrum of activity, particularly against *S. epidermidis*. Several dialkoxy analogues (**IBC17-21**) demonstrated improved potency, with **IBC20** showing notably high activity. While **IBC22** and **IBC25** were largely ineffective, **IBC23** and **IBC24** displayed significant antibacterial and antifungal activities. Overall, dialkoxy and trialkoxy derivatives exhibited enhanced efficacy, whereas monoalkoxy compounds with bulky or long-chain substituents were generally less active. The presence of multiple alkoxy substituents, such as methoxy and ethoxy groups, on the phenyl ring significantly improved activity, particularly against fungi and Gram-positive bacteria. Molecular docking studies revealed that **IBC20** and **IBC23** showed favourable binding to the biofilm-associated regulator TcaR, suggesting a potential allosteric inhibition mechanism, while weak interactions were observed with TagF. ADME predictions indicated good oral absorption and compliance with key drug-likeness criteria. **Conclusions**: The results demonstrate that both the number and type of alkoxy substituents play a critical role in antimicrobial activity. In particular, **IBC20** and **IBC23** emerge as promising candidates for further development as antimicrobial agents targeting biofilm-associated pathways.

## 1. Introduction

The discovery of new molecules that have therapeutic impacts on the body is one of the main objectives of modern medicinal chemistry [1]. Scientists have found that understanding how various lead structures may combat microorganisms and overcome resistance is essential to creating the next generation of antibiotics [2]. Natural products have played a crucial role throughout the history of antibiotic discovery and the creation of novel antimicrobial drugs, providing valuable leads and promising candidates to tackle resistance and develop novel antibiotics with enhanced antibacterial potency due to their structural diversity and unique bioactivities. They have characteristics like natural selection, typically low levels of toxicity, great cellular specificity, and extensive physicochemical variety since they have evolved over millions of years, unlike wholly synthesized molecules [3,4,5]. Notwithstanding their pharmacological importance, natural product-derived lead compounds are often associated with fundamental disadvantages such as limited antibacterial effectiveness, weak chemical stability, and insufficient solubility [6]. As a consequence, reasonable structural alteration has become a crucial strategy to get around these disadvantages while strengthening their therapeutic potential. The effectiveness of this approach in modern drug discovery is demonstrated by the large number of semi-synthetic derivatives of natural compounds that have been successfully introduced into clinical practice to date [7,8].

The discovery that microwaves, initially used only as a heating source in the kitchen, could also be used as a thermal source in a chemistry laboratory revolutionised synthetic chemistry methodology. From the earliest days when a primitive microwave oven used in the kitchen was tested to carry out chemical reactions, to the advanced modern devices used today to activate and accelerate organic reactions using microwave radiation, tens of thousands of studies have been conducted [9,10]. These studies have revealed that microwave-assisted organic synthesis offers numerous advantages over classical methods. Because microwaves can reach much higher temperatures in much shorter periods than conventional heating, chemical reactions that sometimes take days can be completed in minutes. Furthermore, this technique provides uniform and selective heating with lower energy consumption. Higher purity and yield of products formed in reactions, and higher reproducibility of reactions are among its most important advantages. Due to its remarkable advantages, such as simplicity, cleanliness, speed, efficiency, and economy, microwave-assisted organic synthesis has begun to replace conventional methods in the synthesis of many molecules [11].

**Figure 1:**
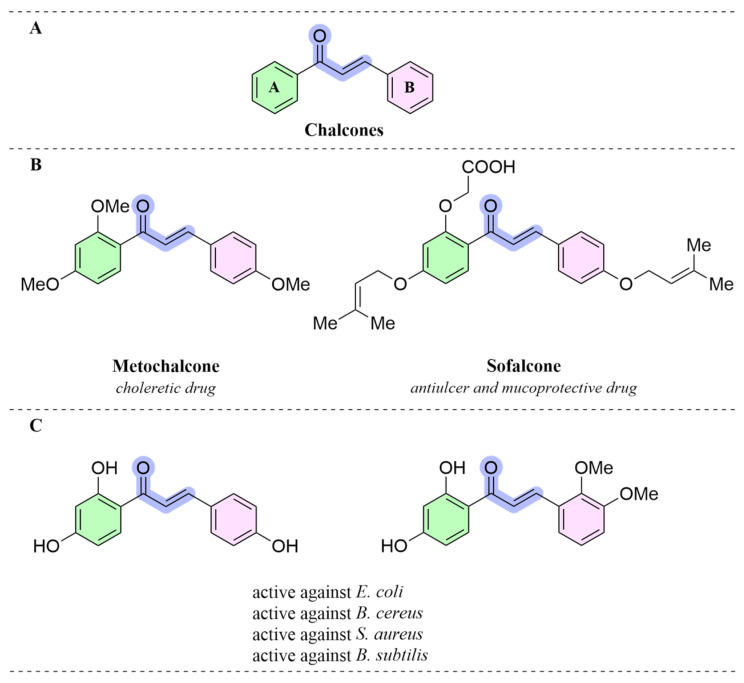
The structures of important chalcone molecules.

Chalcone structures (*E*-1,3-diphenyl-2-propen-1-one) shown in Figure 1A, which belong to the flavonoids family, are a class of open-chain bioactive natural products composed of two aromatic ring systems connected by an α,β-unsaturated carbonyl moiety [12,13] and are well known for their diverse biological activities, including anticancer, anti-inflammatory, antioxidant, antibacterial, and antiviral activities [14,15,16,17]. Some lead compounds derived from chalcones, including metochalcone and sofalcone, have been approved for clinical applications as a choleretic agent and as an antiulcer, mucoprotective agent (Figure 1B), respectively [18,19]. According to previous studies, chalcone derivatives are very suitable pivotal structures for the discovery of novel antibacterial lead structures. Some chalcones were isolated through an activity-guided purification process from *Astragalus adsurgens* plants infected with the pathogenic fungus *Embellisia astragali*. These compounds exhibited remarkable antibacterial activity with a minimum inhibitory concentration (MIC) as low as 7.8 mg/mL (Figure 1C) [20].

**Figure 2:**
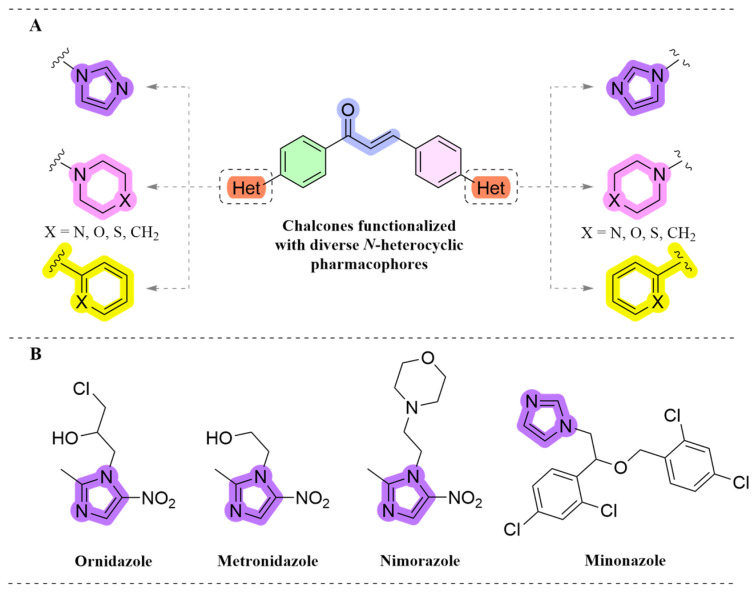
(**A**) Chalcones functionalized with diverse *N*-heterocyclic pharmacophores; (**B**) Representative marketed imidazole-based drugs.

Heterocyclic structures are common in biologically active small molecules and natural products, and they are essential structural components of various biomolecules like DNA and RNA as well as many different kinds of pharmaceutical substances [21]. Notably, the importance of heterocyclic moieties in medicinal chemistry is shown by the fact that it is estimated that more than 90% of clinically approved drugs contain them. Due to their wide range of structural versatility and biological capabilities, *N*-heterocyclic structures are becoming especially well-known [22]. They are pharmacophoric parts that can act at receptor sites as both hydrogen bond donors and acceptors, influencing important aspects including pharmacokinetics, pharmacodynamics, ionization constants (pKa), and total bioavailability. Numerous molecular classes, such as amino acids, natural alkaloids, antifungal azoles, and quinolone-based antimicrobials, serve as examples of these characteristics [23]. Chalcones functionalized with diverse *N*-heterocyclic pharmacophores, including piperazines, piperidines, morpholines, and imidazoles, have been rationally designed, as illustrated in Figure 2A, and subsequently found to exhibit promising biological potency against a variety of microorganisms and diseases, so they might be potential candidates for the development of novel agents [24,25,26,27,28,29,30,31,32]. Imidazoles are important nitrogen heterocyclic molecules with a five-membered ring that contains two nitrogen atoms, which enables them to form intermolecular bonds such as hydrogen bonds with either protein receptors or enzymes in the body [33,34]. Imidazole-based drugs, including ornidazole, metronidazole, nimorazole, miconazole, and butoconazole shown in Figure 2B, have been employed effectively as antimicrobial agents and are clinically **utilised** in the treatment of various infectious diseases [35]. Given the potential therapeutic significance of such molecules, we chose to further explore this class of compounds. Based on data from the literature, our aim in this study is to effectively utilize the chalcone pharmacophore as the lead scaffold and insert an imidazole ring and other active group(s) to synthesize a series of novel imidazole-based chalcones (IBCs*)* and to evaluate their antimicrobial activities as well as perform further in-depth biological tests.

## 2. Results and Discussion

### 2.1. Design Strategy and Chemistry

**Figure 3:**
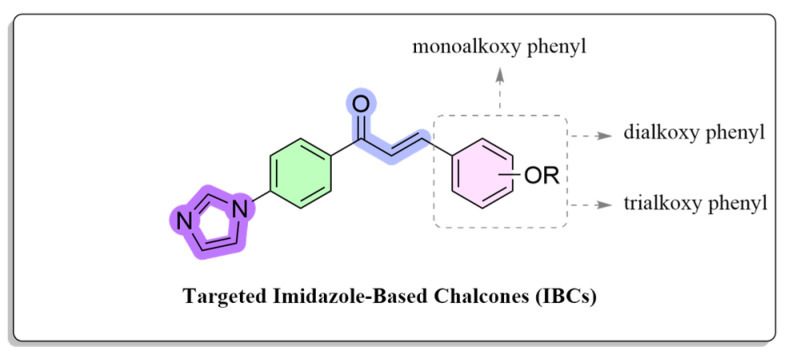
The design strategy of the targeted imidazole-based chalcones (IBCs).

**Scheme 1:**
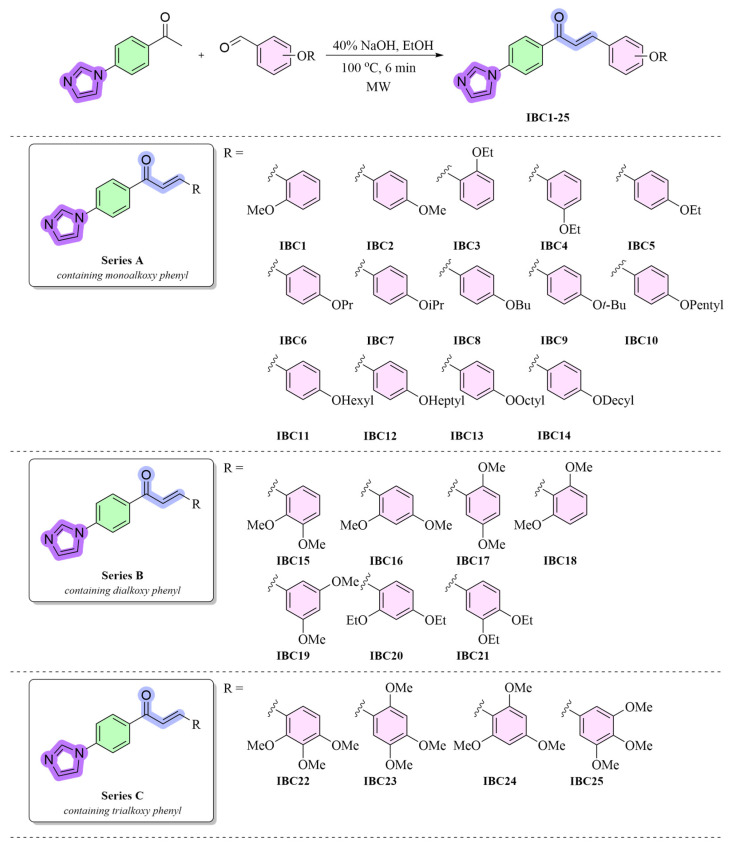
Synthesis and substrate scope of IBCs (**IBC1-25**) from imidazole-based acetophenone.

Based on previous investigations, the alkoxy aryl hydrophobic moiety has been identified to constitute an essential pharmacophoric component for biological effect. To assess the influence of electronic enrichment on these scaffolds along with the incorporated imidazole moiety, this moiety was on occasion substituted with additional bioisosteres, such as monoalkoxy, dialkoxy, and trimethoxyphenyl groups (Figure 3). Many researchers have synthesized the chalcones using various methods because of their intriguing biological activities [36]. A sustainable and eco-friendly method was demonstrated by the successful use of microwave irradiation to synthesize imidazole-based chalcones (IBCs) by the reaction of corresponding substituted aldehydes with electron-releasing group(s) (monoalkoxy, dialkoxy, and trialkoxy substituted aldehydes) with imidazole-based acetophenone in the presence of sodium hydroxide as a base at 100 °C were synthesized according to the literature method [24]. Microwave-assisted synthesis can reduce reaction times from hours to minutes and provides rapid and uniform heating, which dramatically accelerates reaction rates. The concise synthetic route of IBCs is shown in Scheme 1. Through modifying the number and position of substituents, the chalcone formation was carried out employing a Claisen-Schmidt condensation that was particularly designed for the synthesis of IBCs. This allowed for an in-depth investigation of structure-activity relationships (SARs). All spectral results of the IBCs agreed with the suggested structures, and the structures of the chalcones were characterized through ^1^H NMR, ^13^C NMR, FTIR, and HRMS spectral data.

### 2.2. Antibacterial and Antifungal Activity Evaluation

#### 2.2.1. Determination of Minimum Inhibitory Concentrations (MIC)

The obtained IBCs (**IBC1-25**) were initially tested for their antibacterial and antifungal activities against four strains of Gram-negative bacteria, three strains of Gram-positive bacteria, and three fungi by determining their minimum inhibitory concentration (MIC) values. The MIC values employed for the screening against bacteria and fungi, with the results of the antimicrobial activity presented in Table 1. To investigate the significance of the IBCs (**IBC1-25**), their antimicrobial activities were evaluated in comparison with those of commercially available broad-spectrum reference agents. Overall activity is quite low in the monoalkoxy analogues (**IBC1-14**). Only a small number of molecules demonstrated some kind of antifungal effect, while most of them had no impact. For instance, two IBCs (**IBC3** and **IBC4**) had a MIC of 312.50 µg/mL and were only effective against *C. albicans*. **IBC5** was more noteworthy since it showed efficacy against *C. albicans* with MIC value 156.25 µg/mL, *S. aureus* with MIC value 19.53 µg/mL, *S. epidermidis* with MIC value 39.06 µg/mL, *E. coli* with MIC value 312.500 µg/mL, *P. aeruginosa* with MIC value 625 µg/mL, and *C. parapsilosis* with MIC value 78.12 µg/mL. Furthermore, **IBC10** showed only weak activity against *C. parapsilosis* (625 µg/mL) and *C. albicans* (312.50 µg/mL). The spectrum of activity is more extensive for the dialkoxy derivatives (**IBC15-21**). Whereas the MIC values showed that **IBC15-17** and **IBC19** did not have any antibacterial activity (one exception for both **IBC15** and **IBC19**), **IBC18** and **IBC21** showed low potency against both Gram-negative and Gram-positive strains with MIC values ranging from 39.06 to 625 μg/mL. Two analogues (**IBC20-21**) also demonstrated broad-spectrum action against fungi with MIC values ranging from 39.06 to 156.25 μg/mL. Among them, **IBC20** was particularly the most potent, displaying efficacy against *S. epidermidis* with an MIC of 9.76 µg/mL equal to that of the control drug of Cefuroxime. Among the trialkoxy analogues (**IBC22-25**), **IBC23** and **IBC24** were more potent, showing activity against both bacteria and fungi. Most of the organisms were all resistant to **IBC22** and **IBC25**.

While dialkoxy and trialkoxy analogues often show more prevalent and stronger activity, almost all of the monosubstituted analogues with a single alkoxy group are inactive. However, the presence of two to three alkoxy groups on the same phenyl ring typically increases action, especially against fungi and Gram-positive bacteria. This is supported by the fact that most monosubstituted analogues exhibit no action, whereas a number of IBCs in the dialkoxy and trialkoxy analogues exhibit measurable efficacy against diverse microorganisms. Long-chain alkoxys (such as hexyl, heptyl, octyl, and decyl) are inactive in the monoalkoxy analogues. Likewise, bulky substituents or branched alkoxys (*i*-Pr, *t*-Bu) typically decrease activity or have erratic effects. This implies that branching creates steric hindrance and that extremely long chains have a detrimental effect on solubility and/or target accessibility. Therefore, small to medium-sized alkoxy groups, i.e., methoxy and ethoxy groups, or several small groups exhibit the best activity.

**Figure 4:**
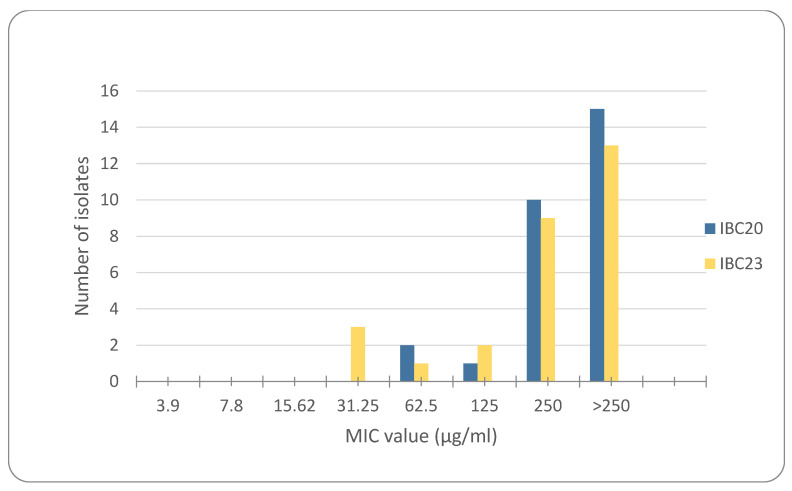
The in vitro activities of **IBC20** and **IBC23** against clinically derived multidrug-resistant *Staphylococcus* spp.

According to the findings in Table 1, the MIC values of **IBC20** and **IBC23**, which showed efficacy against *Staphylococcus* spp., were further examined against 28 multidrug-resistant *Staphylococcus* spp. strains obtained from various clinical samples. The results are shown in Figure 4. The higher MICs detected in MDR isolates compared to the MIC values of **IBC20** and **IBC23** in standard *Staphylococcus* strains suggest that this resistance is due to the multidrug resistance mechanisms possessed by these isolates. Therefore, it would be beneficial for future studies to investigate the resistance mechanism responsible for these increased MIC concentrations in MDR isolates for the tested molecules.

#### 2.2.2. Determination of Time-Kill Curve Kinetic Study

**Figure 5:**
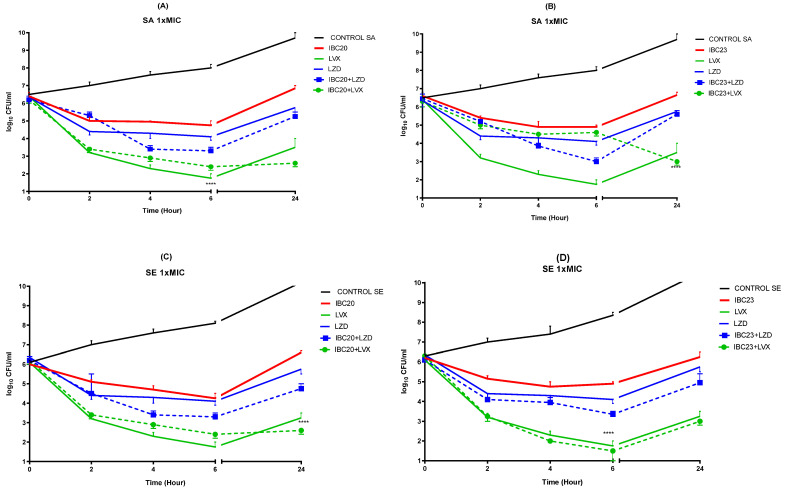
The analysis of the time-kill curve. (**A**) **IBC20** alone or in combination with levofloxacin or linezolid for *S. aureus* ATCC 29213; (**B**) **IBC23** alone or in combination with levofloxacin or linezolid for *S. aureus* ATCC 29213; (**C**) **IBC20** alone or in combination with levofloxacin or linezolid for *S. epidermidis* ATCC 12228; (**D**) **IBC23** alone or in combination with levofloxacin or linezolid for *S. epidermidis* ATCC 12228 at concentrations of 1 × MIC. The horizontal axis illustrates the duration of the killing process, whereas the vertical axis shows the logarithmic survival rates of the bacteria that were tested. (**** *p* < 0.0001) (SA: *S. aureus*, SE: *S. epidermidis*, LVX: levofloxacin, LZD: linezolid).

Time-kill Curve (TKC) studies were conducted on the *S. aureus* ATCC 29213 and *S. epidermidis* ATCC 12228 strains, as shown in Figure 5. The TKC demonstrated that neither the tested molecules (**IBC20** and **IBC23**) nor antibiotics utilised bactericidal action against the investigated strain at 1 × MIC within 24 h (Figure 5). However, we observed additive effects when we combined the **IBC20** or **IBC23** with levofloxacin or linezolid at 1 × MIC. Moreover, no antagonism was detected in any combination.

The growing prevalence of antibiotic resistance in *Staphylococcus* spp. presents an important threat to worldwide public health. Because of genetic mutations or the acquisition of exogenous drug-resistant genes in bacteria, the efficiency of a single antibiotic is often inadequate; thus, combination therapy has emerged as an essential therapeutic method for drug-resistant *Staphylococcus* spp. Combination treatment often entails administering two or more antibiotics concurrently to maximize antibacterial activity via synergy. Furthermore, the risk that a pathogen would develop resistance to the combination of medications is substantially lower than that associated with a monotherapy strategy. This therapy aims to eradicate bacteria to the fullest, hence reducing the likelihood of bacterial adaptability and the development of resistance [37,38,39,40,41]. Therefore, our molecules combined with commonly used antibiotics may create an additive effect that ensures the long-term use of these existing antibiotics for treating resistant infections.

#### 2.2.3. Evaluation of the In Vitro Antibiofilm Activity

*Staphylococcus* species, particularly *Staphylococcus aureus* and *Staphylococcus epidermidis*, are well-known for their ability to adhere to abiotic surfaces and form robust biofilms. These sessile communities are embedded in a self-produced extracellular polymeric substance matrix, primarily composed of polysaccharides, proteins, and extracellular DNA. Biofilm formation significantly enhances the antimicrobial resistance of Staphylococcal cells, contributing to the persistence of infections, especially in nosocomial environments involving indwelling medical devices such as catheters and prosthetic implants. It is estimated that biofilm-associated infections account for approximately 60% of recurrent microbial diseases in humans. Within biofilms, Staphylococcal cells can exhibit resistance levels up to 10–1000 times greater than their planktonic counterparts, rendering conventional antibiotic therapies largely ineffective. This resistance complicates clinical management and contributes to the global burden of chronic and device-related infections. Consequently, novel therapeutic strategies and antibiofilm agents are being actively investigated to overcome the limitations of traditional antimicrobial approaches and improve treatment outcomes [42,43,44].

**Figure 6:**
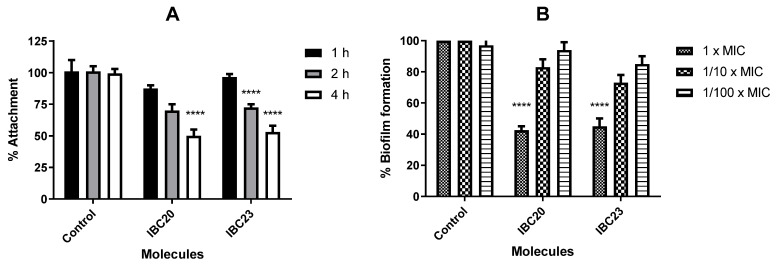
Inhibition of MRSA. (**A**) Wells with 1/10 × MIC of molecules and 1 × 10^6^ CFU/200 μL inoculum incubated for 1, 2, or 4 h at 37 °C; (**B**) Wells with 1×, 1/10×, or 1/100 × MIC and 5 × 10^6^ CFU/200 μL inoculum incubated for 24 h at 37 °C. Microorganisms without molecules are 100% approved in control bars. Molecules were examined in six wells. Each experiment represents three independent tests. All biofilm differences between control and molecule-treated were statistically significant. (**** *p* < 0.0001).

In the present study, the examined compounds (**IBC20** and **IBC23**) were evaluated at a sub-MIC (1/10 × MIC), demonstrating comparable efficacy in biofilm adhesion to the wells (Figure 6A). All examined compounds exhibited optimal activity within 4 h relative to earlier time points. The cell attachment percentage to the wells was around 50% relative to control after 4 h. Upon analyzing the biofilm formation percentage of the examined strain (Figure 6B), the inhibition rates were influenced by the tested doses, exhibiting significant fluctuations; the highest inhibition rates were recorded at 1 × MICs for the evaluated compounds, as anticipated.

#### 2.2.4. In Silico Molecular Docking Studies

Based on the in vitro antimicrobial activity results, **IBC20** and **IBC23**, which exhibited the strongest activity against *S. epidermidis*, molecular docking studies were conducted to explore possible protein targets that may be associated with their biological effects. These computational analyses were intended to provide structural insight into potential molecular interactions underlying the experimental findings. Two proteins functionally implicated in biofilm formation were selected as docking targets: TcaR (PDB ID: 3KP4), a transcriptional regulator of the icaADBC operon, and TagF (PDB ID: 3L7L), a wall teichoic acid (WTA) polymerase involved in cell wall biosynthesis. The docking protocol was validated by re-docking the co-crystallized ligands into their respective binding sites, affording RMSD values of 0.7585 Å for TcaR (Figure S1) and 1.2613 Å for TagF (Figure S2), confirming the suitability of the computational setup for reproducing crystallographic binding orientations.

Biofilm development in *S. epidermidis* depends both on structural components and transcriptional regulation. TagF participates in WTA chain elongation, contributing to cell wall stability and biofilm architecture [45]. TcaR, a member of the MarR family, regulates the ica operon responsible for polysaccharide intercellular adhesin (PIA) production, a key constituent of the biofilm matrix [46,47]. Given the established role of these proteins in biofilm-associated processes, they were selected as biologically relevant targets for in silico investigation.

**Figure 7:**
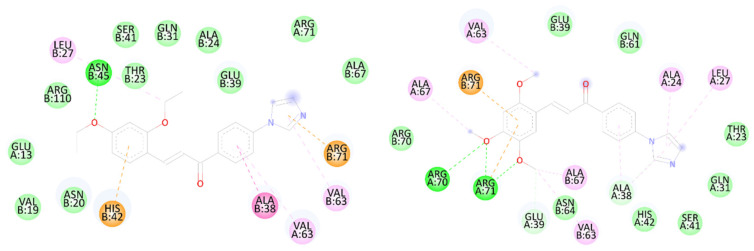
A 2D-interaction plot of **IBC20** and **IBC23** with TcaR (PDB: 3KP4). Interaction types: Hydrogen bonds are shown as green dashed lines, van der Waals interactions in light green, π–alkyl interactions as orange dashed lines, π–π (or amide–π) stacking in purple, and π–cation interactions as red dashed lines.

The calculated binding energies are summarized in Table 2. Both **IBC20** and **IBC23** demonstrated favourable predicted binding affinities toward TcaR and TagF, with **IBC20** exhibiting the most favourable docking score against TcaR (−8.1 kcal/mol), comparable to that of the reference compound. Examination of the predicted binding poses suggests that both compounds can be accommodated within the reported allosteric pocket of TcaR located near helices α1–α2. In the docking models, polar contacts involving Asn20 and hydrophobic interactions with residues such as Leu27 and His42 were observed (Figure 7), consistent with previously described ligand-TcaR interaction patterns [43].

**IBC23** formed a higher number of hydrogen-bond interactions within the predicted binding site, whereas **IBC20** displayed stronger hydrophobic complementarity. Notably, **IBC20** also exhibited slightly superior antibacterial potency in vitro, which may be consistent with its favourable predicted binding energy.

In contrast, docking to TagF revealed less favourable interaction characteristics. Although both compounds could be positioned within the enzyme cavity, they did not reproduce the highly anionic interaction network typically required for CDP-glycerol recognition (Figure S3). This suggests comparatively weaker structural complementarity with TagF relative to TcaR within the constraints of the docking model.

Taken together, the docking results provide a structurally plausible explanation that is compatible with the experimentally observed antibiofilm activity of **IBC20** and **IBC23**, particularly in relation to TcaR. Nevertheless, molecular docking represents a predictive computational approach and does not constitute direct evidence of target engagement or enzymatic inhibition. Further mechanistic investigations, such as biochemical binding assays or gene expression analyses, would be required to conclusively establish the molecular target responsible for the observed biological effects.

#### 2.2.5. In Silico Drug-Likeness and ADME Analysis

To obtain a preliminary understanding of the pharmacokinetic behaviour of the most active compounds (**IBC20** and **IBC23**), an in silico drug-likeness and ADME evaluation was performed using the SwissADME platform (Table 3). These analyses were intended to provide supportive insight into physicochemical suitability rather than definitive evidence of clinical applicability.

Both compounds possess molecular weights within the acceptable range for orally administered small molecules (**IBC20**: 362 g/mol; **IBC23**: 364 g/mol). Their topological polar surface area (TPSA) values (53.35 Å^2^ and 62.58 Å^2^, respectively), combined with the absence of hydrogen bond donors and moderate hydrogen bond acceptor counts, suggest favourable passive membrane permeability. In addition, both molecules comply with widely accepted drug-likeness filters, including Lipinski, Ghose, Veber, Egan, and Muegge criteria, without violations.

The predicted high gastrointestinal absorption and bioavailability score (0.55) further indicate reasonable oral drug-likeness potential. Their calculated lipophilicity values (XlogP3: 4.21 for **IBC20** and 3.35 for **IBC23**) fall within the commonly accepted range for drug-like molecules, while the predicted aqueous solubility values suggest moderate solubility [45]. Neither compound is predicted to be a P-glycoprotein substrate, which may favour intracellular retention. However, both compounds are predicted to inhibit several CYP450 isoforms, indicating a potential for metabolic interactions that would require experimental clarification.

It is important to emphasize that the present computational assessment was limited to physicochemical and pharmacokinetic descriptors. No in silico toxicity predictions were included in this study. Therefore, the current ADME evaluation should be interpreted as an initial developability screen rather than a comprehensive safety assessment. Detailed in vitro and in silico toxicity investigations will be necessary in future studies to establish the safety profile of these compounds and to further evaluate their therapeutic potential.

## 3. Conclusions

In this work, three different series of chalcones (IBCs, **IBC1-25**), including monoalkoxylated phenyl chalcones and multialkoxylated phenyl chalcones ring B, were designed and synthesized via the reactions between imidazole-based acetophenone and corresponding substituted benzaldehydes by using a sustainable and eco-friendly method. IBC analogues have been characterized by ^1^H and ^13^C NMR, FTIR, and HRMS. By integrating the imidazole heterocyclic moiety into the chalcone privileged fragment in one scaffold, these novel IBCs were developed as potential antibacterial agents to explore new candidates for combating bacterial infections. All compounds were evaluated for their antibacterial and antifungal activities, and their detailed SARs were obtained. Only a small number of monoalkoxy analogues (**IBC1-14**) showed weak antifungal effects, primarily against *C. albicans*, and generally showed low antibacterial efficacy. Dialkoxy (**IBC15-21**) and trialkoxy (**IBC22-25**) analogues, on the other hand, showed stronger and wider antibacterial activity; **IBC20** was the most potent, comparable to the control drug cefuroxime. While dialkoxy and trialkoxy analogues with small to medium-sized alkoxy groups show improved antibacterial activity, especially against fungi and Gram-positive bacteria, monoalkoxy analogues are essentially inactive. Long-chain or bulky substituents decrease efficacy, most likely as a result of steric hindrance, reduced solubility, or decreased accessibility to the target. Based on these results, given the significant activity of the found IBCs against Gram-positive bacteria, we were interested in two IBC analogues (**IBC20** and **IBC23**) to further examine their antimicrobial potential, with a particular emphasis on antibiofilm activity. In addition, a time–kill curve study was conducted to test bactericidal activity. Multidrug-resistant *Staphylococcus* spp. infections are one of the most common causes of infection in hospitals. It has also been associated with increased incidence of disease, mortality, duration of stay, and treatment expenditures [48]. In our study, their compatibility with levofloxacin and linezolid, two drugs often used in clinics to treat resistant staphylococcal infections, underscores the importance of these compounds’ antibacterial characteristics. Although the Food and Drug Administration has approved a large number of drugs for MRSA since 2014, the fact that the fatality rate associated with invasive MRSA infections remains continuously high highlights the need to do more research in this field for those who are afflicted [49,50]. We urgently need to develop more efficient anti-MRSA treatments and implement infection management techniques to meet the current demand. Further research in this area could potentially mitigate the clinical implications of this condition. The relevance of these molecules in future antibacterial applications is highlighted by the fact that they are compatible with current antibiotics, which is helpful in the treatment of severe illnesses. This is especially true in cases of infections that are resistant to medication and need combination therapy. The overall results from the antimicrobial, molecular docking, and ADME tests show that **IBC20** and **IBC23** mostly work by changing TcaR instead of stopping TagF. Their strong TcaR binding, good pharmacokinetic profiles, and compliance with all drug-likeness rules show that they could be developed further as anti-biofilm agents. Subsequent in vivo and mechanistic investigations will be crucial to confirm their therapeutic relevance. Accordingly, **IBC20** and **IBC23** emerge as lead structures from this study and represent suitable candidates for advanced in vitro validation and future lead optimization efforts.

## 4. Experimental

### 4.1. Chemicals and Apparatus

Melting points (mp) were recorded with an electrical melting point (Büchi B-540, Flawil, Switzerland) and are uncorrected. All chemicals used within this study were bought from various commercial sources with a minimum purity of 95%. All reagents were used without further any purification. Reactions were performed using an Anton Paar Monowave 400 Microwave Synthesis Reactor (Graz, Austria) in sealed G30 reaction vials with magnetic stirring. Reaction temperatures and times are specified in the experimental procedure. The reactions were checked by thin-layer chromatography (TLC). TLC was carried out using aluminum-based DC-plates (Silica gel 60 F_254_) and TLC plates were visualized using UV light (254 nm). Column chromatography was conducted under medium pressure on a Silica gel 60 (63–200 µm particle-sized) purchased from Merck (Darmstadt, Germany) with an appropriate solvent system as eluents. High-resolution mass spectra electrospray ionization (HRMS-ESI) analyses was performed using a Waters SYNAPT G1 MS (Milford, MA, USA). The infrared (IR) spectra of all analogues were obtained on a FTIR spectrometer, using the single reflection diamond ATR module. Nuclear magnetic resonance (NMR) were carried out using a Bruker Avance III™ HD 600 MHz NMR spectrometer (Billerica, MA, USA, 600 MHz frequency for proton NMR, 125 MHz frequency for carbon NMR) in the specified deuterated solvent, respectively. Chemical shifts were reported in parts per million (ppm) in CDCl_3_ and coupling constants (J) were given in hertz (Hz).

### 4.2. General Procedure for the Synthesis of the Imidazole-Based Chalcones (IBC1-25)

To a solution of 4′-(imidazol-1-yl)acetophenone (1.00 mmol, 0.1862 g) and the corresponding substituted benzaldehydes (1.00 mmol) in ethanol (5 mL) was placed in a reaction vial G30 (Anton Paar, Graz, Austria) sealed with a silicone septum and a snap cap, with magnetic stirring at 600 rpm. To this solution, ethanolic solution of 40% NaOH (0.274 mL) was added at room temperature. The vial was placed in an Anton Paar Monowave 400 Microwave Synthesis Reactor (Graz, Austria) and heated to the desired reaction temperature (90 °C) within 1 min, then maintained at 100 °C for 6 min under microwave irradiation. After the completion of the reaction, the temperature was decreased to 55 °C in the reactor. Subsequently, the reaction mixture was further cooled to room temperature (if necessary, the vial was placed in a fridge), and the precipitate was collected by filtration, washed with cold ethanol, and dried. Finally, the crude product was purified by silica gel column chromatography using an appropriate solvent as the eluent to give corresponding IBC analogues, if required.

#### 4.2.1. 1-(4-(1*H*-Imidazol-1-yl)phenyl)-3-(2-methoxyphenyl)prop-2-en-1-one (IBC1) [35]

Following the general procedure by applying 2-methoxybenzaldehyde (0.1362 g), the crude residue was purified by column chromatography to furnish IBC1 as a yellow solid. Yield: 55%, mp 115.4–115.7 °C. FTIR (ATR) υ (cm^−1^): 3102 (CH_aromatic_), 2920, 2850 (CH_aliphatic_), 1661 (>C=O), 1591, 1524, 1483, 1462, 1425, 1373, 1339, 1294, 1250, 1215, 1190, 1157, 1123, 1103, 1049, 1013. ^1^H NMR (600 MHz, *CDCl*_3_) *δ* (ppm): δ 8.19–8.12 (m, 3H, CH_aromatic_), 7.97 (s, 1H, CH_aromatic_), 7.66–7.62 (m, 2H, CH_aromatic_ and CH_vinyl_), 7.53 (d, *J* = 8.6 Hz, 2H, CH_aromatic_), 7.43–7.38 (m, 1H, CH_aromatic_), 7.38–7.36 (m, 1H, CH_aromatic_), 7.26 (s, 1H, CH_aromatic_), 7.01 (t, *J* = 7.5 Hz, 1H, CH_aromatic_), 6.98–6.94 (m, 1H, CH_vinyl_), 3.93 (s, 3H, OCH_3_). ^13^C NMR (150 MHz, *CDCl*_3_) *δ* (ppm): 189.49 (>C=O), 158.94, 141.16, 140.32, 137.26, 135.43, 132.07, 131.10, 130.51, 129.45, 123.71, 122.27, 120.82, 120.77, 117.79, 111.31 (C_aromatic_ and C_vinyl_), 55.59 (OCH_3_). HRMS(+ESI) *m*/*z* calcd for C_19_H_17_N_2_O_2_ [M + H]^+^: 305.1290; found: 305.1290.

#### 4.2.2. 1-(4-(1*H*-Imidazol-1-yl)phenyl)-3-(4-methoxyphenyl)prop-2-en-1-one (IBC2) [35]

Following the general procedure by applying 4-methoxybenzaldehyde (0.1362 g), the crude residue was purified by column chromatography to furnish **IBC2** as a yellow solid. Yield: 64%, mp 141.6–141.9 °C. FTIR (ATR) υ (cm^−1^): 3105, 3067 (CH_aromatic_), 2995, 2845 (CH_aliphatic_), 1659 (>C=O), 1597, 1578, 1510, 1487, 1422, 1375, 1344, 1317, 1258, 1225, 1173, 1063, 1017. ^1^H NMR (600 MHz, *DMSO-d*_6_) *δ* (ppm): δ 8.43 (s, 1H, CH_aromatic_), 8.26 (d, *J* = 8.6 Hz, 2H, CH_aromatic_), 7.89 (s, 1H, CH_aromatic_), 7.85–7.82 (m, 5H, CH_aromatic_ and C_vinyl_), 7.72 (d, *J* = 15.5 Hz, 1H, C_vinyl_), 7.14 (s, 1H, CH_aromatic_), 6.99 (d, *J* = 8.7 Hz, 2H, CH_aromatic_), 3.79 (s, 3H, OCH_3_). ^13^C NMR (150 MHz, *DMSO-d*_6_) *δ* (ppm): 188.08 (>C=O), 161.87, 144.64, 140.51, 136.21, 136.18, 131.34, 130.81, 130.79, 127.73, 120.20, 119.72, 118.23, 114.84 (C_aromatic_ and C_vinyl_), 55.81 (OCH_3_). HRMS(+ESI) *m*/*z* calcd for C_19_H_17_N_2_O_2_ [M + H]^+^: 305.1290; found: 305.1290.

#### 4.2.3. 1-(4-(1*H*-Imidazol-1-yl)phenyl)-3-(2-ethoxyphenyl)prop-2-en-1-one (IBC3)

Following the general procedure by applying 2-ethoxybenzaldehyde (0.1502 g), the crude residue was purified by column chromatography to furnish **IBC3** as a yellow solid. Yield: 65%, mp 92.6–93.8 °C. FTIR (ATR) υ (cm^−1^): 3129, 3105 (CH_aromatic_), 2918, 2849 (CH_aliphatic_), 1657 (>C=O), 1591, 1526, 1513, 1487, 1454, 1429, 1375, 1296, 1250, 1221, 1194, 1173, 1123, 1101, 1057, 1030, 1015. ^1^H NMR (600 MHz, *CDCl*_3_) *δ* (ppm): δ 8.12–8.03 (m, 3H, CH_aromatic_ and C_vinyl_), 7.90 (s, 1H, CH_aromatic_), 7.65 (d, *J* = 15.8 Hz, 1H, C_vinyl_), 7.55 (dd, *J* = 7.7, 1.3 Hz, 1H, CH_aromatic_), 7.45 (d, *J* = 8.6 Hz, 2H, CH_aromatic_), 7.34–7.25 (m, 2H, CH_aromatic_), 7.19 (d, *J* = 5.3 Hz, 1H, CH_aromatic_), 6.92 (t, *J* = 7.5 Hz, 1H, CH_aromatic_), 6.87 (d, *J* = 8.3 Hz, 1H, CH_aromatic_), 4.08 (q, *J* = 7.0 Hz, 2H, OCH_2_), 1.46 (t, *J* = 7.0 Hz, 3H, OCH_2_CH_3_). ^13^C NMR (150 MHz, *CDCl*_3_) *δ* (ppm): 188.40 (>C=O), 157.43, 140.49, 139.29, 136.30, 134.44, 130.97, 130.06, 129.42, 128.99, 122.65, 121.20, 119.75, 119.67, 116.79, 111.13 (C_aromatic_ and C_vinyl_), 63.01 (OCH_2_), 13.87 (OCH_2_CH_3_). HRMS(+ESI) *m*/*z* calcd for C_20_H_19_N_2_O_2_ [M + H]^+^: 319.1447; found: 319.1430.

#### 4.2.4. 1-(4-(1*H*-Imidazol-1-yl)phenyl)-3-(3-ethoxyphenyl)prop-2-en-1-one (IBC4)

Following the general procedure by applying 3-ethoxybenzaldehyde (0.1502 g), the crude residue was purified by column chromatography to furnish **IBC4** as a yellow solid. Yield: 57%, mp 102.3–103.2 °C. FTIR (ATR) υ (cm^−1^): 3121 (CH_aromatic_), 2978, 2928 (CH_aliphatic_), 1657 (>C=O), 1593, 1578, 1518, 1485, 1391, 1302, 1292, 1179, 1165, 1113, 1030. ^1^H NMR (600 MHz, *CDCl*_3_) *δ* (ppm): δ 8.16 (d, *J* = 8.5 Hz, 2H, CH_aromatic_), 7.99 (s, 1H, CH_aromatic_), 7.81 (d, *J* = 15.6 Hz, 1H, C_vinyl_), 7.54 (d, *J* = 8.5 Hz, 2H, CH_aromatic_), 7.51 (d, *J* = 15.6 Hz, 1H, C_vinyl_), 7.38 (s, 1H, CH_aromatic_), 7.34 (t, *J* = 7.9 Hz, 1H, CH_aromatic_), 7.28–7.22 (m, 2H, CH_aromatic_), 7.18 (s, 1H, CH_aromatic_), 6.98 (dd, *J* = 8.2, 2.2 Hz, 1H, CH_aromatic_), 4.09 (q, *J* = 7.0 Hz, 2H, OCH_2_), 1.45 (t, *J* = 7.0 Hz, 3H, OCH_2_CH_3_). ^13^C NMR (150 MHz, *CDCl*_3_) *δ* (ppm): 188.84 (>C=O), 159.39, 145.56, 140.49, 136.93, 136.00, 135.44, 131.06, 130.52, 130.03, 121.61, 121.13, 120.84, 117.79, 117.00, 114.24 (C_aromatic_ and C_vinyl_), 63.64 (OCH_2_), 14.81 (OCH_2_CH_3_). HRMS(+ESI) *m*/*z* calcd for C_20_H_19_N_2_O_2_ [M + H]^+^: 319.1447; found: 319.1442.

#### 4.2.5. 1-(4-(1*H*-Imidazol-1-yl)phenyl)-3-(4-ethoxyphenyl)prop-2-en-1-one (IBC5)

Following the general procedure by applying 4-ethoxybenzaldehyde (0.1502 g), the crude residue was purified by column chromatography to furnish **IBC5** as a yellow solid. Yield: 54%, mp 160.7–162.4 °C. FTIR (ATR) υ (cm^−1^): 3119 (CH_aromatic_), 2980, 2940 (CH_aliphatic_), 1653 (>C=O), 1605, 1589, 1568, 1524, 1510, 1483, 1464, 1422, 1364, 1304, 1250, 1225, 1185, 1160, 1113, 1088, 1042. ^1^H NMR (600 MHz, *CDCl*_3_) *δ* (ppm): δ 8.15 (d, *J* = 8.4 Hz, 2H, CH_aromatic_), 7.97 (s, 1H, CH_aromatic_), 7.83 (d, *J* = 15.5 Hz, 1H, C_vinyl_), 7.61 (d, *J* = 8.5 Hz, 2H, CH_aromatic_), 7.53 (d, *J* = 8.4 Hz, 2H, CH_aromatic_), 7.41 (d, *J* = 15.6 Hz, 1H, C_vinyl_), 7.38 (s, 1H, CH_aromatic_), 7.26 (s, 1H, CH_aromatic_), 6.94 (d, *J* = 8.5 Hz, 2H, CH_aromatic_), 4.09 (q, *J* = 7.0 Hz, 2H, OCH_2_), 1.45 (t, *J* = 7.0 Hz, 3H, OCH_2_CH_3_). ^13^C NMR (150 MHz, *CDCl*_3_) *δ* (ppm): 188.84 (>C=O), 161.38, 145.49, 140.32, 137.28, 135.43, 131.09, 130.41, 130.39, 127.21, 120.79, 118.91, 117.79, 114.98 (C_aromatic_ and C_vinyl_), 63.71 (OCH_2_), 14.72 (OCH_2_CH_3_). HRMS(+ESI) *m*/*z* calcd for C_20_H_19_N_2_O_2_ [M + H]^+^: 319.1447; found: 319.1448.

#### 4.2.6. 1-(4-(1*H*-imidazol-1-yl)phenyl)-3-(4-propoxyphenyl)prop-2-en-1-one (IBC6)

Following the general procedure by applying 4-propoxybenzaldehyde (0.1642 g), the crude residue was purified by column chromatography to furnish **IBC6** as a yellow solid. Yield: 60%, mp 110–112 °C. FTIR (ATR) υ (cm^−1^): 3125 (CH_aromatic_), 2967, 2930, 2876 (CH_aliphatic_), 1651 (>C=O), 1597, 1585, 1568, 1510, 1474, 1423, 1395, 1339, 1292, 1260, 1220, 1180, 1103, 1034, 1011. ^1^H NMR (600 MHz, *CDCl*_3_) *δ* (ppm): δ 8.14 (d, *J* = 8.2 Hz, 2H, CH_aromatic_), 7.98 (s, 1H, CH_aromatic_), 7.82 (d, *J* = 15.5 Hz, 1H, C_vinyl_), 7.60 (d, *J* = 8.4 Hz, 2H, CH_aromatic_), 7.52 (d, *J* = 8.2 Hz, 2H, CH_aromatic_), 7.41 (d, *J* = 15.6 Hz, 1H, C_vinyl_), 7.37 (s, 1H, CH_aromatic_), 7.25 (s, 1H, CH_aromatic_), 6.94 (d, *J* = 8.4 Hz, 2H, CH_aromatic_), 3.97 (t, *J* = 6.5 Hz, 2H, OCH_2_), 1.89–1.76 (m, 2H, OCH_2_CH_2_), 1.05 (t, *J* = 7.4 Hz, 3H, CH_3_). ^13^C NMR (150 MHz, *CDCl*_3_) *δ* (ppm): 188.81 (>C=O), 161.58, 145.51, 140.26, 137.26, 135.42, 130.97, 130.41, 130.38, 127.14, 120.76, 118.82, 117.82, 115.00 (C_aromatic_ and C_vinyl_), 69.70 (OCH_2_), 22.48, 10.49 (CH_2_CH_3_). HRMS(+ESI) *m*/*z* calcd for C_21_H_21_N_2_O_2_ [M + H]^+^: 333.1603; found: 333.1604.

#### 4.2.7. 1-(4-(1*H*-imidazol-1-yl)phenyl)-3-(4-isopropoxyphenyl)prop-2-en-1-one (IBC7)

Following the general procedure by applying 4-isopropoxybenzaldehyde (0.1642 g), the crude residue was purified by column chromatography to furnish **IBC7** as a yellow solid. Yield: 47%, mp 131–133 °C. FTIR (ATR) υ (cm^−1^): 3063 (CH_aromatic_), 2978, 2938 (CH_aliphatic_), 1647 (>C=O), 1601, 1585, 1557, 1508, 1483, 1429, 1375, 1306, 1294, 1254, 1215, 1173, 1103, 1051, 1032. ^1^H NMR (600 MHz, *CDCl*_3_) *δ* (ppm): δ 8.14 (d, *J* = 8.4 Hz, 2H, CH_aromatic_), 7.98 (s, 1H, CH_aromatic_), 7.82 (d, *J* = 15.6 Hz, 1H, C_vinyl_), 7.60 (d, *J* = 8.6 Hz, 2H, CH_aromatic_), 7.52 (d, *J* = 8.4 Hz, 2H, CH_aromatic_), 7.41 (d, *J* = 15.6 Hz, 1H, C_vinyl_), 7.37 (s, 1H, CH_aromatic_), 7.25 (s, 1H, CH_aromatic_), 6.92 (d, *J* = 8.6 Hz, 2H, CH_aromatic_), 4.66–4.59 (m, 1H, OCH), 1.38 (s, 3H, CH_3_), 1.37 (s, 3H, CH_3_). ^13^C NMR (150 MHz, *CDCl*_3_) *δ* (ppm): 188.83 (>C=O), 160.43, 145.51, 140.26, 137.30, 135.42, 130.98, 130.45, 130.38, 126.99, 120.78, 118.80, 117.81, 116.01 (C_aromatic_ and C_vinyl_), 70.10 (OCH), 21.96 (CH_3_). HRMS(+ESI) *m*/*z* calcd for C_21_H_21_N_2_O_2_ [M + H]^+^: 333.1603; found: 333.1603.

#### 4.2.8. 1-(4-(1*H*-imidazol-1-yl)phenyl)-3-(4-butoxyphenyl)prop-2-en-1-one (IBC8)

Following the general procedure by applying 4-butoxybenzaldehyde (0.1782 g), the crude residue was purified by column chromatography to furnish **IBC8** as a yellow solid. Yield: 56%, mp 118–121 °C. FTIR (ATR) υ (cm^−1^): 3092 (CH_aromatic_), 2955, 2930, 2870 (CH_aliphatic_), 1651 (>C=O), 1605, 1587, 1566, 1522, 1476, 1425, 1371, 1341, 1310, 1294, 1252, 1215, 1182, 1117, 1105, 1057, 1028. ^1^H NMR (600 MHz, *CDCl*_3_) *δ* (ppm): δ 8.14 (d, *J* = 8.4 Hz, 2H, CH_aromatic_), 7.98 (s, 1H, CH_aromatic_), 7.82 (d, *J* = 15.5 Hz, 1H, C_vinyl_), 7.61 (d, *J* = 8.6 Hz, 2H, CH_aromatic_), 7.52 (d, *J* = 8.4 Hz, 2H, CH_aromatic_), 7.41 (d, *J* = 15.5 Hz, 1H, C_vinyl_), 7.37 (s, 1H, CH_aromatic_), 7.25 (s, 1H, CH_aromatic_), 6.94 (d, *J* = 8.5 Hz, 2H, CH_aromatic_), 4.01 (t, *J* = 6.5 Hz, 2H, OCH_2_), 1.83–1.76 (m, 2H, OCH_2_CH_2_), 1.55–1.46 (m, 2H, CH_2_CH_3_), 0.99 (t, *J* = 7.4 Hz, 3H, CH_3_). ^13^C NMR (150 MHz, *CDCl*_3_) *δ* (ppm): 188.82 (>C=O), 161.60, 145.52, 140.28, 137.28, 135.42, 131.01, 130.40, 130.38, 127.14, 120.78, 118.83, 117.80, 115.00 (C_aromatic_ and C_vinyl_), 67.93 (OCH_2_), 31.18, 19.21 (CH_2_CH_2_), 13.83 (CH_3_). HRMS(+ESI) *m*/*z* calcd for C_22_H_23_N_2_O_2_ [M + H]^+^: 347.1760; found: 347.1760.

#### 4.2.9. 1-(4-(1*H*-imidazol-1-yl)phenyl)-3-(4-(tert-butoxy)phenyl)prop-2-en-1-one (IBC9)

Following the general procedure by applying 4-*tert*-butoxybenzaldehyde (0.1782 g), the crude residue was purified by column chromatography to furnish **IBC9** as a yellow solid. Yield: 63%, mp 128–130 °C. FTIR (ATR) υ (cm^−1^): 3126 (CH_aromatic_), 2978, 2928 (CH_aliphatic_), 1655 (>C=O), 1595, 1560, 1503, 1481, 1420, 1395, 1366, 1290, 1250, 1219, 1180, 1161, 1103, 1036. ^1^H NMR (600 MHz, *CDCl*_3_) *δ* (ppm): δ 8.15 (d, *J* = 8.4 Hz, 2H, CH_aromatic_), 7.98 (s, 1H, CH_aromatic_), 7.83 (d, *J* = 15.6 Hz, 1H, C_vinyl_), 7.58 (d, *J* = 8.4 Hz, 2H, CH_aromatic_), 7.52 (d, *J* = 8.4 Hz, 2H, CH_aromatic_), 7.44 (d, *J* = 15.6 Hz, 1H, C_vinyl_), 7.38 (s, 1H, CH_aromatic_), 7.25 (s, 1H, CH_aromatic_), 7.04 (d, *J* = 8.4 Hz, 2H, CH_aromatic_), 1.41 (s, 9H, CH_3_). ^13^C NMR (150 MHz, *CDCl*_3_) *δ* (ppm): 188.79 (>C=O), 158.44, 145.26, 140.34, 137.12, 135.41, 131.02, 130.41, 129.63, 129.29, 123.64, 120.77, 119.84, 117.78 (C_aromatic_ and C_vinyl_), 79.51 (OC), 28.91 (CH_3_). HRMS(+ESI) *m*/*z* calcd for C_22_H_23_N_2_O_2_ [M + H]^+^: 347.1760; found: 347.1761.

#### 4.2.10. 1-(4-(1*H*-imidazol-1-yl)phenyl)-3-(4-(pentyloxy)phenyl)prop-2-en-1-one (IBC10)

Following the general procedure by applying 4-amyloxybenzaldehyde (0.1923), the crude residue was purified by column chromatography to furnish **IBC10** as a yellow solid. Yield: 58%, mp 99–100 °C. FTIR (ATR) υ (cm^−1^): 3138 (CH_aromatic_), 2941, 2870 (CH_aliphatic_), 1653 (>C=O), 1601, 1589, 1568, 1508, 1481, 1472, 1425, 1371, 1290, 1252, 1215, 1182, 1111, 1053. ^1^H NMR (600 MHz, *CDCl*_3_) *δ* (ppm): δ 8.14 (d, *J* = 8.0 Hz, 2H, CH_aromatic_), 7.98 (s, 1H, CH_aromatic_), 7.82 (d, *J* = 15.5 Hz, 1H, C_vinyl_), 7.60 (d, *J* = 8.2 Hz, 2H, CH_aromatic_), 7.51 (d, *J* = 8.1 Hz, 2H, CH_aromatic_), 7.41 (d, *J* = 15.6 Hz, 1H, C_vinyl_), 7.37 (s, 1H, CH_aromatic_), 7.25 (s, 1H, CH_aromatic_), 6.93 (d, *J* = 8.2 Hz, 2H, CH_aromatic_), 4.00 (t, *J* = 6.5 Hz, 2H, OCH_2_), 1.85–1.77 (m, 2H, OCH_2_CH_2_), 1.49–1.42 (m, 2H, CH_2_CH_2_CH_3_), 1.42–1.35 (m, 2H, CH_2_CH_3_), 0.94 (t, *J* = 7.1 Hz, 3H, CH_3_). ^13^C NMR (150 MHz, *CDCl*_3_) *δ* (ppm): 188.79 (>C=O), 161.59, 145.50, 140.24, 137.26, 135.41, 130.94, 130.41, 130.38, 127.12, 120.76, 118.80, 117.80, 114.99 (C_aromatic_ and C_vinyl_), 68.22 (OCH_2_), 28.83, 28.14, 22.43 (3xCH_2_), 14.02 (CH_3_). HRMS(+ESI) *m*/*z* calcd for C_23_H_25_N_2_O_2_ [M + H]^+^: 361.1916; found: 361.1916.

#### 4.2.11. 1-(4-(1*H*-imidazol-1-yl)phenyl)-3-(4-(hexyloxy)phenyl)prop-2-en-1-one (IBC11)

Following the general procedure by applying 4-hexyloxybenzaldehyde (0.2063), the crude residue was purified by column chromatography to furnish **IBC11** as a yellow solid. Yield: 60%, mp 114–115 °C. FTIR (ATR) υ (cm^−1^): 3125 (CH_aromatic_), 2947, 2924, 2866 (CH_aliphatic_), 1651 (>C=O), 1605, 1587, 1562, 1522, 1485, 1423, 1342, 1294, 1244, 1215, 1182, 1117, 1053. ^1^H NMR (600 MHz, *CDCl*_3_) *δ* (ppm): δ 8.14 (d, *J* = 8.4 Hz, 2H, CH_aromatic_), 7.97 (s, 1H, CH_aromatic_), 7.82 (d, *J* = 15.6 Hz, 1H, C_vinyl_), 7.60 (d, *J* = 8.6 Hz, 2H, CH_aromatic_), 7.52 (d, *J* = 8.4 Hz, 2H, CH_aromatic_), 7.41 (d, *J* = 15.5 Hz, 1H, C_vinyl_), 7.37 (s, 1H, CH_aromatic_), 7.25 (s, 1H, CH_aromatic_), 6.93 (d, *J* = 8.6 Hz, 2H, CH_aromatic_), 4.00 (t, *J* = 6.6 Hz, 2H, OCH_2_), 1.84–1.77 (m, 2H, OCH_2_CH_2_), 1.51–1.42 (m, 2H, OCH_2_CH_2_CH_2_), 1.39–1.30 (m, 4H, CH_2_CH_2_), 0.91 (t, *J* = 6.8 Hz, 3H, CH_3_). ^13^C NMR (150 MHz, *CDCl*_3_) *δ* (ppm): 188.82 (>C=O), 161.60, 145.53, 140.26, 137.28, 135.41, 130.97, 130.41, 130.39, 127.13, 120.77, 118.82, 117.80, 115.00 (C_aromatic_ and C_vinyl_), 68.25 (OCH_2_), 31.55, 29.11, 25.68, 22.59 (4xCH_2_), 14.03 (CH_3_). HRMS(+ESI) *m*/*z* calcd for C_24_H_27_N_2_O_2_ [M + H]^+^: 375.2073; found: 375.2072.

#### 4.2.12. 1-(4-(1*H*-imidazol-1-yl)phenyl)-3-(4-(heptyloxy)phenyl)prop-2-en-1-one (IBC12)

Following the general procedure by applying 4-heptyloxybenzaldehyde (0.2203), the crude residue was purified by column chromatography to furnish **IBC12** as a yellow solid. Yield: 58%, mp 84–85 °C. FTIR (ATR) υ (cm^−1^): 3117 (CH_aromatic_), 2920, 2851 (CH_aliphatic_), 1651 (>C=O), 1603, 1587, 1566, 1522, 1474, 1425, 1373, 1342, 1306, 1290, 1250, 1219, 1190, 1185, 1115, 1036. ^1^H NMR (600 MHz, *CDCl*_3_) *δ* (ppm): δ 8.13 (d, *J* = 8.4 Hz, 2H, CH_aromatic_), 7.97 (s, 1H, CH_aromatic_), 7.81 (d, *J* = 15.5 Hz, 1H, C_vinyl_), 7.59 (d, *J* = 8.5 Hz, 2H, CH_aromatic_), 7.51 (d, *J* = 8.4 Hz, 2H, CH_aromatic_), 7.40 (d, *J* = 15.5 Hz, 1H, C_vinyl_), 7.37 (s, 1H, CH_aromatic_), 7.24 (s, 1H, CH_aromatic_), 6.93 (d, *J* = 8.5 Hz, 2H, CH_aromatic_), 3.99 (t, *J* = 6.5 Hz, 2H, OCH_2_), 1.83–1.76 (m, 2H, OCH_2_CH_2_), 1.50–1.42 (m, 2H, OCH_2_CH_2_CH_2_), 1.40–1.24 (m, 6H, CH_2_CH_2_), 0.90 (t, *J* = 6.7 Hz, 3H, CH_3_). ^13^C NMR (150 MHz, *CDCl*_3_) *δ* (ppm): 188.72 (>C=O), 161.58, 145.45, 140.25, 137.20, 135.39, 131.01, 130.39, 130.36, 127.11, 120.70, 118.77, 117.76, 114.98 (C_aromatic_ and C_vinyl_), 68.23 (OCH_2_), 31.75, 29.14, 29.03, 25.95, 22.59 (5xCH_2_), 14.09 (CH_3_). HRMS(+ESI) *m*/*z* calcd for C_25_H_29_N_2_O_2_ [M + H]^+^: 389.2229; found: 389.2231.

#### 4.2.13. 1-(4-(1*H*-imidazol-1-yl)phenyl)-3-(4-(octyloxy)phenyl)prop-2-en-1-one (IBC13)

Following the general procedure by applying 4-octyloxybenzaldehyde (0.2343 g), the crude residue was purified by column chromatography to furnish **IBC13** as a yellow solid. Yield: 49%, mp 92–94 °C. FTIR (ATR) υ (cm^−1^): 3134 (CH_aromatic_), 2922, 2853 (CH_aliphatic_), 1653 (>C=O), 1589, 1568, 1508, 1474, 1423, 1371, 1288, 1254, 1217, 1173, 1128, 1103, 1028. ^1^H NMR (600 MHz, *CDCl*_3_) *δ* (ppm): δ 8.14 (d, *J* = 8.6 Hz, 2H, CH_aromatic_), 7.97 (s, 1H, CH_aromatic_), 7.82 (d, *J* = 15.5 Hz, 1H, C_vinyl_), 7.60 (d, *J* = 8.7 Hz, 2H, CH_aromatic_), 7.54–7.49 (m, 2H, CH_aromatic_), 7.40 (d, *J* = 15.6 Hz, 1H, C_vinyl_), 7.37 (s, 1H, CH_aromatic_), 7.25 (s, 1H, CH_aromatic_), 6.93 (d, *J* = 8.7 Hz, 2H, CH_aromatic_), 4.00 (t, *J* = 6.6 Hz, 2H, OCH_2_), 1.84–1.76 (m, 2H, OCH_2_CH_2_), 1.50–1.41 (m, 2H, OCH_2_CH_2_CH_2_), 1.39–1.24 (m, 8H, CH_2_CH_2_), 0.89 (t, *J* = 7.0 Hz, 3H, CH_3_). ^13^C NMR (150 MHz, *CDCl*_3_) *δ* (ppm): 188.76 (>C=O), 161.59, 145.49, 140.26, 137.23, 135.40, 131.01, 130.40, 130.37, 127.12, 120.73, 118.79, 117.78, 114.99 (C_aromatic_ and C_vinyl_), 68.24 (OCH_2_), 31.79, 29.33, 29.22, 29.14, 26.00, 22.65 (6xCH_2_), 14.10 (CH_3_). HRMS(+ESI) *m*/*z* calcd for C_26_H_31_N_2_O_2_ [M + H]^+^: 403.2386; found: 403.2386.

#### 4.2.14. 1-(4-(1*H*-imidazol-1-yl)phenyl)-3-(4-(decyloxy)phenyl)prop-2-en-1-one (IBC14)

Following the general procedure by applying 4-decyloxybenzaldehyde (0.2624 g), the crude residue was purified by column chromatography to furnish **IBC14** as a yellow solid. Yield: 53%, mp 86–88 °C. FTIR (ATR) υ (cm^−1^): 2918, 2851 (CH_aliphatic_), 1653 (>C=O), 1603, 1589, 1566, 1522, 1472, 1423, 1395, 1371, 1341, 1290, 1256, 1225, 1185, 1113, 1034. ^1^H NMR (600 MHz, *CDCl*_3_) *δ* (ppm): δ 8.05 (d, *J* = 8.4 Hz, 2H, CH_aromatic_), 7.90 (s, 1H, CH_aromatic_), 7.73 (d, *J* = 15.5 Hz, 1H, C_vinyl_), 7.51 (d, *J* = 8.6 Hz, 2H, CH_aromatic_), 7.42 (d, *J* = 8.4 Hz, 2H, CH_aromatic_), 7.32 (d, *J* = 15.6 Hz, 1H, C_vinyl_), 7.28 (s, 1H, CH_aromatic_), 7.16 (s, 1H, CH_aromatic_), 6.84 (d, *J* = 8.6 Hz, 2H, CH_aromatic_), 3.91 (t, *J* = 6.5 Hz, 2H, OCH_2_), 1.76–1.67 (m, 2H, OCH_2_CH_2_), 1.41–1.33 (m, 2H, OCH_2_CH_2_CH_2_), 1.30–1.10 (m, 12H, CH_2_CH_2_), 0.80 (t, *J* = 6.9 Hz, 3H, CH_3_). ^13^C NMR (150 MHz, *CDCl*_3_) *δ* (ppm): 188.78 (>C=O), 161.60, 145.53, 140.22, 137.28, 135.39, 130.86, 130.41, 130.38, 127.12, 120.76, 118.79, 117.82, 115.00 (C_aromatic_ and C_vinyl_), 68.25 (OCH_2_), 31.89, 29.56, 29.55, 29.37, 29.31, 29.15, 26.00, 22.67 (8xCH_2_), 14.12 (CH_3_). HRMS(+ESI) *m*/*z* calcd for C_28_H_35_N_2_O_2_ [M + H]^+^: 431.2699; found: 431.2699.

#### 4.2.15. 1-(4-(1*H*-imidazol-1-yl)phenyl)-3-(2,3-dimethoxyphenyl)prop-2-en-1-one (IBC15)

Following the general procedure by applying 2,3-dimethoxybenzaldehyde (0.1662 g), the crude residue was purified by column chromatography to furnish **IBC15** as a light brown solid. Yield: 45%, mp 159 °C. FTIR (ATR) υ (cm^−1^): 3136, 3081 (CH_aromatic_), 2990, 2926, 2824 (CH_aliphatic_), 1655 (>C=O), 1599, 1570, 1526, 1476, 1425, 1371, 1346, 1290, 1265, 1234, 1219, 1101, 1072, 1057, 1030. ^1^H NMR (600 MHz, *CDCl*_3_) *δ* (ppm): δ 8.08–8.02 (m, 3H, CH_aromatic_ and C_vinyl_), 7.89 (s, *J* = 13.2 Hz, 1H, CH_aromatic_), 7.52 (d, *J* = 15.8 Hz, 1H, C_vinyl_), 7.44 (d, *J* = 8.6 Hz, 2H, CH_aromatic_), 7.29 (s, 1H, CH_aromatic_), 7.20 (d, *J* = 7.7 Hz, 1H, CH_aromatic_), 7.16 (s, 1H, CH_aromatic_), 7.02 (t, *J* = 8.0 Hz, 1H, CH_aromatic_), 6.90 (dd, *J* = 8.1, 1.1 Hz, 1H, CH_aromatic_), 3.81 (s, 3H, OCH_3_), 3.80 (s, 3H, OCH_3_). ^13^C NMR (150 MHz, *CDCl*_3_) *δ* (ppm): 188.09 (>C=O), 152.20, 148.01, 139.36, 139.30, 135.92, 134.37, 129.99, 129.48, 127.78, 123.24, 121.85, 119.71, 118.61, 116.74, 113.44 (C_aromatic_ and C_vinyl_), 60.31, 54.87 (2xOCH_3_). HRMS(+ESI) *m*/*z* calcd for C_20_H_19_N_2_O_3_ [M + H]^+^: 335.1396; found: 335.1395.

#### 4.2.16. 1-(4-(1*H*-imidazol-1-yl)phenyl)-3-(2,4-dimethoxyphenyl)prop-2-en-1-one (IBC16) [51]

Following the general procedure by applying 2,4-dimethoxybenzaldehyde (0.1662 g), the crude residue was purified by column chromatography to furnish **IBC16** as a yellow solid. Yield: 64%, mp 167–169 °C. FTIR (ATR) υ (cm^−1^): 3130 (CH_aromatic_), 2936, 2847 (CH_aliphatic_), 1651 (>C=O), 1605, 1585, 1566, 1520, 1504, 1485, 1456, 1441, 1420, 1373, 1341, 1298, 1271, 1211, 1163, 1119, 1026. ^1^H NMR (600 MHz, *DMSO-d*_6_) *δ* (ppm): δ 8.15–8.12 (m, 2H, CH_aromatic_), 8.09 (d, *J* = 15.7 Hz, 1H, C_vinyl_), 7.97 (s, 1H, CH_aromatic_), 7.59 (d, *J* = 8.6 Hz, 1H, CH_aromatic_), 7.55 (d, *J* = 15.7 Hz, 1H, C_vinyl_), 7.52 (d, *J* = 8.6 Hz, 2H, CH_aromatic_), 7.37 (s, 1H, CH_aromatic_), 7.25 (s, 1H, CH_aromatic_), 6.55 (dd, *J* = 8.6, 2.3 Hz, 1H, CH_aromatic_), 6.49 (d, *J* = 2.3 Hz, 1H, CH_aromatic_), 3.92 (s, 3H, OCH_3_), 3.87 (s, 3H, OCH_3_). ^13^C NMR (150 MHz, *DMSO-d*_6_) *δ* (ppm): 189.53 (>C=O), 163.32, 160.58, 141.30, 140.12, 137.64, 135.44, 131.25, 131.02, 130.39, 120.75, 119.76, 117.82, 116.94, 105.55, 98.50 (C_aromatic_ and C_vinyl_), 55.60, 55.54 (2xOCH_3_). HRMS(+ESI) *m*/*z* calcd for C_20_H_19_N_2_O_3_ [M + H]^+^: 335.1396; found: 335.1396.

#### 4.2.17. 1-(4-(1*H*-imidazol-1-yl)phenyl)-3-(2,5-dimethoxyphenyl)prop-2-en-1-one (IBC17)

Following the general procedure by applying 2,5-dimethoxybenzaldehyde (0.1662 g), the crude residue was purified by column chromatography to furnish **IBC17** as a brown oil. Yield: 67%. FTIR (ATR) υ (cm^−1^): 3119 (CH_aromatic_), 2916, 2849 (CH_aliphatic_), 1655 (>C=O), 1605, 1589, 1520, 1493, 1423, 1302, 1256, 1213, 1179, 1105, 1026. ^1^H NMR (600 MHz, *CDCl*_3_) *δ* (ppm): δ 8.14 (d, *J* = 8.6 Hz, 2H, CH_aromatic_), 8.11 (d, *J* = 15.8 Hz, 1H, C_vinyl_), 7.99 (s, 1H, CH_aromatic_), 7.59 (d, *J* = 15.8 Hz, 1H, C_vinyl_), 7.52 (d, *J* = 8.6 Hz, 2H, CH_aromatic_), 7.38 (t, *J* = 1.2 Hz, 1H, CH_aromatic_), 7.25 (s, 1H, CH_aromatic_), 7.17 (d, *J* = 3.0 Hz, 1H, CH_aromatic_), 6.96 (dd, *J* = 9.0, 3.0 Hz, 1H, CH_aromatic_), 6.89 (d, *J* = 9.0 Hz, 1H, CH_aromatic_), 3.87 (s, 3H, OCH_3_), 3.82 (s, 3H, OCH_3_). ^13^C NMR (150 MHz, *CDCl*_3_) *δ* (ppm): 189.36 (>C=O), 153.53, 153.43, 140.84, 140.26, 137.15, 135.40, 130.87, 130.51, 124.20, 122.42, 120.74, 117.80, 117.51, 113.95, 112.49 (C_aromatic_ and C_vinyl_), 56.11, 55.84 (2xOCH_3_). HRMS(+ESI) *m*/*z* calcd for C_20_H_19_N_2_O_3_ [M + H]^+^: 335.1396; found: 335.1396.

#### 4.2.18. 1-(4-(1*H*-imidazol-1-yl)phenyl)-3-(2,6-dimethoxyphenyl)prop-2-en-1-one (IBC18)

Following the general procedure by applying 2,6-dimethoxybenzaldehyde (0.1662 g), the crude residue was purified by column chromatography to furnish **IBC18** as a yellow solid. Yield: 47 mp 102–103 °C. FTIR (ATR) υ (cm^−1^): 3123 (CH_aromatic_), 2943, 2839 (CH_aliphatic_), 1657 (>C=O), 1607, 1568, 1520, 1474, 1435, 1325, 1285, 1256, 1202, 1175, 1105, 1057, 1028. ^1^H NMR (600 MHz, *CDCl*_3_) *δ* (ppm): δ 8.32 (d, *J* = 15.9 Hz, 1H, C_vinyl_), 8.15–8.11 (m, 2H, CH_aromatic_), 7.99 (d, *J* = 15.9 Hz, 1H, C_vinyl_), 7.96 (s, 1H, CH_aromatic_), 7.52–7.46 (m, 2H, CH_aromatic_), 7.37 (t, *J* = 1.3 Hz, 1H, CH_aromatic_), 7.30 (d, *J* = 8.6 Hz, 1H, CH_aromatic_), 7.23 (s, 1H, CH_aromatic_), 6.59 (d, *J* = 8.4 Hz, 2H, CH_aromatic_), 3.92 (s, 6H, OCH_3_). ^13^C NMR (150 MHz, *CDCl*_3_) *δ* (ppm): 190.47 (>C=O), 160.48, 140.02, 137.66, 136.47, 135.39, 131.89, 130.92, 130.45, 124.14, 120.64, 117.80, 112.69, 103.79 (C_aromatic_ and C_vinyl_), 55.90 (2xOCH_3_). HRMS(+ESI) *m*/*z* calcd for C_20_H_19_N_2_O_3_ [M + H]^+^: 335.1396; found: 335.1396.

#### 4.2.19. 1-(4-(1*H*-imidazol-1-yl)phenyl)-3-(3,5-dimethoxyphenyl)prop-2-en-1-one (IBC19)

Following the general procedure by applying 3,5-dimethoxybenzaldehyde (0.1662 g), the crude residue was purified by column chromatography to furnish **IBC19** as a dark red solid. Yield: 70%, mp 156–158 °C. FTIR (ATR) υ (cm^−1^): 3120 (CH_aromatic_), 2920, 2851 (CH_aliphatic_), 1684, 1682 (>C=O), 1593, 1520, 1456, 1427, 1285, 1204, 1152. ^1^H NMR (600 MHz, *DMSO-d*_6_) *δ* (ppm): δ 8.15 (d, *J* = 8.6 Hz, 2H, CH_aromatic_), 7.99 (s, 1H, CH_aromatic_), 7.76 (d, *J* = 15.6 Hz, 1H, C_vinyl_), 7.54 (d, *J* = 8.6 Hz, 2H, CH_aromatic_), 7.48 (d, *J* = 15.7 Hz, 1H, C_vinyl_), 7.38 (s, 1H, CH_aromatic_), 7.26 (s, 1H, CH_aromatic_), 6.79 (d, *J* = 2.1 Hz, 2H, CH_aromatic_), 6.55 (d, *J* = 2.1 Hz, 1H, CH_aromatic_), 3.85 (s, 6H, OCH_3_). ^13^C NMR (150 MHz, *DMSO-d*_6_) *δ* (ppm): δ 188.86 (>C=O), 161.14, 145.56, 140.50, 136.87, 136.53, 135.42, 131.05, 130.53, 130.06, 121.94, 120.84, 120.81, 120.75, 117.78, 106.54, 106.50, 102.94 (C_aromatic_ and C_vinyl_), 55.51 (OCH_3_). HRMS(+ESI) *m*/*z* calcd for C_20_H_19_N_2_O_3_ [M + H]^+^: 335.1396; found: 335.1378.

#### 4.2.20. 1-(4-(1*H*-imidazol-1-yl)phenyl)-3-(2,4-diethoxyphenyl)prop-2-en-1-one (IBC20)

Following the general procedure by applying 2,4-diethoxybenzaldehyde (0.1942 g), the crude residue was purified by column chromatography to furnish **IBC20** as a yellow solid. Yield: 67%, mp 138–140 °C. FTIR (ATR) υ (cm^−1^): 3111 (CH_aromatic_), 2980, 2932, 2880 (CH_aliphatic_), 1645 (>C=O), 1605, 1593, 1557, 1524, 1481, 1437, 1389, 1306, 1290, 1258, 1219, 1182, 1136, 1107, 1032. ^1^H NMR (600 MHz, *CDCl*_3_) *δ* (ppm): δ 8.14 (d, *J* = 8.6 Hz, 2H, CH_aromatic_), 8.08 (d, *J* = 15.7 Hz, 1H, C_vinyl_), 7.97 (s, 1H, CH_aromatic_), 7.65 (d, *J* = 15.6 Hz, 1H, C_vinyl_), 7.55 (d, *J* = 8.6 Hz, 1H, CH_aromatic_), 7.51 (d, *J* = 8.6 Hz, 2H, CH_aromatic_), 7.37 (s, 1H, CH_aromatic_), 7.25 (s, 1H, CH_aromatic_), 6.52 (dd, *J* = 8.6, 2.2 Hz, 1H, CH_aromatic_), 6.47 (d, *J* = 2.2 Hz, 1H, CH_aromatic_), 4.12 (q, *J* = 7.0 Hz, 1H, OCH_2_), 4.08 (q, *J* = 7.0 Hz, 1H, OCH_2_), 1.53 (t, *J* = 7.0 Hz, 1H, CH_3_), 1.44 (t, *J* = 7.0 Hz, 3H, CH_3_). ^13^C NMR (150 MHz, *CDCl*_3_) *δ* (ppm): 189.39 (>C=O), 162.61, 160.09, 141.71, 140.10, 137.71, 135.43, 131.86, 131.02, 130.31, 120.73, 120.42, 119.57, 117.81, 116.78, 105.96, 99.64 (C_aromatic_ and C_vinyl_), 64.05, 63.78 (2xOCH_2_), 14.82, 14.73 (2xCH_3_). HRMS(+ESI) *m*/*z* calcd for C_22_H_23_N_2_O_3_ [M + H]^+^: 363.1709; found: 363.1708.

#### 4.2.21. 1-(4-(1*H*-imidazol-1-yl)phenyl)-3-(3,4-diethoxyphenyl)prop-2-en-1-one (IBC21)

Following the general procedure by applying 3,4-diethoxybenzaldehyde (0.1942 g), the crude residue was purified by column chromatography to furnish **IBC21** as a yellow solid. Yield: 72%, mp 160–161 °C. FTIR (ATR) υ (cm^−1^): 3123 (CH_aromatic_), 2980, 2878 (CH_aliphatic_), 1651 (>C=O), 1607, 1584, 1564, 1516, 1485, 1423, 1398, 1341, 1304, 1256, 1172, 1101, 1030. ^1^H NMR (600 MHz, *CDCl*_3_) *δ* (ppm): δ 8.14 (d, *J* = 8.6 Hz, 2H, CH_aromatic_), 7.97 (s, 1H, CH_aromatic_), 7.79 (d, *J* = 15.5 Hz, 1H, C_vinyl_), 7.53 (d, *J* = 8.6 Hz, 2H, CH_aromatic_), 7.38 (d, *J* = 15.5 Hz, 1H, C_vinyl_), 7.37 (s, 1H, CH_aromatic_), 7.25 (s, 1H, CH_aromatic_), 7.23 (dd, *J* = 8.3, 1.9 Hz, 1H, CH_aromatic_), 7.19 (d, *J* = 1.9 Hz, 1H, CH_aromatic_), 6.90 (d, *J* = 8.3 Hz, 1H, CH_aromatic_), 4.19–4.13 (m, 4H, 2xOCH_2_), 1.49 (m, 6H, 2xCH_3_). ^13^C NMR (150 MHz, *CDCl*_3_) *δ* (ppm): 188.88 (>C=O), 151.64, 148.88, 145.86, 140.30, 137.25, 135.42, 131.08, 130.40, 127.48, 123.43, 120.77, 119.13, 117.79, 112.76, 112.62 (C_aromatic_ and C_vinyl_), 64.82, 64.53 (2xOCH_2_), 14.82, 14.69 (2xCH_3_). HRMS(+ESI) *m*/*z* calcd for C_22_H_23_N_2_O_3_ [M + H]^+^: 363.1709; found: 363.1708.

#### 4.2.22. 1-(4-(1*H*-imidazol-1-yl)phenyl)-3-(2,3,4-trimethoxyphenyl)prop-2-en-1-one (IBC22)

Following the general procedure by applying 2,3,4-trimethoxybenzaldehyde (0.1962 g), the crude residue was purified by column chromatography to furnish **IBC22** as a yellow solid. Yield: 57%, mp 132–134 °C. FTIR (ATR) υ (cm^−1^): 3123 (CH_aromatic_), 2922 (CH_aliphatic_), 1653 (>C=O), 1607, 1587, 1574, 1524, 1487, 1464, 1416, 1371, 1302, 1275, 1258, 1234, 1190, 1100, 1086, 1043. ^1^H NMR (600 MHz, *CDCl*_3_) *δ* (ppm): δ 8.15 (d, *J* = 8.4 Hz, 2H, CH_aromatic_), 8.04 (d, *J* = 15.7 Hz, 1H, C_vinyl_), 7.99 (s, 1H, CH_aromatic_), 7.57 (d, *J* = 15.7 Hz, 1H, C_vinyl_), 7.53 (d, *J* = 8.4 Hz, 2H, CH_aromatic_), 7.41 (d, *J* = 8.8 Hz, 1H, CH_aromatic_), 7.39 (s, 1H, CH_aromatic_), 7.26 (s, 1H, CH_aromatic_), 6.74 (d, *J* = 8.8 Hz, 1H, CH_aromatic_), 3.97 (s, 3H, OCH_3_), 3.93 (s, 3H, OCH_3_), 3.90 (s, 3H, OCH_3_). ^13^C NMR (150 MHz, *CDCl*_3_) *δ* (ppm): 189.21 (>C=O), 156.09, 153.94, 142.50, 140.91, 140.27, 137.33, 131.04, 130.42, 124.13, 121.75, 120.76, 120.65, 117.82, 107.66 (C_aromatic_ and C_vinyl_), 61.43, 60.93, 56.11 (3xOCH_3_). HRMS(+ESI) *m*/*z* calcd for C_21_H_21_N_2_O_4_ [M + H]^+^: 365.1501; found: 365.1497.

#### 4.2.23. 1-(4-(1*H*-imidazol-1-yl)phenyl)-3-(2,4,5-trimethoxyphenyl)prop-2-en-1-one (IBC23) [52]

Following the general procedure by applying 2,4,5-trimethoxybenzaldehyde (0.1962 g), the crude residue was purified by column chromatography to furnish **IBC23** as a yellow solid. Yield: 64%, mp 193–194 °C. FTIR (ATR) υ (cm^−1^): 3121, 3013 (CH_aromatic_), 2943, 2833 (CH_aliphatic_), 1645 (>C=O), 1605, 1584, 1560, 1508, 1466, 1408, 1371, 1292, 1206, 1179, 1125, 1028. ^1^H NMR (600 MHz, *CDCl*_3_) *δ* (ppm): δ 8.17–8.10 (m, 3H, CH_aromatic_ and C_vinyl_), 7.97 (s, 1H, CH_aromatic_), 7.52 (d, *J* = 8.4 Hz, 2H, CH_aromatic_), 7.47 (d, *J* = 15.7 Hz, 1H, C_vinyl_), 7.37 (d, *J* = 0.8 Hz, 1H, CH_aromatic_), 7.25 (s, 1H, CH_aromatic_), 7.13 (s, 1H, CH_aromatic_), 6.53 (s, 1H, CH_aromatic_), 3.96 (s, 3H, OCH_3_), 3.92 (s, 3H, OCH_3_), 3.91 (s, 3H, OCH_3_). ^13^C NMR (150 MHz, *CDCl*_3_) *δ* (ppm): 189.45 (>C=O), 154.90, 152.85, 143.34, 140.90, 140.12, 137.58, 135.42, 131.04, 130.39, 120.72, 119.62, 117.80, 115.26, 111.64, 96.79 (C_aromatic_ and C_vinyl_), 56.61, 56.34, 56.10 (3xOCH_3_). HRMS(+ESI) *m*/*z* calcd for C_21_H_21_N_2_O_4_ [M + H]^+^: 365.1501; found: 365.1500.

#### 4.2.24. 1-(4-(1*H*-imidazol-1-yl)phenyl)-3-(2,4,6-trimethoxyphenyl)prop-2-en-1-one (IBC24) [53]

Following the general procedure by applying 2,4,6-trimethoxybenzaldehyde (0.1962 g), the crude residue was purified by column chromatography to furnish **IBC24** as a yellow solid. Yield: 57%, mp 188–190 °C. FTIR (ATR) υ (cm^−1^): 3130, 3051 (CH_aromatic_), 2940, 2837 (CH_aliphatic_), 1645 (>C=O), 1605, 1557, 1524, 1456, 1414, 1319, 1292, 1211, 1192, 1157, 1121, 1059, 1030. ^1^H NMR (600 MHz, *CDCl*_3_) *δ* (ppm): δ 8.30 (d, *J* = 15.8 Hz, 1H, C_vinyl_), 8.15–8.11 (m, 2H, CH_aromatic_), 7.96 (s, 1H, CH_aromatic_), 7.88 (d, *J* = 15.8 Hz, 1H, C_vinyl_), 7.50 (d, *J* = 8.6 Hz, 2H, CH_aromatic_), 7.36 (t, *J* = 1.3 Hz, 1H, CH_aromatic_), 7.24 (s, 1H, CH_aromatic_), 6.14 (s, 2H, CH_aromatic_), 3.92 (s, 6H, OCH_3_), 3.87 (s, 3H, OCH_3_). ^13^C NMR (150 MHz, *CDCl*_3_) *δ* (ppm): 190.44 (>C=O), 163.44, 161.88, 139.86, 138.11, 136.75, 135.44, 130.94, 130.33, 121.19, 120.64, 120.10, 117.83, 106.43, 90.57 (C_aromatic_ and C_vinyl_), 55.85, 55.43 (2xOCH_3_). HRMS(+ESI) *m*/*z* calcd for C_21_H_21_N_2_O_4_ [M + H]^+^: 365.1501; found: 365.1473.

#### 4.2.25. 1-(4-(1*H*-imidazol-1-yl)phenyl)-3-(3,4,5-trimethoxyphenyl)prop-2-en-1-one (IBC25) [28]

Following the general procedure by applying 3,4,5-trimethoxybenzaldehyde (0.1962 g), the crude residue was purified by column chromatography to furnish **IBC25** as a yellow solid. Yield: 65%, mp 173 °C. FTIR (ATR) υ (cm^−1^): 3111 (CH_aromatic_), 2924, 2581 (CH_aliphatic_), 1655 (>C=O), 1578, 1503, 1458, 1418, 1312, 1277, 1246, 1215, 1153, 1125, 1063, 1026. ^1^H NMR (600 MHz, *CDCl*_3_) *δ* (ppm): δ 8.16 (d, *J* = 8.6 Hz, 2H, CH_aromatic_), 7.98 (s, 1H, CH_aromatic_), 7.77 (d, *J* = 15.6 Hz, 1H, C_vinyl_), 7.55 (d, *J* = 8.6 Hz, 2H, CH_aromatic_), 7.42 (d, *J* = 15.6 Hz, 1H, C_vinyl_), 7.38 (s, 1H, CH_aromatic_), 7.26 (s, 1H, CH_aromatic_), 6.89 (s, 2H, CH_aromatic_), 3.93 (s, 6H, 2xOCH_3_), 3.92 (s, 3H, OCH_3_). ^13^C NMR (150 MHz, *CDCl*_3_) *δ* (ppm): 188.82 (>C=O), 153.54, 145.70, 140.76, 140.44, 136.97, 135.41, 131.10, 130.49, 130.10, 120.80, 120.74, 117.76, 105.85 (C_aromatic_ and C_vinyl_), 61.02, 56.27 (2xOCH_3_). HRMS(+ESI) *m*/*z* calcd for C_21_H_21_N_2_O_4_ [M + H]^+^: 365.1501; found: 365.1502.

### 4.3. Biological Studies

#### 4.3.1. Determination of Biological Activity

The Clinical and Laboratory Standards Institute-approved broth microdilution technique determined the substances’ MICs [54,55]. The CLSI-compliant preparations consisted of four Gram-negative bacteria (*Pseudomonas aeruginosa* ATCC 27853, *Klebsiella pneumoniae* ATCC 4352, *Escherichia coli* ATCC 25922, and *Proteus mirabilis* ATCC 14153), three Gram-positive bacteria (*Staphylococcus aureus* ATCC 29213, *Staphylococcus epidermidis* ATCC 12228, and *Enterococcus faecalis* ATCC 29212 and three Candida species (*Candida albicans* ATCC 10231, *Candida tropicalis* ATCC 750, and *Candida parapsilosis* ATCC 22019). Stock solutions of the chemicals were made in DMSO. Serial two-fold dilutions from 1250 to 0.06 μg/mL were prepared in Mueller–Hinton Broth for bacteria and RPMI 1640 for the yeast. The MIC was the lowest concentration of analyzed extracts that completely suppressed growth. Three experiments were conducted.

Based on antibiotic activity data, we used the CLSI-recommended broth microdilution method to test **IBC20** and **IBC23** against clinically derived multidrug-resistant *Staphylococcus* spp. bacteria in vitro. The Department of Infectious Diseases and Clinical Microbiology, Faculty of Medicine, Istanbul Medipol University, provided 28 nosocomially acquired multidrug-resistant *Staphylococcus* spp. from blood specimens collected between June and December 2024 for this test. Each strain was identified using API STAPH (bioMérieux, Marcy-l’Étoile, France). All *Staphylococcus* spp. isolates were selected using oxacillin susceptibility to identify methicillin-resistant isolates recognized by CLSI (MIC ≥ 4 ug/mL) [54].

#### 4.3.2. Determination of Time-Kill Curves

Using the time-kill curve (TKC) method [56], the bactericidal and synergistic activity of selected molecules (**IBC20** and **IBC23**) alone or in combination with the traditional antibiotics, levofloxacin and linezolid, which were chosen based on their MIC values were evaluated at 1 × MIC against strains of *S. aureus* ATCC 29213 and *S. epidermidis* ATCC 12228. For the strains under examination, molecule-free controls were also included. Viable counts were assessed at 0, 2, 4, 6, and 24 h after inoculation by subculturing 0.1 mL serial dilutions onto TSA plates. The test tubes containing MHB with and without the molecules (growth control) in a total volume of 10 mL were incubated in a 37 °C calibrated shaking water bath. Every test was run in triplicate. The time–kill assay’s lower limit of detection was 1 log_10_ CFU/mL. A decrease of ≥3 log_10_ CFU/mL from the original inoculum was considered to be the bactericidal activity. The effect of the additive was examined in connection with its more powerful element. When compared to the more active of the two medications when taken alone, synergy and antagonism were defined as a 2 log_10_ decrease or rise in the viable count of the combination after 24 h, respectively [56].

#### 4.3.3. Determination of the Antibiofilm Activity

Biofilm attachment and inhibition of biofilm formation assays were performed as previously described [57,58]. For biofilm attachment, an overnight culture of *MRSA* ATCC 43300 was diluted 1/50 to obtain 1 × 10^6^–1 × 10^7^ CFU/200 mL in TSB supplemented with 1% glucose. Then the strains were added to each well of 96-well tissue culture microtiter plates with 1/10 × MIC of tested molecules. The plates were allowed to incubate for 1, 2, and 4 h at 37 °C. The positive control was the studied strain in the medium alone. After incubation, each well was washed with PBS solution three times, and OD was measured at 595 nm.

For inhibition of the biofilm formation, the tested strain was incubated in its medium and molecules at 1× and 1/10× in addition to 1/100 × MIC at 37 °C for 24 h in microtiter plates. Six wells were used for each molecule. The positive control was the tested strain in its medium without molecules. After incubation, each well was washed with PBS solution thrice, and OD was measured at 595 nm.

#### 4.3.4. Statistical Analysis

All studies were conducted in three separate assays. Two-way ANOVA, Tukey’s multiple comparison test was performed to analyze differences between the control group and molecules alone or in combination with levofloxacin or linezolid. A *p* value of less than 0.0001 was judged statistically significant.

### 4.4. Molecular Docking, In Silico Drug-likeness, and ADMET Analysis

The X-ray crystallographic structures of *S. epidermidis* TcaR in its apo form and in complex with beta-lactam antibiotics (PDB code: 3KP4) [46] and *S. epidermidis* bacterial teichoic acid polymerase TagF (PDB code: 3L7L) [45] were retrieved from the Protein Data Bank to serve as a model in this study. AutoDock Vina 1.2.3 was utilised for the docking studies on imidazole-based chalcones [59,60]. Before initializing the molecular docking process, for 3KP4, chain A and for 3L7L, chain D were selected, water residues and heteroatoms were removed, polar hydrogens and Gasteiger charges were added to the protein molecules. The binding sites were defined according to the coordinates of co-crystallized ligands (methicillin and Cytidine 5′-diphosphoglycerol for 3KP4 and 3L7L, respectively). ChemDraw Professional 16.0 was used to draw 2D chemical structures of the ligands and 3D conversion and energy minimization were performed on Avogadro 1.2.0 software [61] by applying the MMFF94 force field. The visualization of molecular interactions was conducted using Biovia Discovery Studio Visualizer v.21.1.0 [62].

To validate the docking procedure, the co-crystallized methicillin ligand from the TcaR crystal structure (PDB ID: 3KP4) and Cytidine 5′-diphosphoglycerol from TagF (PDB ID: 3L7L) was re-docked into its native binding sites using the same grid parameters and docking settings as for test compounds. The resulting pose was superimposed on the experimental ligand, and the root-mean-square deviation (RMSD) was used to assess docking accuracy.

The SwissADME (https://swissadme.ch; accessed on 7 November 2025) online tool [63] was utilised to assess drug-likeness and predict the ADME (Absorption, Distribution, Metabolism, and Excretion) parameters of the ligands.

## Figures and Tables

**Table 1:** In vitro antimicrobial activity results of the IBCs (**IBC1-25**) ^a^.

IDs		Microorganisms									
		Gram-Negative Bacteria (MIC, μg/mL)				Gram-Positive Bacteria (MIC, μg/mL)			Fungi (MIC, μg/mL)		
		*P. aeruginosa*	*E. coli*	*K. pneumoniae*	*P. mirabilis*	*S. aureus*	*S. epidermidis*	*E. faecalis*	*C. albicans*	*C. parapsilosis*	*C. tropicalis*
Monoalkoxy Substituted	IBC1	-	-	-	-	-	-	-	-	-	-
	IBC2	-	-	-	-	-	-	-	-	-	-
	IBC3	-	-	-	-	-	-	-	312.50	-	-
	IBC4	-	-	-	-	-	-	-	312.50	-	-
	IBC5	625	312.50	-	-	19.53	39.06	-	156.25	78.12	-
	IBC6	-	-	-	-	-	-	-	-	-	-
	IBC7	-	-	-	-	-	-	-	-	-	-
	IBC8	-	-	-	-	-	-	-	-	-	-
	IBC9	-	-	-	-	-	-	-	-	-	-
	IBC10	-	-	-	-	-	-	-	312.50	625	-
	IBC11	-	-	-	-	-	-	-	-	-	-
	IBC12	-	-	-	-	-	-	-	-	-	-
	IBC13	-	-	-	-	-	-	-	-	-	-
	IBC14	-	-	-	-	-	-	-	-	-	-
Dialkoxy Substituted	IBC15	-	625	-	-	-	-	-	-	-	-
	IBC16	-	-	-	-	-	-	-	-	-	-
	IBC17	-	-	-	-	-	-	-	156.25	-	-
	IBC18	625	312.50	-	-	39.06	39.06	-	156.25	156.25	-
	IBC19	-	-	-	-	125	-	-	-	-	-
	IBC20	-	312.50	625	-	39.06	9.76	-	156.25	78.12	156.25
	IBC21	625	625	-	-	39.06	39.06	-	156.25	156.25	39.06
Trialkoxy Substituted	IBC22	-	-	-	-	625	-	-	312.50	625	-
	IBC23	312.50	156.25	312.50	-	78.12	19.53	312.50	156.25	78.12	-
	IBC24	-	625	-	-	19.53	156.25	-	156.25	78.12	39.06
	IBC25	-	-	-	-	250	-	-	-	-	-
Reference Drugs	Ceftazidime	2.44	-	-	-	-	-	-	-	-	-
	Cefuroxime-Na	-	4.88	4.88	2.44	1.22	-	-	-	-	-
	Cefuroxime	-	-	-	-	-	9.76	-	-	-	-
	Amikacin	-	-	-	-	-	-	128	-	-	-
	Clotrimazole	-	-	-	-	-	-	-	4.88	-	-
	Amphotericin B	-	-	-	-	-	-	-	-	0.5	1

^a^ “-“ means no activity.

**Table 2:** Molecular docking study of **IBC20** and **IBC23** with *S. epidermidis* TcaR and TagF.

Code	Parameter	*S. epidermidis* TcaR PDB ID: 3KP4	*S. epidermidis* Wall Teichoic Acid Polymerase TagF PDB ID: 3L7L
IBC20	Estimated free energy of binding (kcal/mol)	−8.1	−7.6
IBC23	Estimated free energy of binding (kcal/mol)	−7.9	−7.7
Cefuroxime	Estimated free energy of binding (kcal/mol)	−8.1	−7.7

**Table 3:** ADME Prediction of **IBC20** and **IBC23**.

Property	IBC20	IBC23
MW	362	364
TPSA (Å^2^)	53.35	62.58
HBA	4	5
HBD	0	0
XlogP3	4.21	3.35
LogS	−4.62	−4.21
GI Absorption	High	High
BBB permanent	Yes	Yes
Pgp substrate	No	No
Lipinski	Yes (0 violation)	Yes (0 violation)
Ghose	Yes	Yes
Veber	Yes	Yes
Egan	Yes	Yes
Muegge	Yes	Yes
Bioavailability score	0.55	0.55
CYP1A2, CYP2C19, CYP2C9, CYP2D6, CYP3A4	Yes, Yes, Yes, Yes, Yes	Yes, Yes, Yes, No, Yes

## Data Availability

The raw data supporting the conclusions of this article will be made available by the authors on request.

## Supplementary Materials

The following supporting information can be downloaded at: https://www.mdpi.com/article/10.3390/antibiotics15030310/s1.
