# Supplementary material for: Microwave-Assisted Synthesis of Imidazole-Based Chalcones: Modulating Antimicrobial Activity Through Alkoxy Substitutions
Source: Antibiotics (Basel). 2026 Mar 18;15(3):310. doi: 10.3390/antibiotics15030310 (PMC13024498; doi:10.3390/antibiotics15030310)
Supplement: Supplementary file 1 [file antibiotics-15-00310-s001.zip › antibiotics-4171767-supplementary.pdf]

Microwave-Assisted Synthesis of Imidazole-Based Chalcones: Modulating Antimicrobial Activity through Alkoxy Substitutions

Elnar Mammadov [a, b], Nilüfer Bayrak [a], Emel Mataracı-Kara [c], and Amaç Fatih TuYuN [a, *]

[a] Department of Chemistry, Faculty of Science, Istanbul University, Fatih, 34134, Istanbul, Türkiye

[b] Institute of Graduate Studies in Science, Istanbul University, Fatih, 34134, Istanbul, Türkiye

[c] *Department of Pharmaceutical Microbiology, Pharmacy Faculty, Istanbul University, Beyazit, 34116, Istanbul, Turkey*

* Author to whom correspondence should be addressed; E-Mail: aftuyun@gmail.com, aftuyun@istanbul.edu.tr (A. F. T.).

Tel.: +90212 440 0000.

Contents

Docking Studies of imidazole-based chalcones (s) analogues S3-4

^{1}H and ^{13}C spectra of imidazole-based chalcones (s) analogues (**1-25**) S4-29

HRMS spectra of imidazole-based chalcones (s) analogues (**1-25**) S30-36

FTIR spectra of imidazole-based chalcones (s) analogues (**1-25**) S37-45

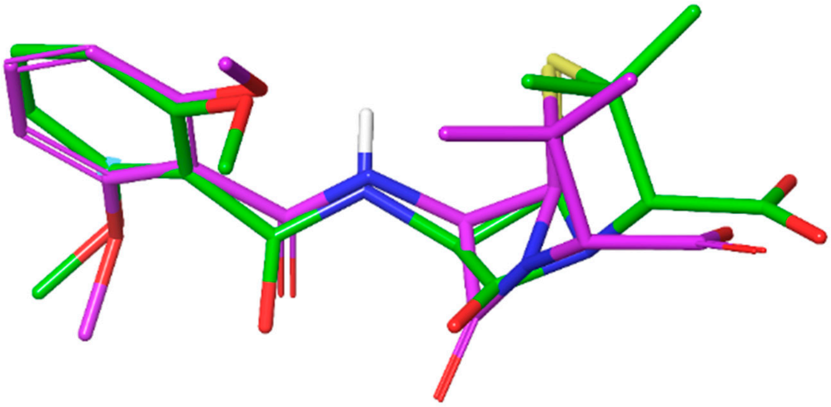

Figure S1. Superimposition of co-crystallized and docked methicillin in TcaR (PDB ID: 3KP4). Superimposed view of the co-crystallized methicillin (shown in purple) and the re-docked methicillin pose (shown in green) within the TcaR binding pocket. The close structural overlap indicates a high docking accuracy and reliable reproduction of the experimental binding conformation. RMSD value: 0.7585.

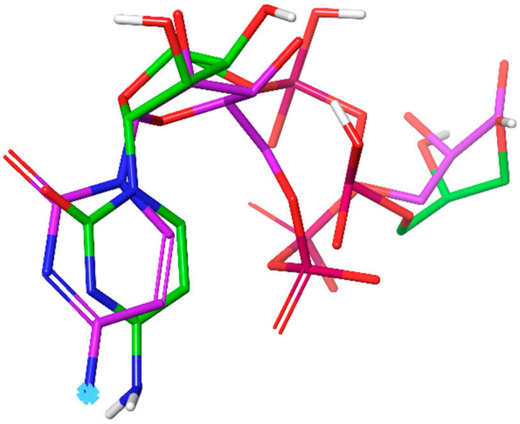

Figure S2. Superimposition of co-crystallized and docked Cytidine 5'-diphosphoglycerol in TagF (PDB ID: 3L7L). Superimposed view of the co-crystallized ligand (shown in magenta) and the re-docked ligand pose (shown in green) within the TagF binding pocket. The close structural overlap indicates a high docking accuracy and reliable reproduction of the experimental binding conformation. RMSD value: 1.2613.

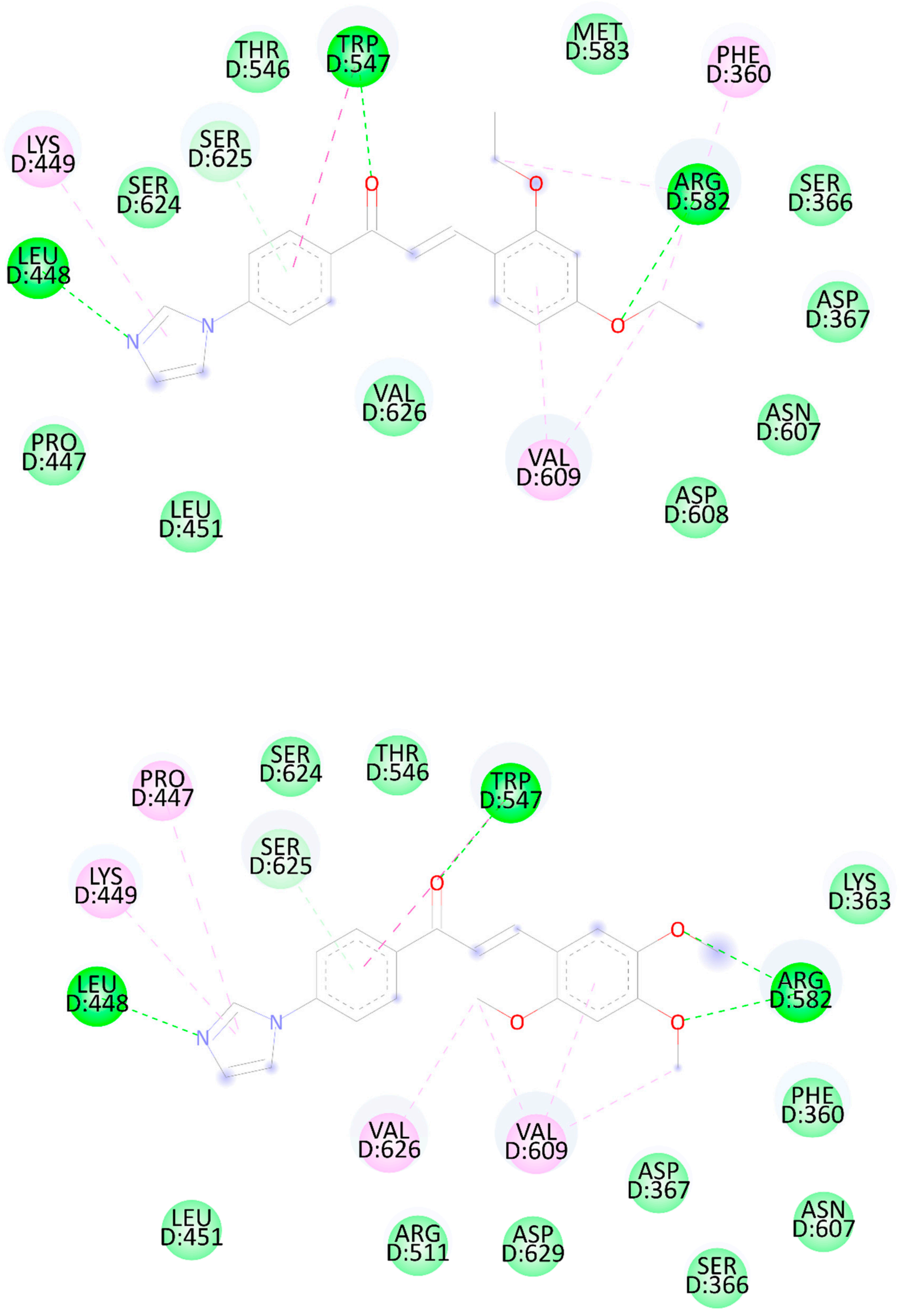

Figure S3. A 2D-interaction plot of **20** and **23** with TagF (PDB: 3L7L). Interaction types: #Hydrogen Bond; #van der Waals; #Pi-Alkyl; #Pi-Pi stacked or Amide-Pi stacked.

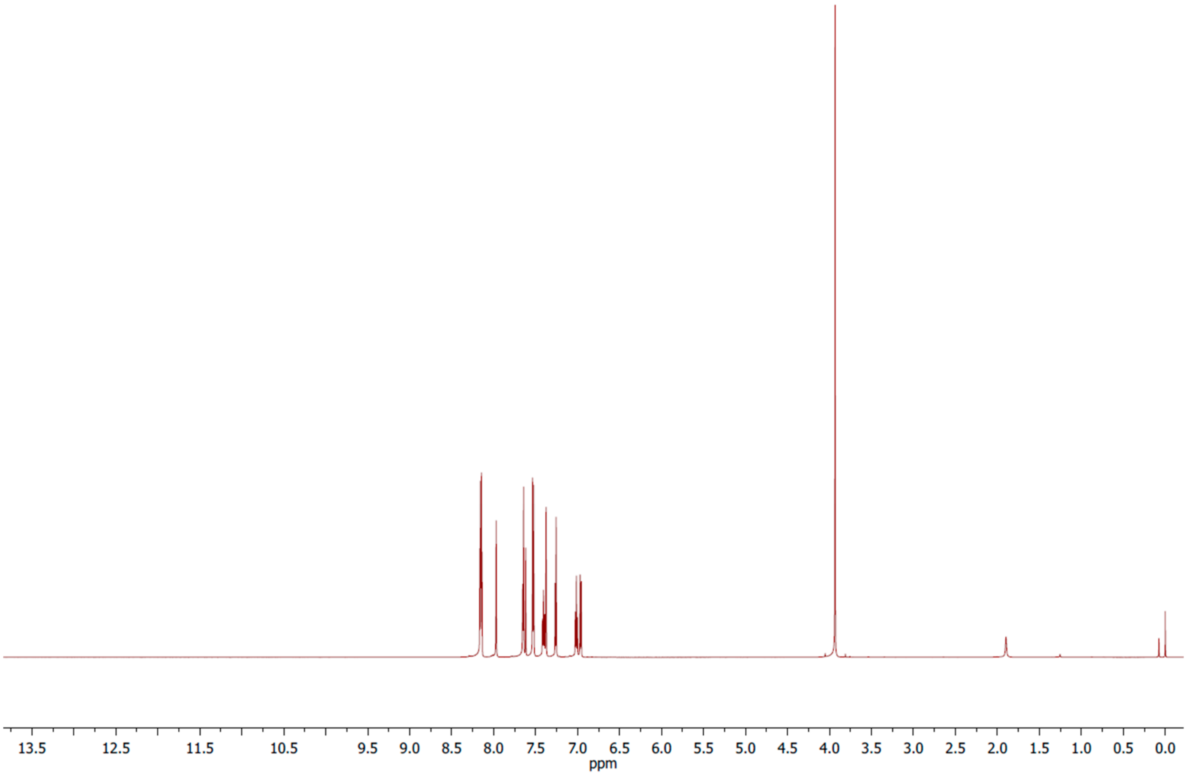

Figure S4. ^{1}H NMR (600 MHz) spectrum of the **IBC1** in $CDCl_3$-*d1*

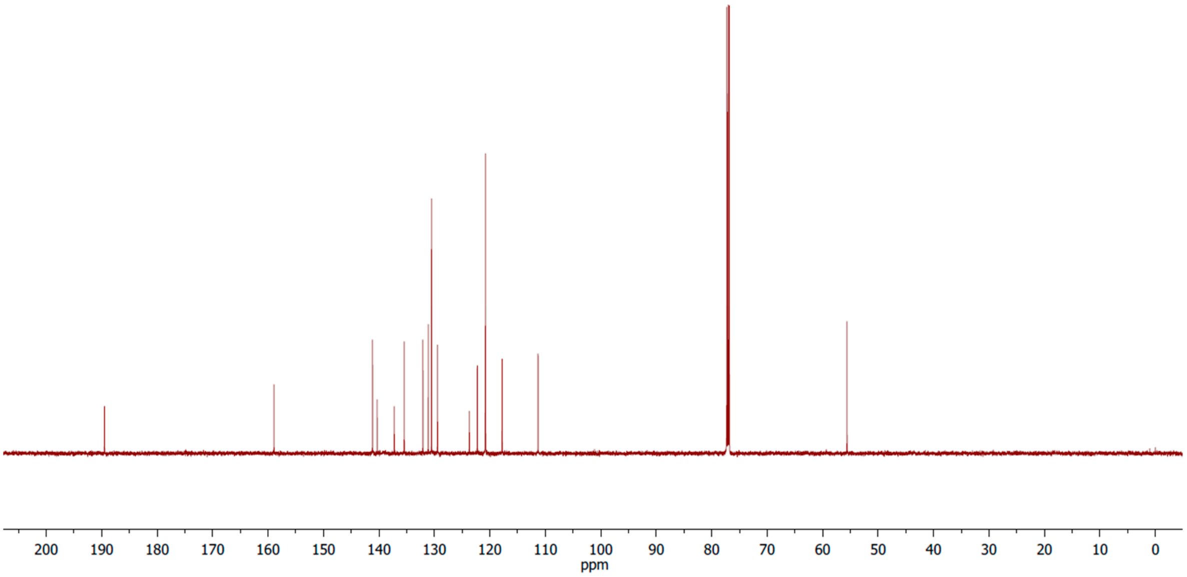

Figure S5. ^{13}C NMR (151 MHz) spectrum of the **IBC1** in $CDCl_3$-*d1*

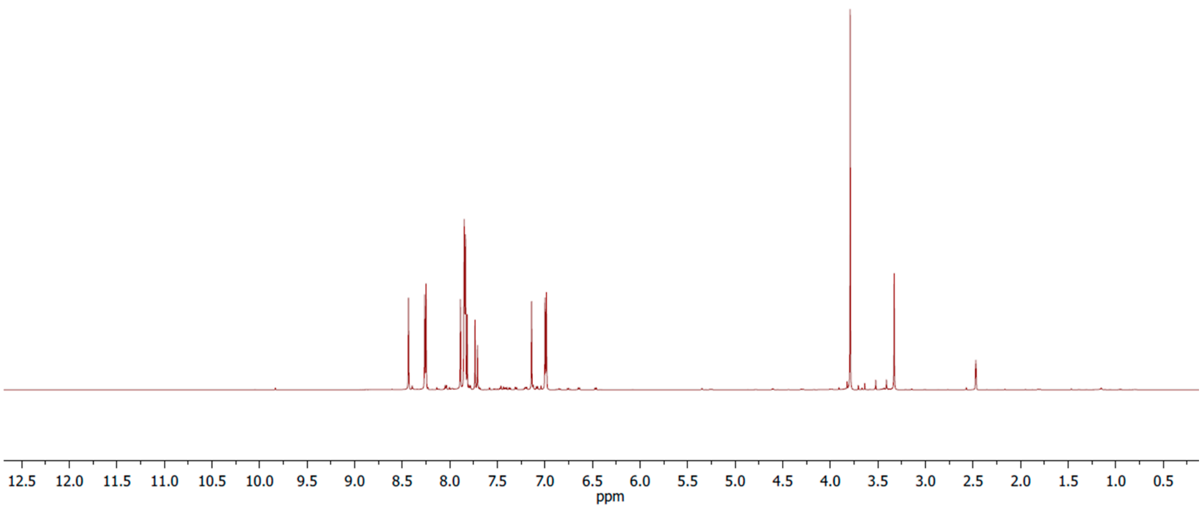

Figure S6. ^{1}H NMR (600 MHz) spectrum of the **IBC2** in DMSO-d_6

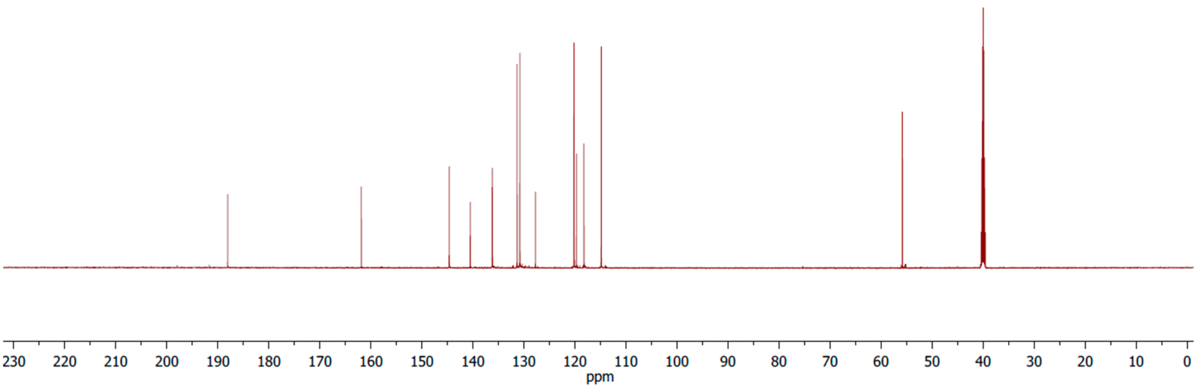

Figure S7. ^{13}C NMR (151 MHz) spectrum of the **IBC2** in DMSO-d_6

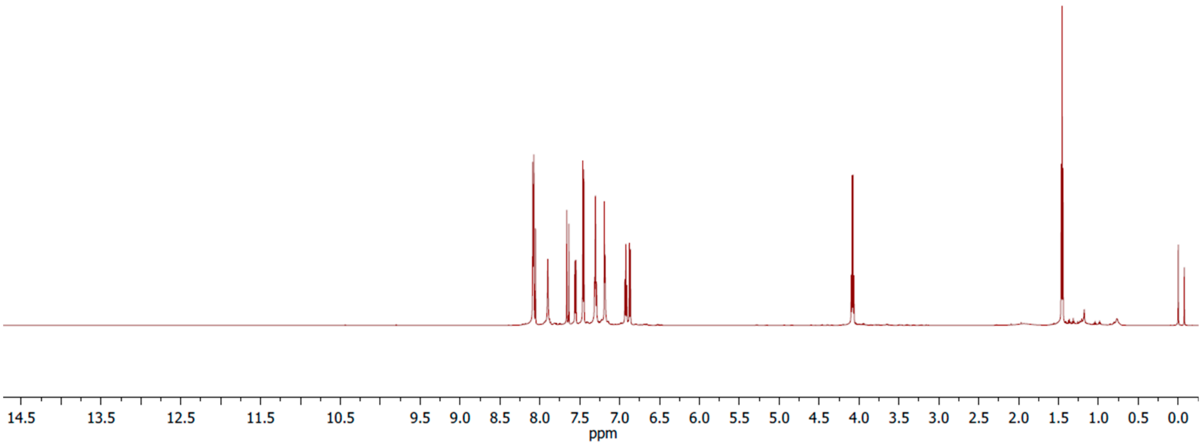

Figure S8. ^{1}H NMR (600 MHz) spectrum of the **IBC3** in $CDCl_3$-*d1*

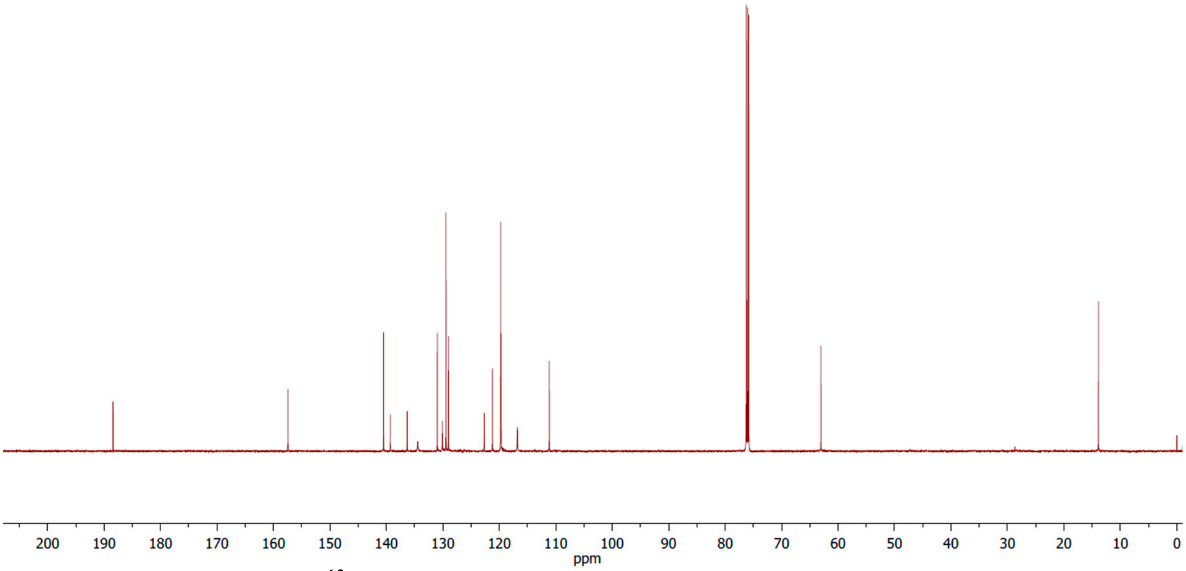

Figure S9. ^{13}C NMR (151 MHz) spectrum of the **IBC3** in $CDCl_3$-*d1*

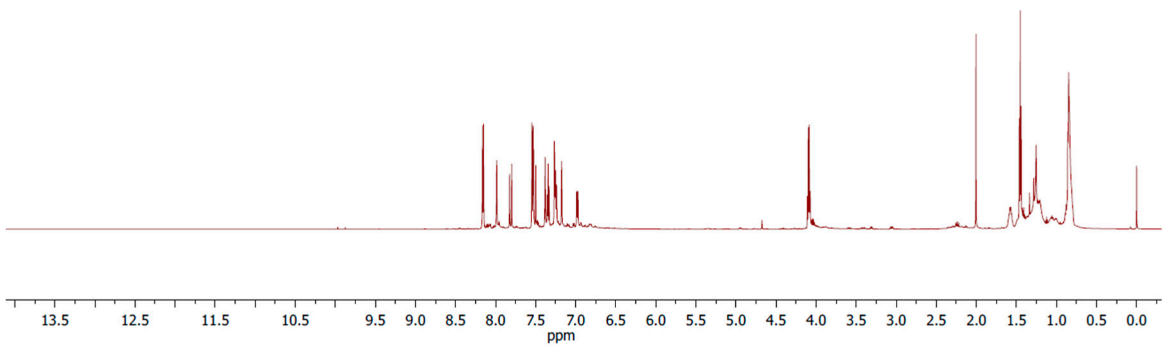

Figure S10. ^{1}H NMR (600 MHz) spectrum of the **IBC4** in $CDCl_3$-*d1*

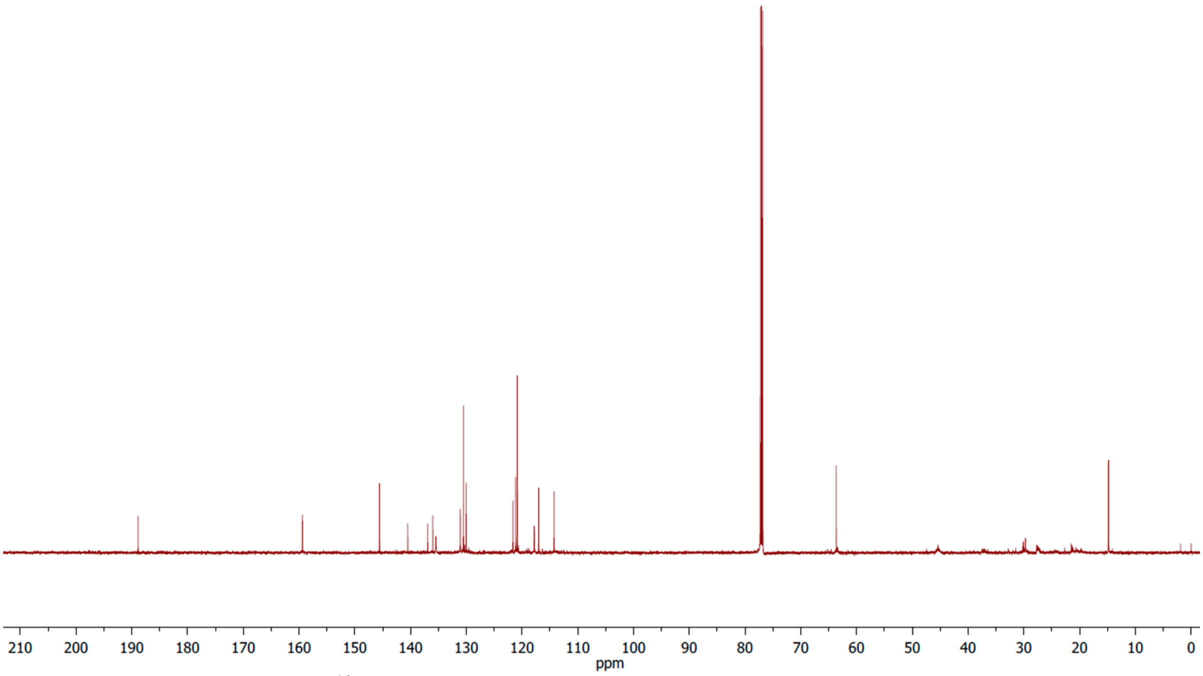

Figure S11. ^{13}C NMR (151 MHz) spectrum of the **IBC4** in $CDCl_3$-*d1*

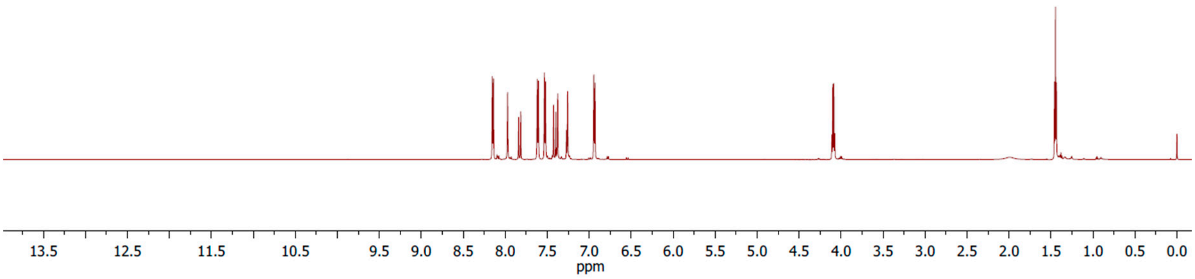

Figure S12. ^{1}H NMR (600 MHz) spectrum of the **IBC5** in $CDCl_3$-*d1*

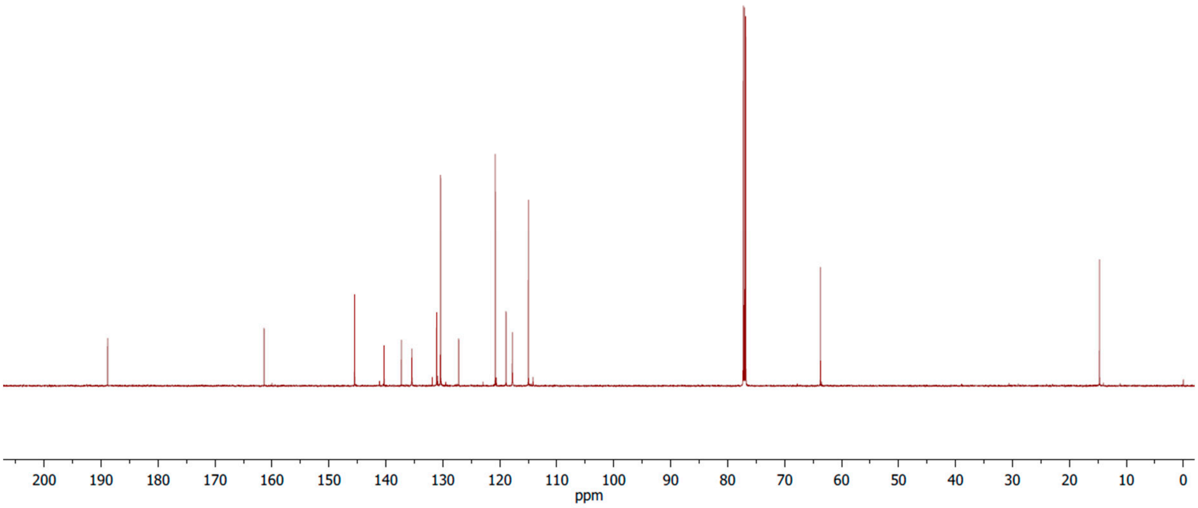

Figure S13. ^{13}C NMR (151 MHz) spectrum of the **IBC5** in $CDCl_3$-*d1*

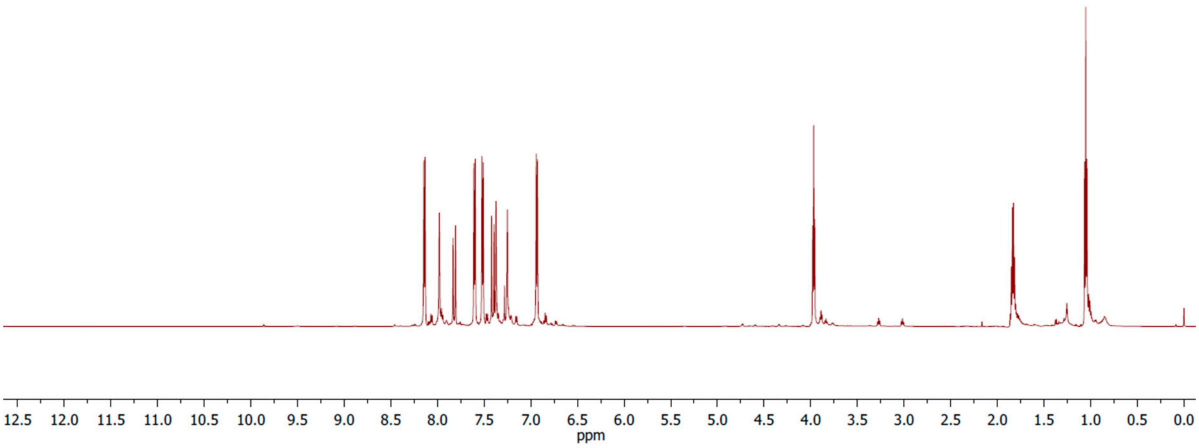

Figure S14. ^{1}H NMR (600 MHz) spectrum of the **IBC6** in $CDCl_3$-*d1*

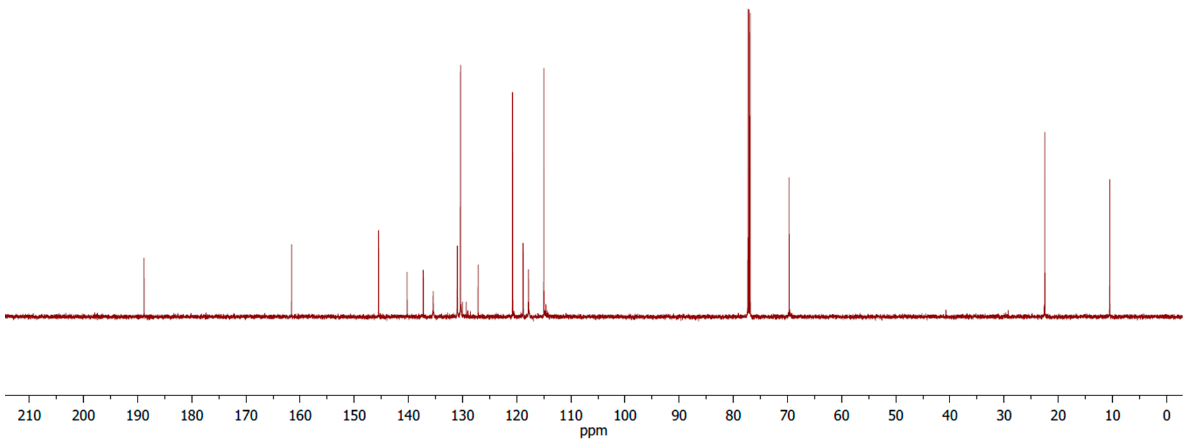

Figure S15. ^{13}C NMR (151 MHz) spectrum of the **IBC6** in $CDCl_3$-*d1*

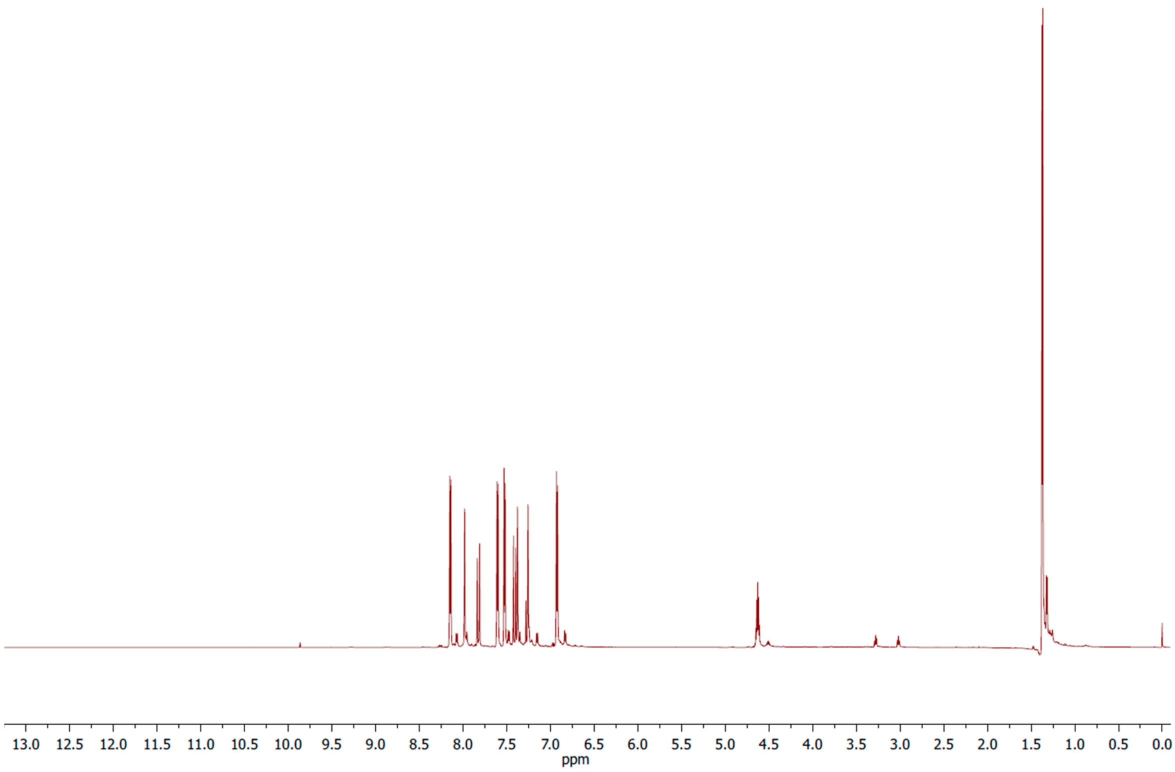

Figure S16. ^{1}H NMR (600 MHz) spectrum of the **IBC7** in $CDCl_3$-*d1*

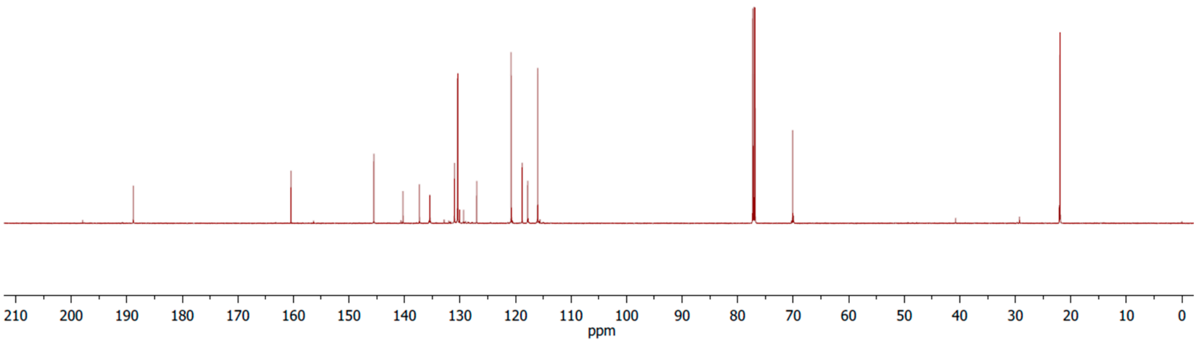

Figure S17. ^{13}C NMR (151 MHz) spectrum of the **IBC7** in $CDCl_3$-*d1*

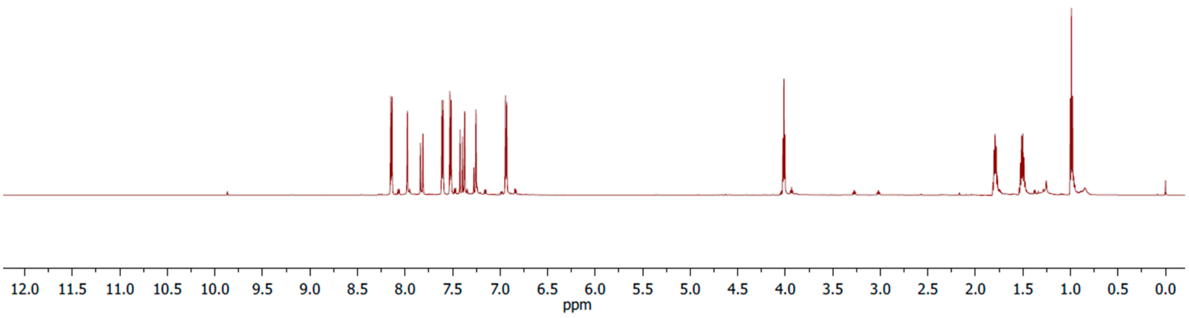

Figure S18. ^{1}H NMR (600 MHz) spectrum of the **IBC8** in $CDCl_3$-*d1*

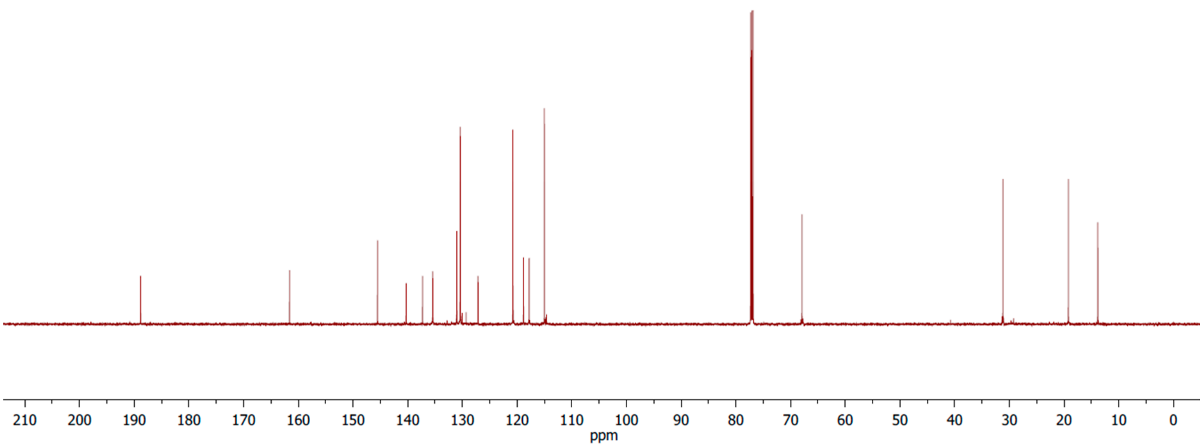

Figure S19. ^{13}C NMR (151 MHz) spectrum of the **IBC8** in $CDCl_3$-*d1*

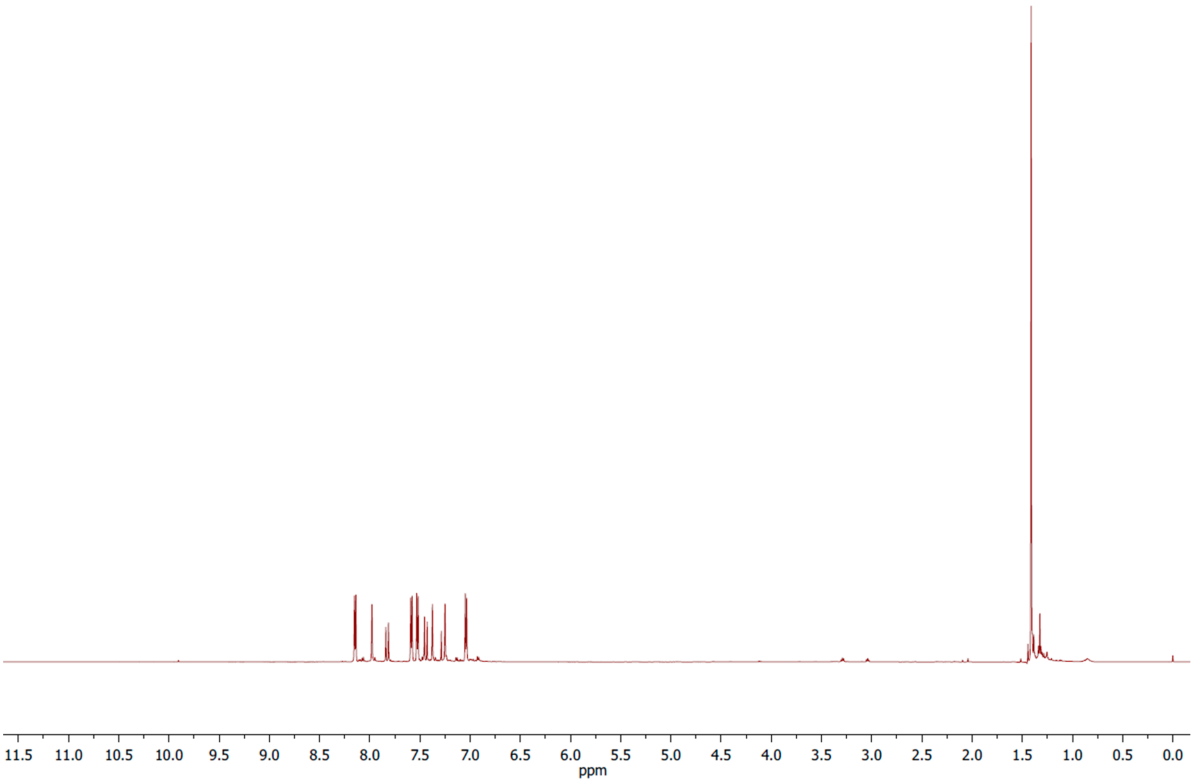

Figure S20. ^{1}H NMR (600 MHz) spectrum of the **IBC9** in $CDCl_3$-*d1*

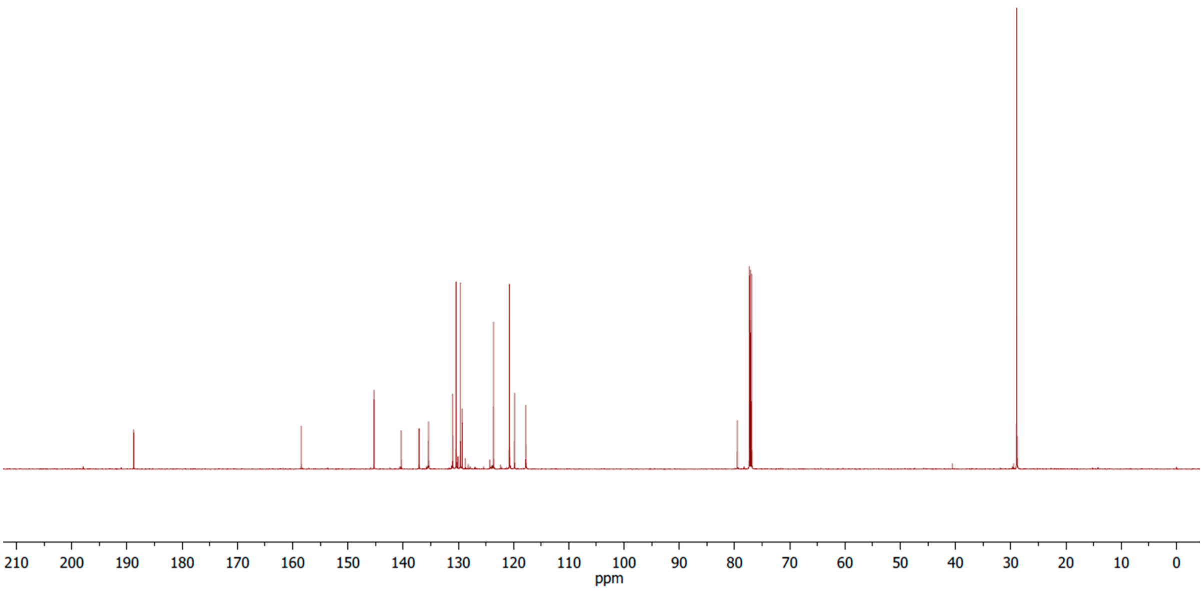

Figure S21. ^{13}C NMR (151 MHz) spectrum of the **IBC9** in $CDCl_3$-*d1*

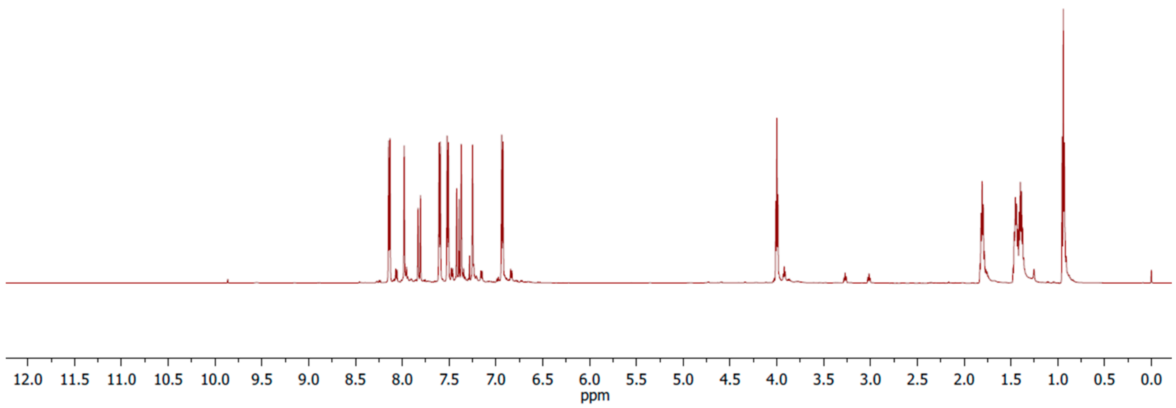

Figure S22. ^{1}H NMR (600 MHz) spectrum of the **IBC10** in $CDCl_3$-*d1*

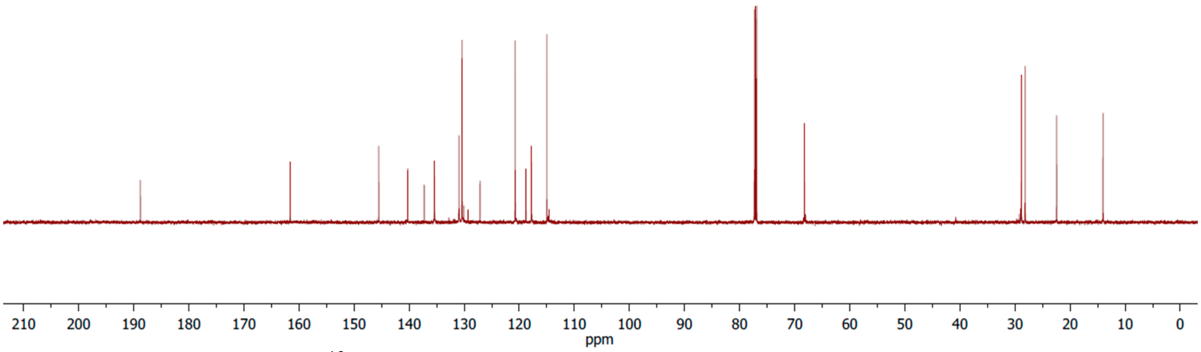

Figure S23. ^{13}C NMR (151 MHz) spectrum of the **IBC10** in $CDCl_3$-*d1*

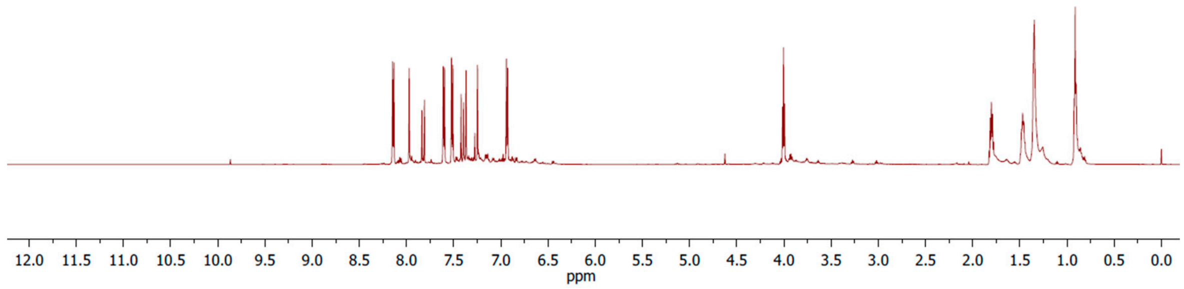

Figure S24. ^{1}H NMR (600 MHz) spectrum of the **IBC11** in $CDCl_3$-*d1*

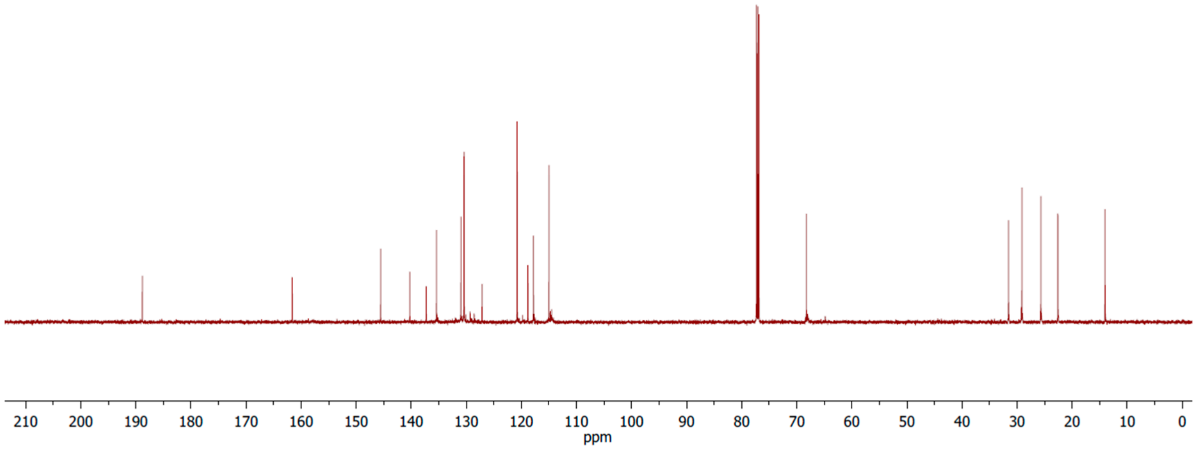

Figure S25. ^{13}C NMR (151 MHz) spectrum of the **IBC11** in $CDCl_3$-*d1*

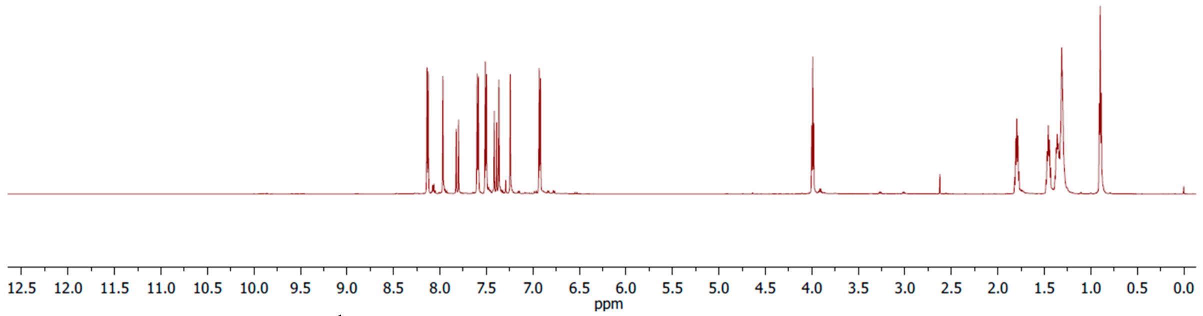

Figure S26. ^{1}H NMR (600 MHz) spectrum of the **IBC12** in $CDCl_3$-*d1*

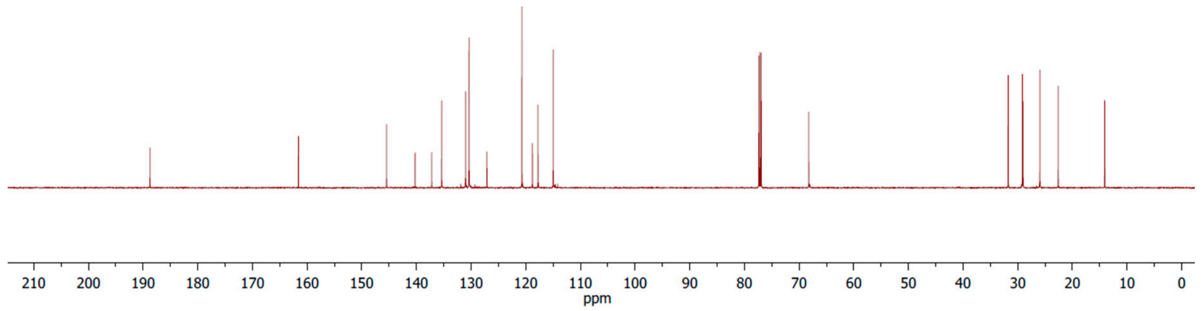

Figure S27. ^{13}C NMR (151 MHz) spectrum of the **IBC12** in $CDCl_3$-*d1*

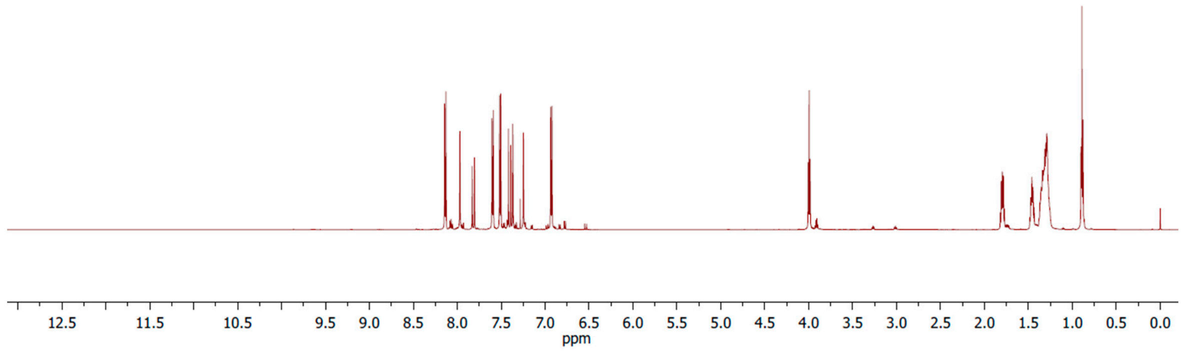

Figure S28. ^{1}H NMR (600 MHz) spectrum of the **IBC13** in $CDCl_3$-*d1*

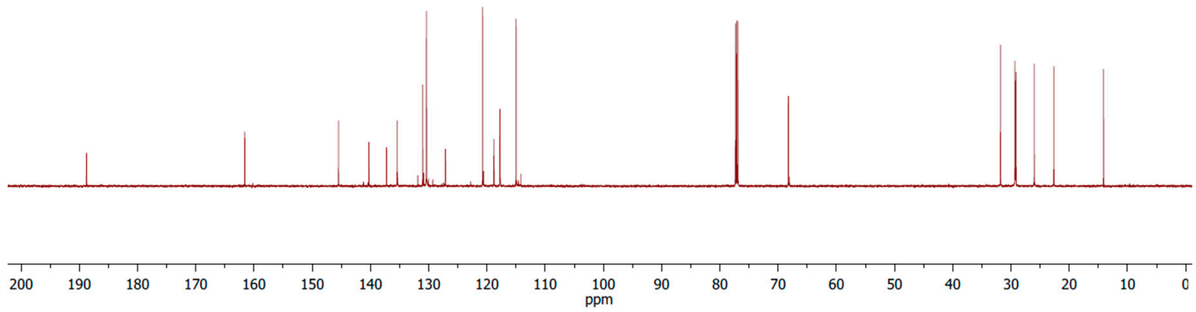

Figure S29. ^{13}C NMR (151 MHz) spectrum of the **IBC13** in $CDCl_3$-*d1*

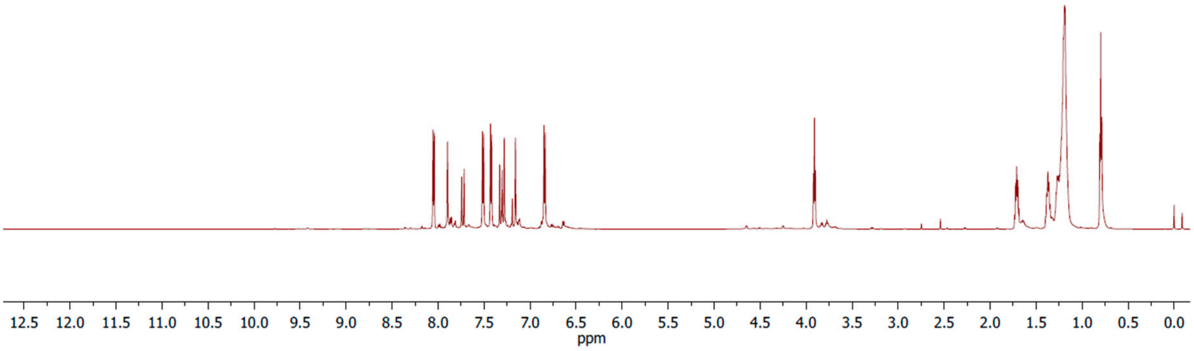

Figure S30. ^{1}H NMR (600 MHz) spectrum of the **IBC14** in $CDCl_3$-*d1*

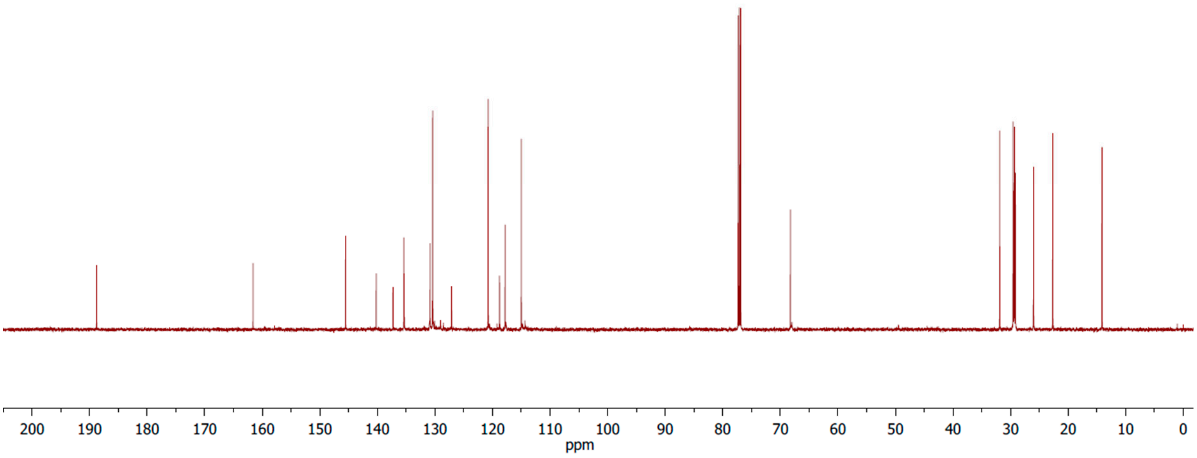

Figure S31. ^{13}C NMR (151 MHz) spectrum of the **IBC14** in $CDCl_3$-*d1*

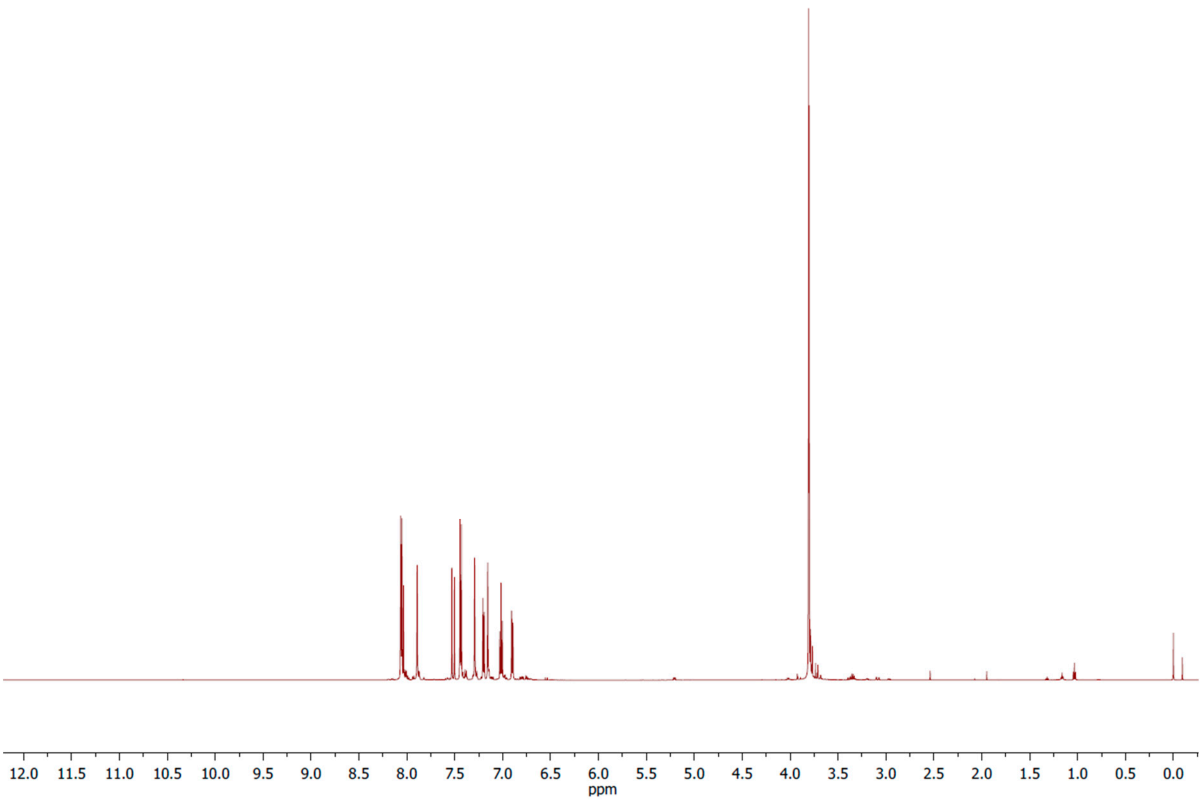

Figure S32. ^{1}H NMR (600 MHz) spectrum of the **IBC15** in $CDCl_3$-*d1*

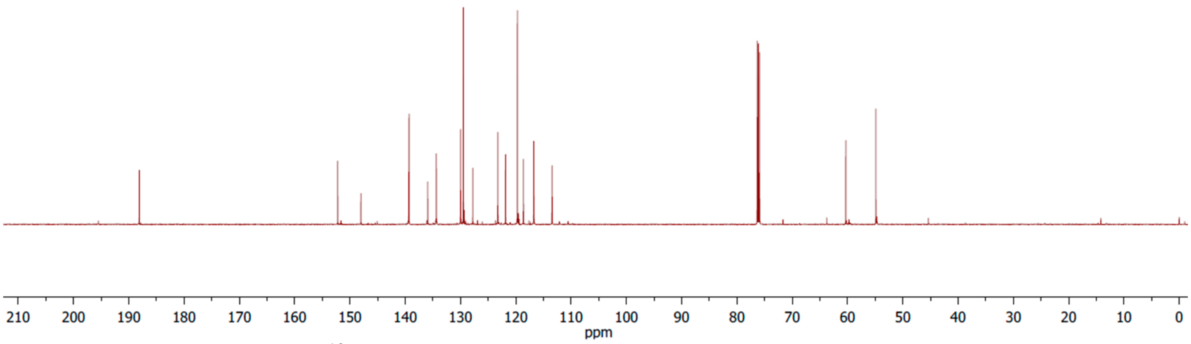

Figure S33. ^{13}C NMR (151 MHz) spectrum of the **IBC15** in $CDCl_3$-*d1*

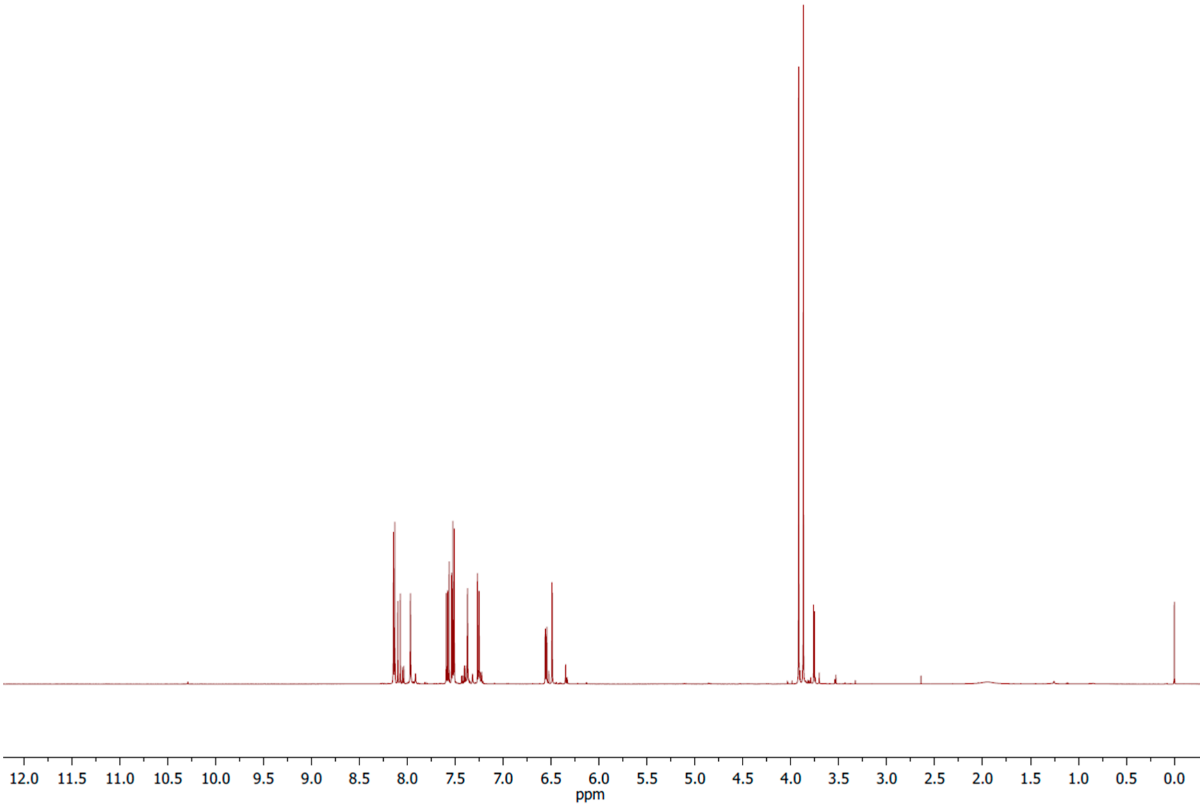

Figure S34. ^{1}H NMR (600 MHz) spectrum of the **IBC16** in $CDCl_3$-*d1*

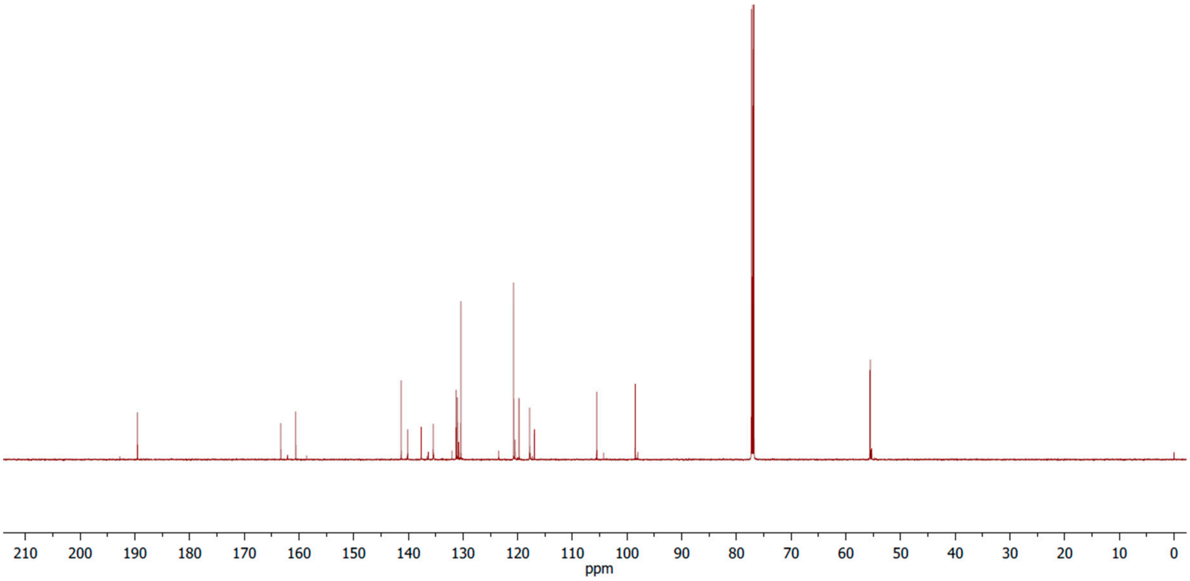

Figure S35. ^{13}C NMR (151 MHz) spectrum of the **IBC16** in $CDCl_3$-*d1*

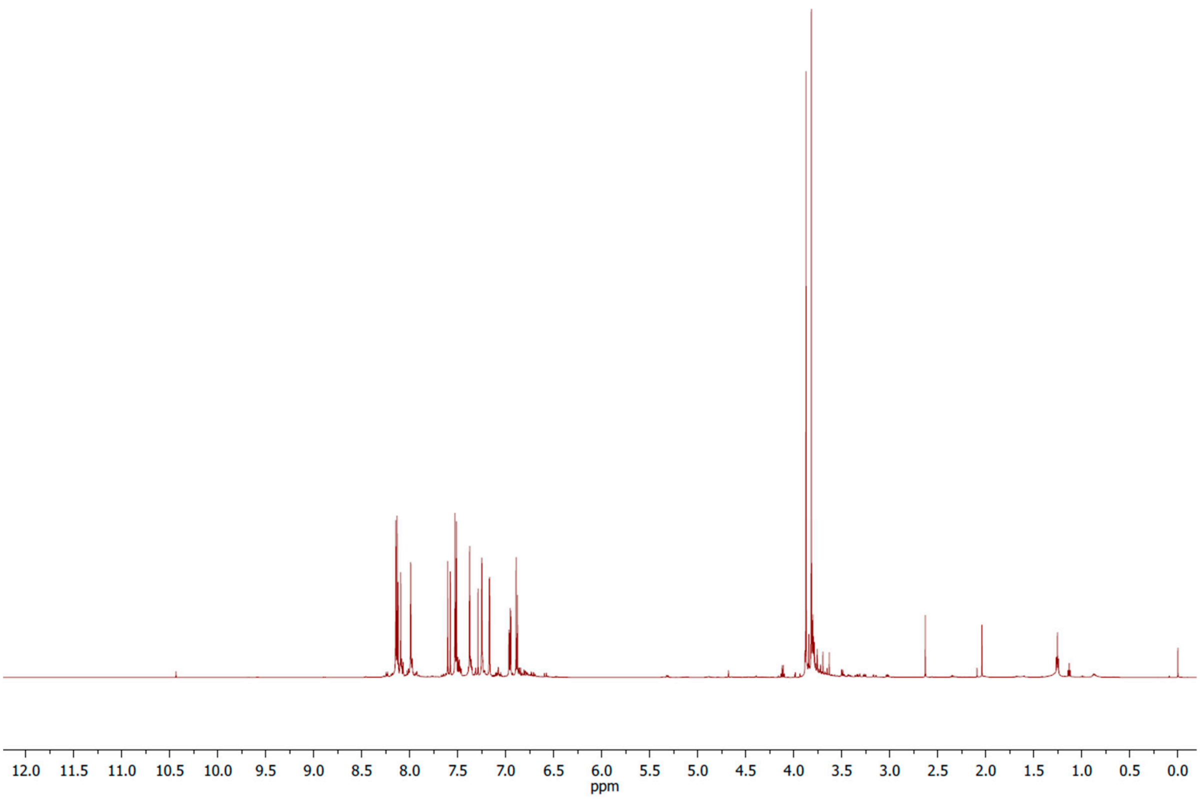

Figure S36. ^{1}H NMR (600 MHz) spectrum of the **IBC17** in $CDCl_3$-*d1*

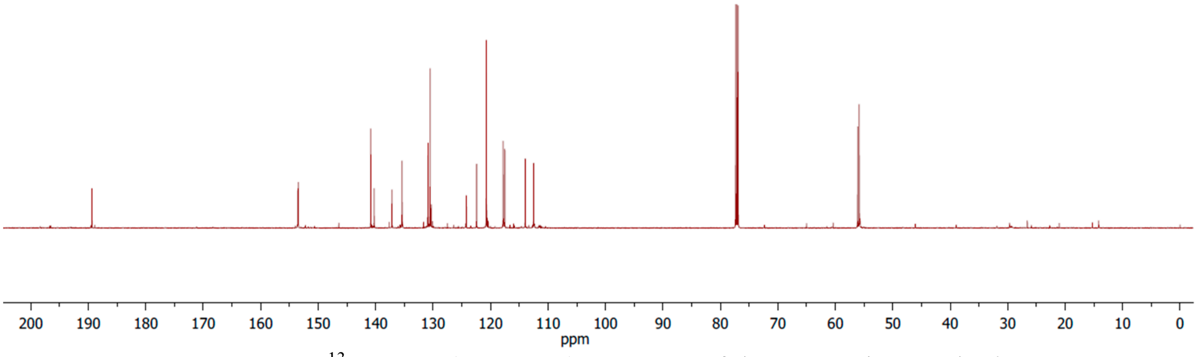

Figure S37. ^{13}C NMR (151 MHz) spectrum of the **IBC17** in $CDCl_3$-*d1*

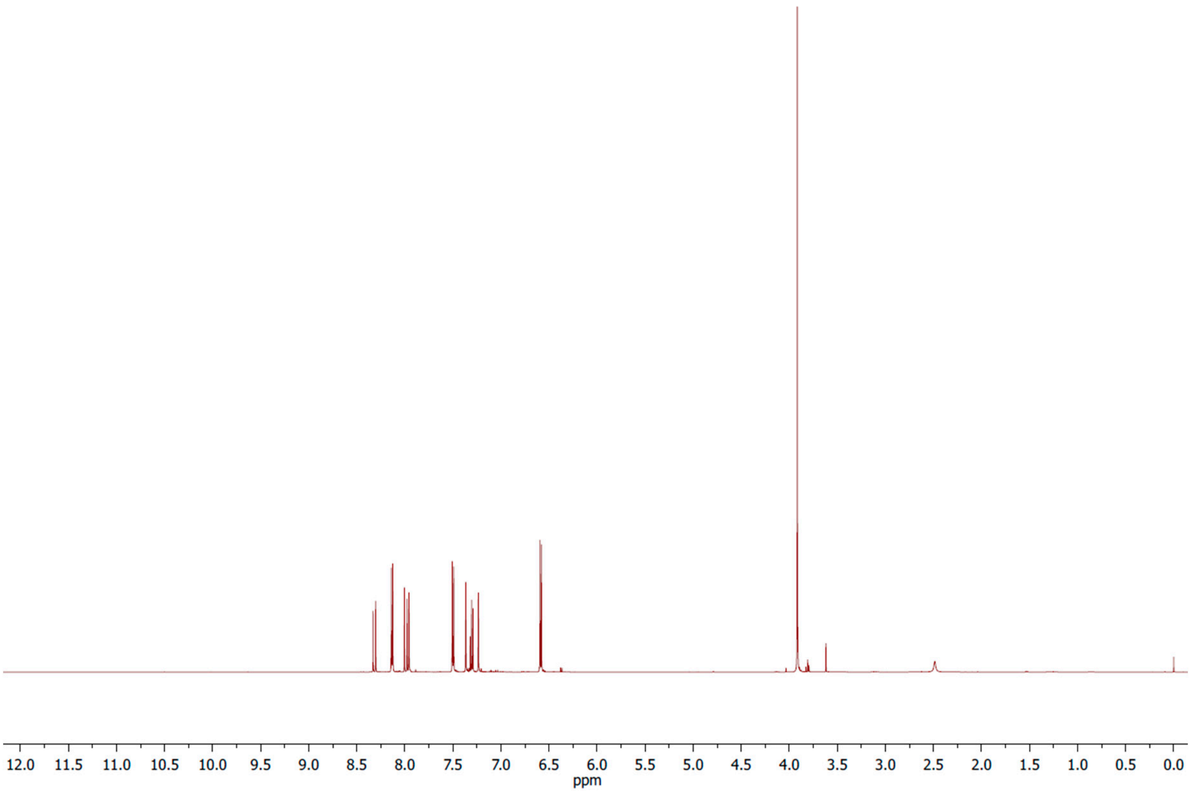

Figure S38. ^{1}H NMR (600 MHz) spectrum of the **IBC18** in $CDCl_3$-*d1*

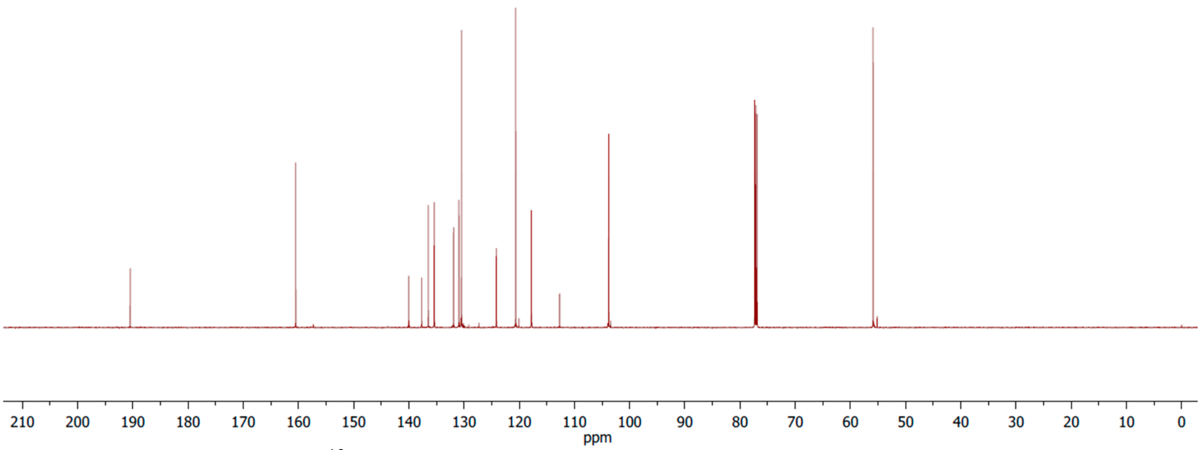

Figure S39. ^{13}C NMR (151 MHz) spectrum of the **IBC18** in $CDCl_3$-*d1*

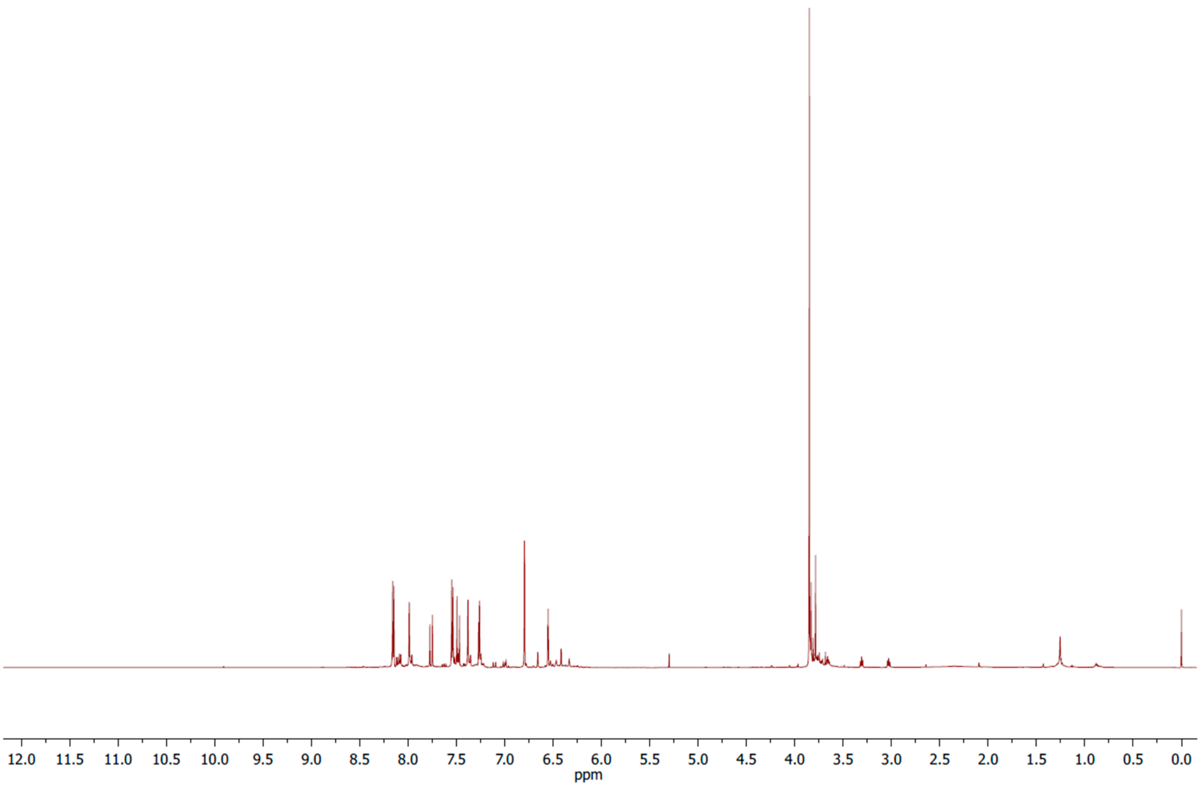

Figure S40. ^{1}H NMR (600 MHz) spectrum of the **IBC19** in $CDCl_3$-*d1*

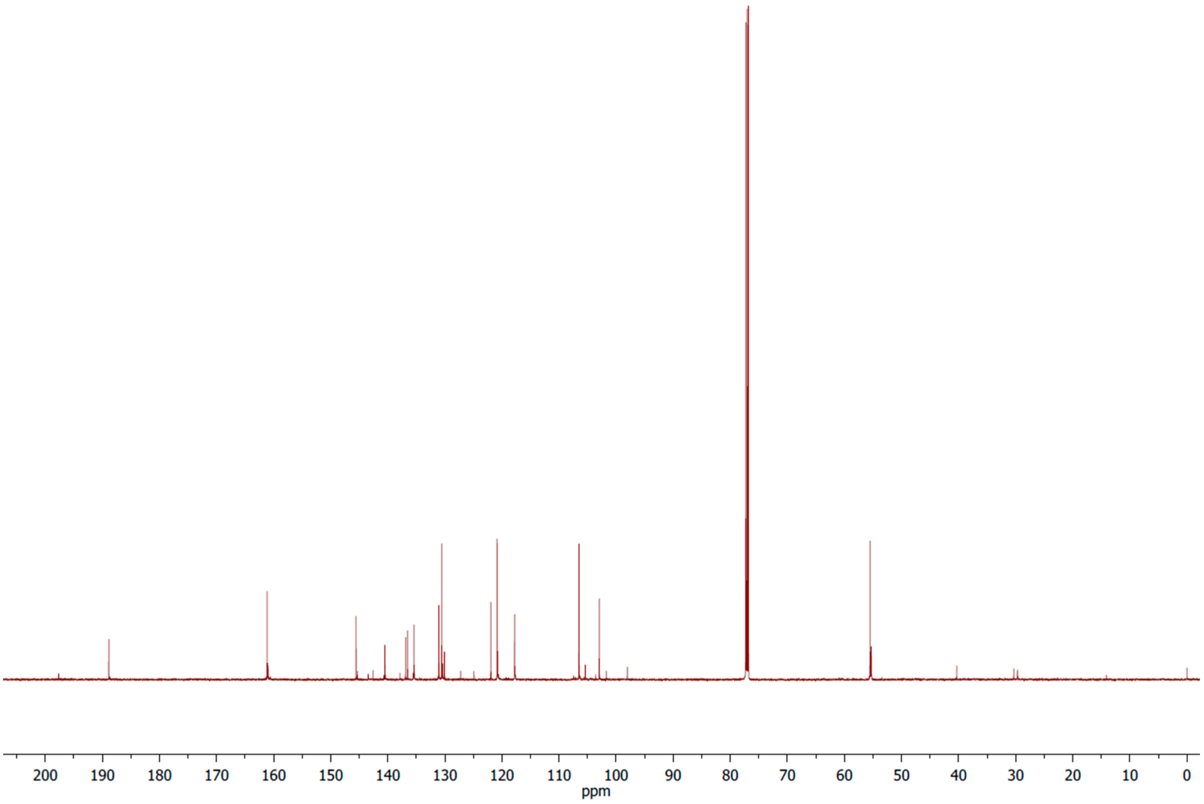

Figure S41. ^{13}C NMR (151 MHz) spectrum of the **IBC19** in $CDCl_3$-*d1*

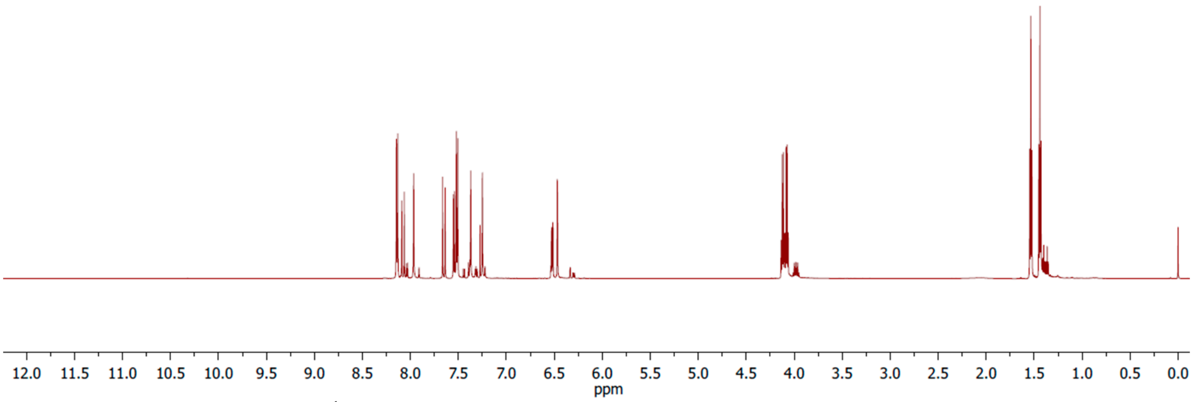

Figure S42. ^{1}H NMR (600 MHz) spectrum of the **IBC20** in $CDCl_3$-*d1*

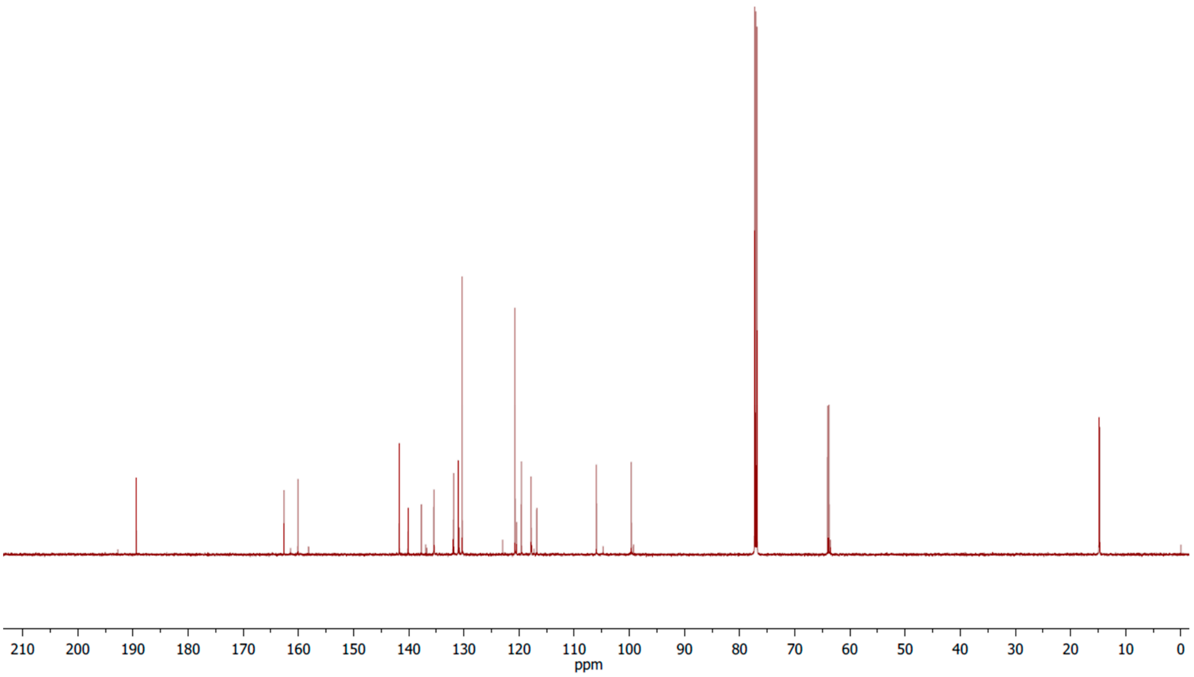

Figure S43. ^{13}C NMR (151 MHz) spectrum of the **IBC20** in $CDCl_3$-*d1*

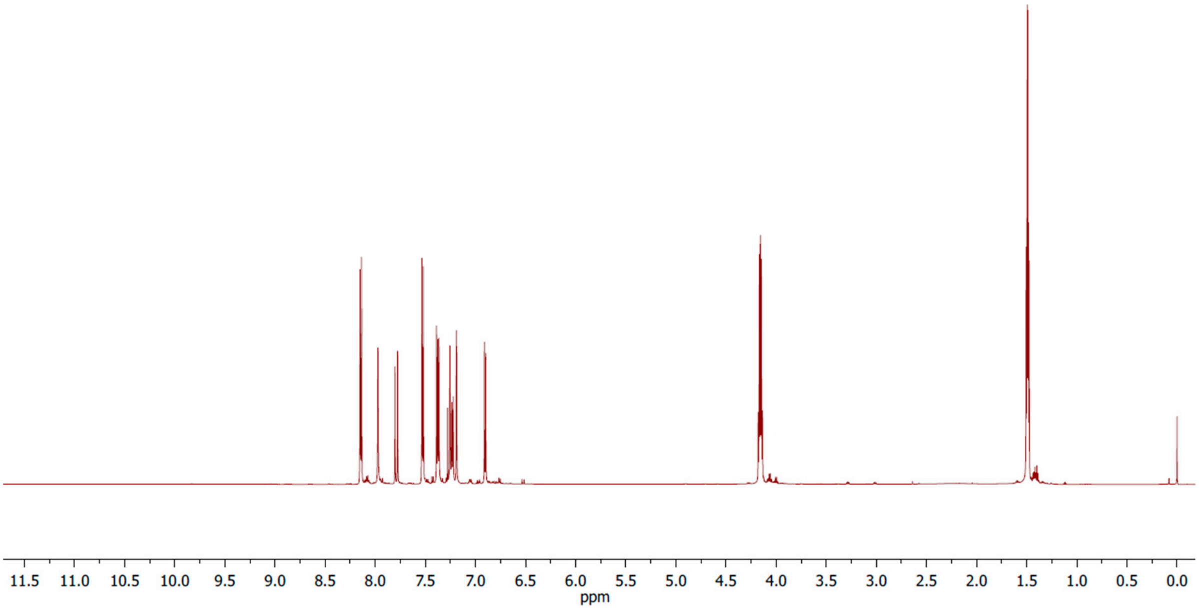

Figure S44. ^{1}H NMR (600 MHz) spectrum of the **IBC21** in $CDCl_3$-*d1*

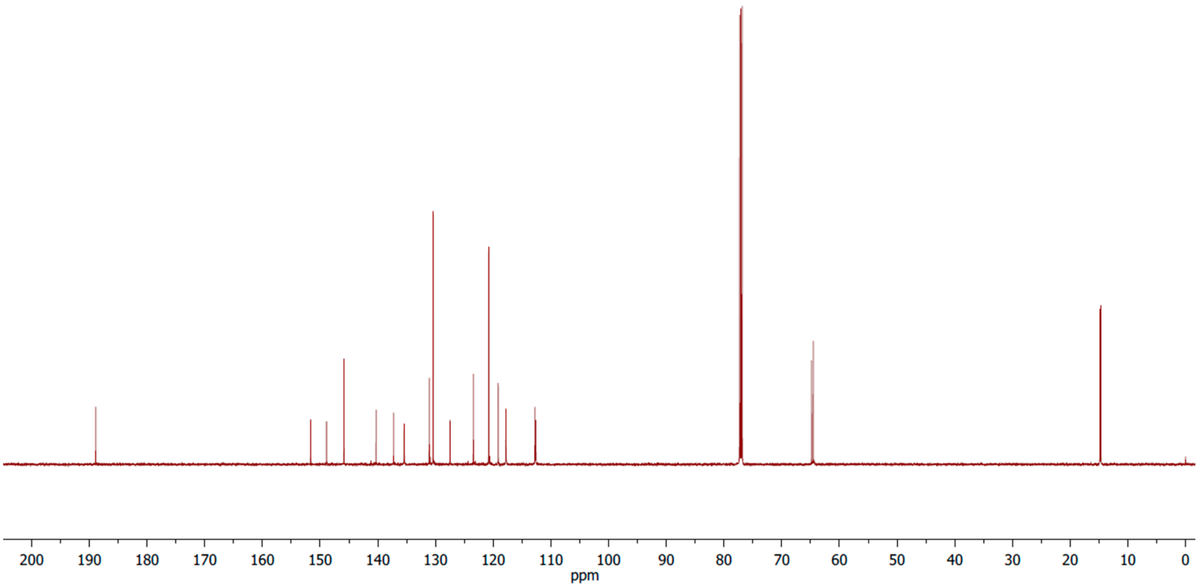

Figure S45. ^{13}C NMR (151 MHz) spectrum of the **IBC21** in $CDCl_3$-*d1*

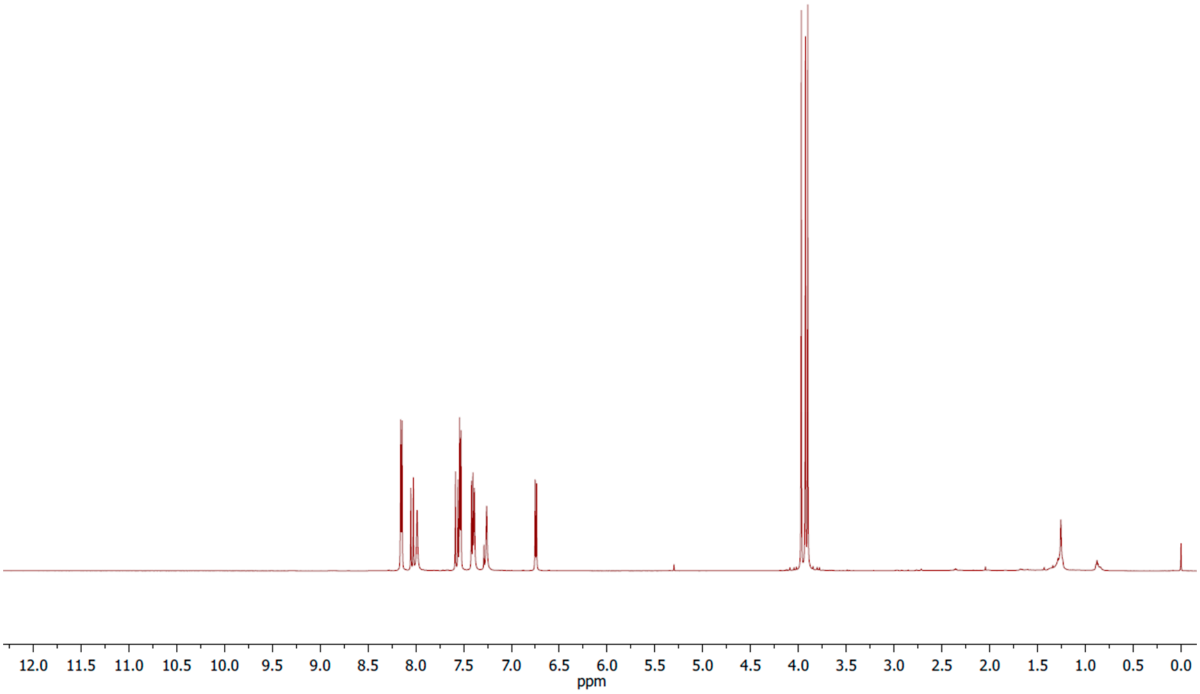

Figure S46. ^{1}H NMR (600 MHz) spectrum of the **IBC22** in $CDCl_3$-*d1*

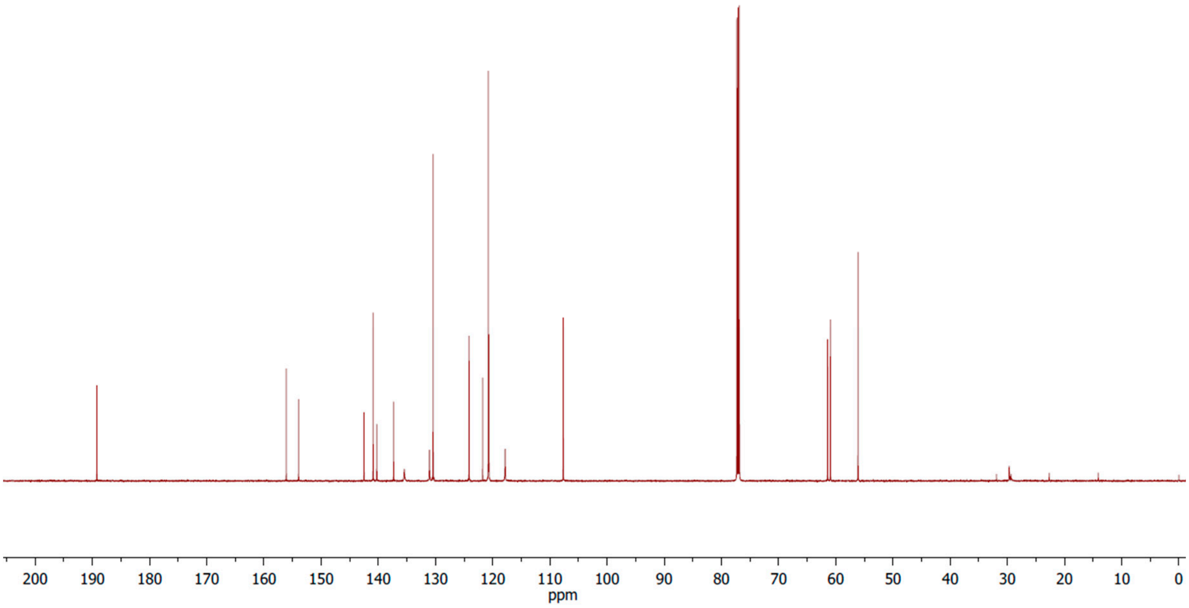

Figure S47. ^{13}C NMR (151 MHz) spectrum of the **IBC22** in $CDCl_3$-*d1*

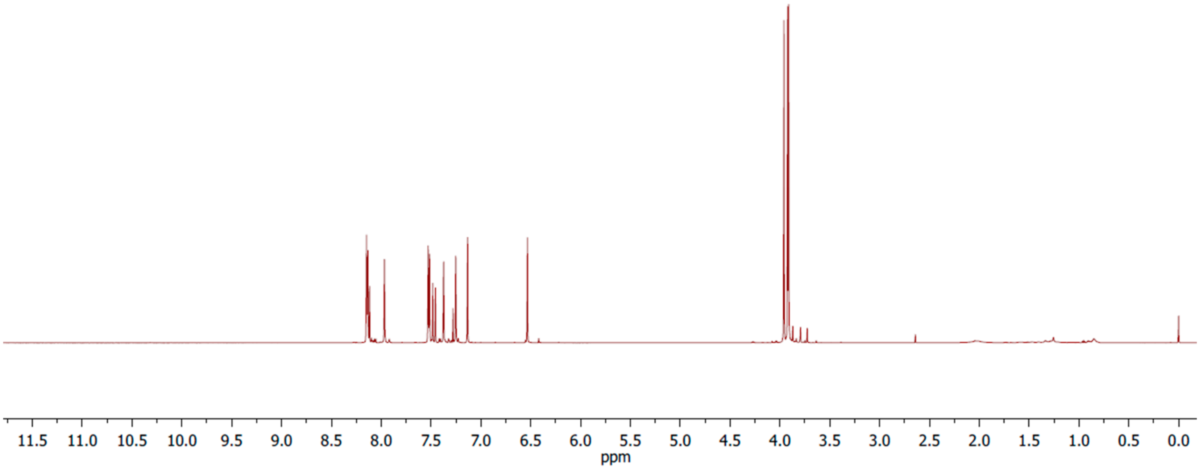

Figure S48. ^{1}H NMR (600 MHz) spectrum of the **IBC23** in $CDCl_3$-*d1*

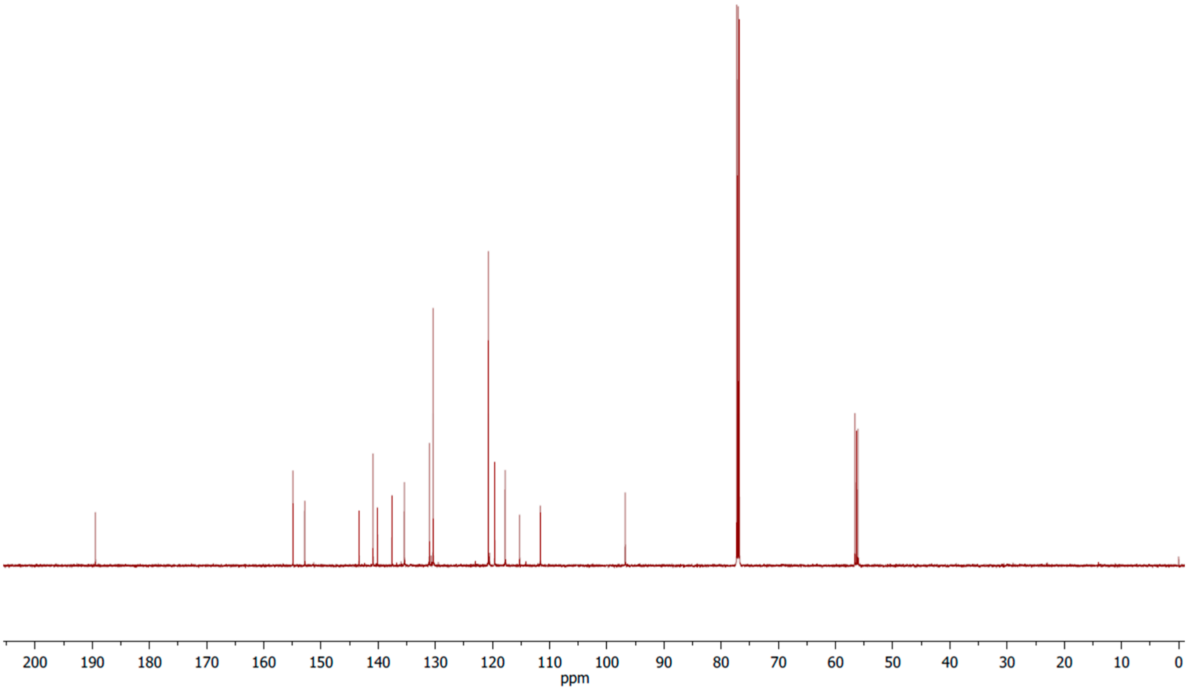

Figure S49. ^{13}C NMR (151 MHz) spectrum of the **IBC23** in $CDCl_3$-*d1*

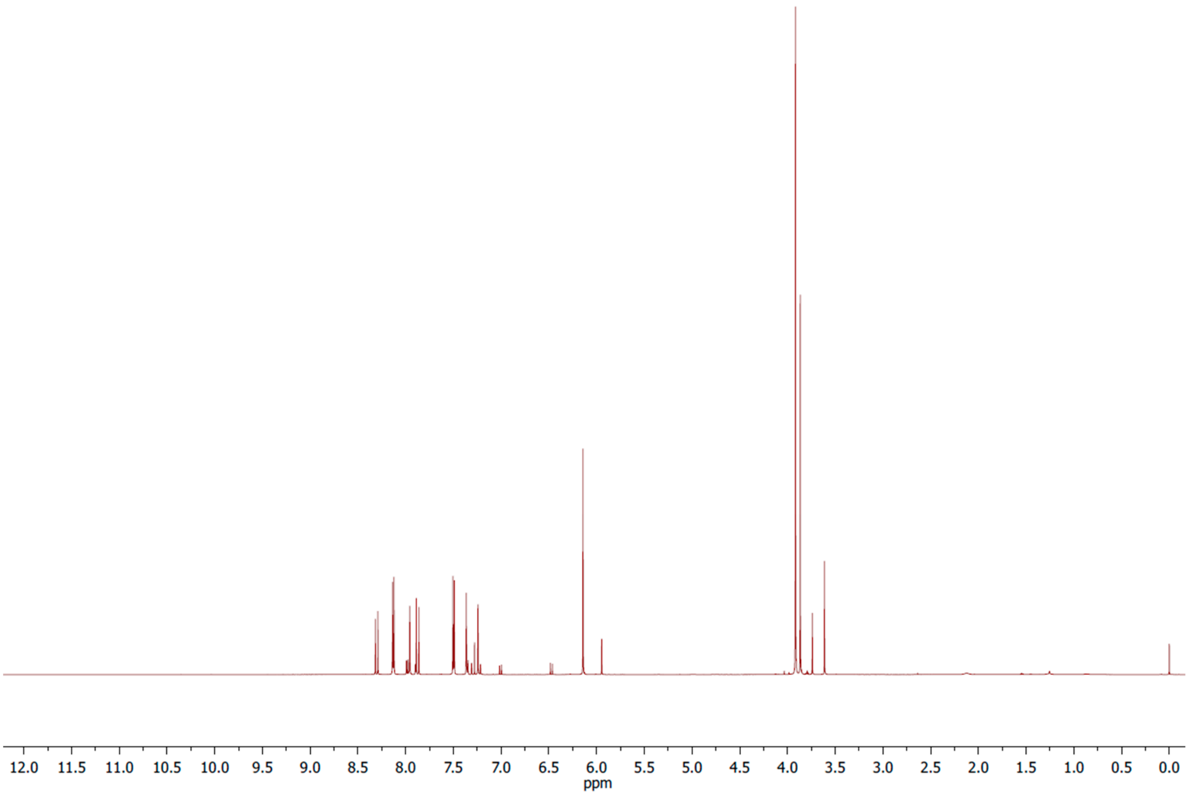

Figure S50. ^{1}H NMR (600 MHz) spectrum of the **IBC24** in $CDCl_3$-*d1*

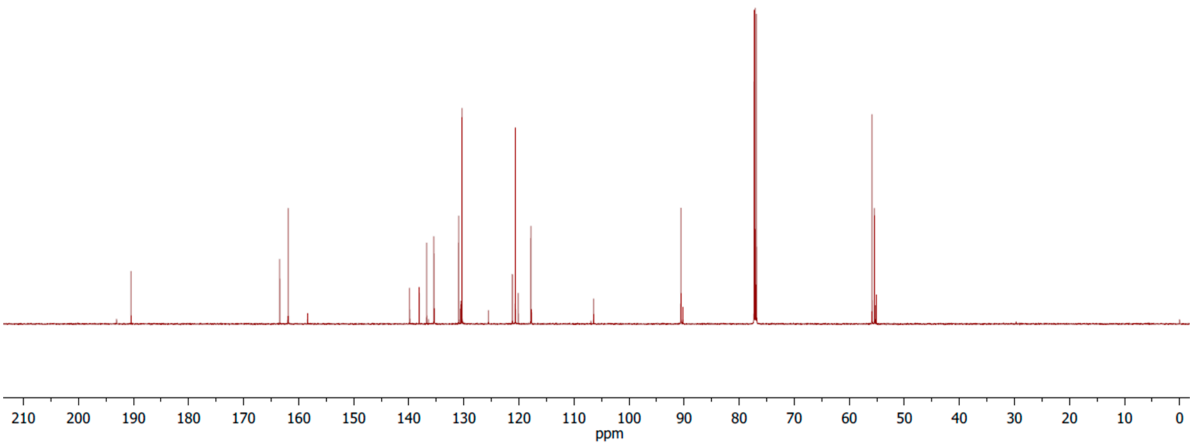

Figure S51. ^{13}C NMR (151 MHz) spectrum of the **IBC24** in $CDCl_3$-*d1*

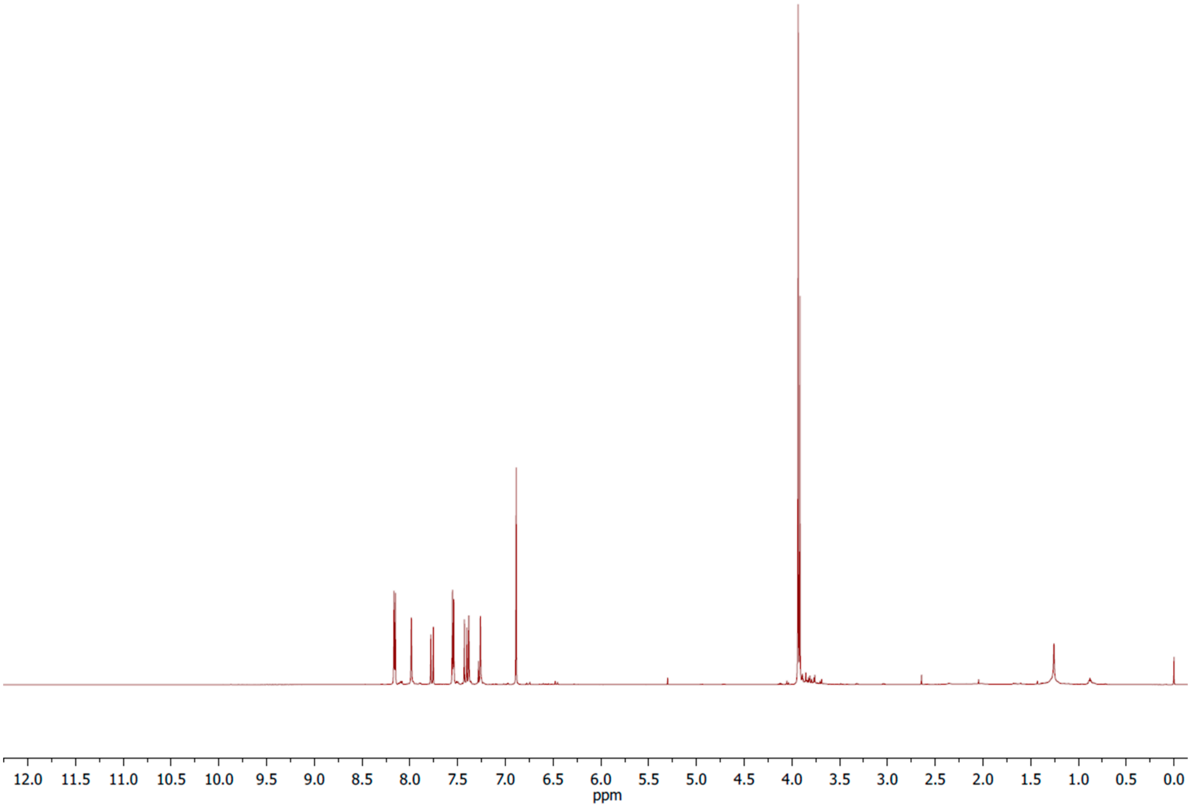

Figure S52. ^{1}H NMR (600 MHz) spectrum of the **IBC25** in $CDCl_3$-*d1*

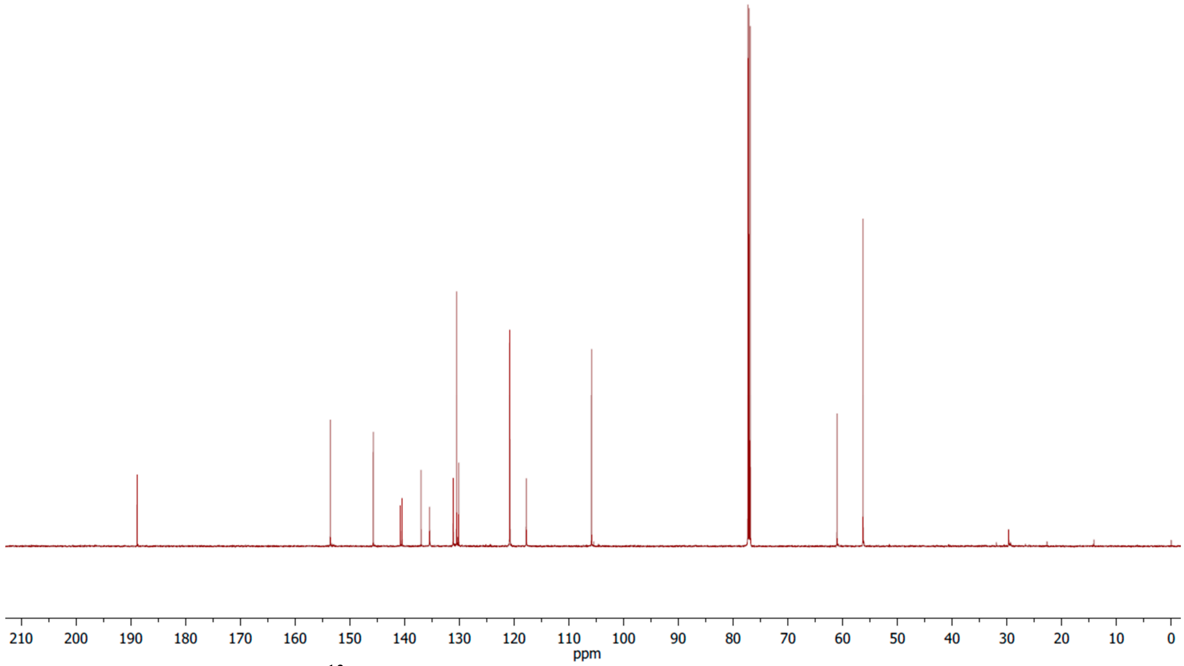

Figure S53. ^{13}C NMR (151 MHz) spectrum of the **IBC25** in $CDCl_3$-*d1*

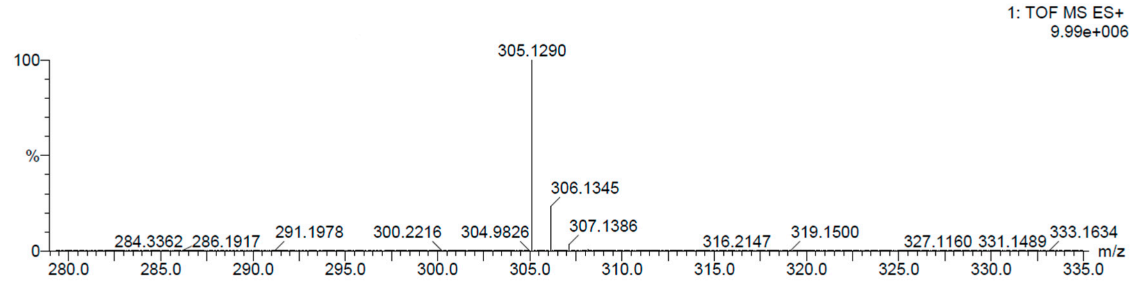

Minimum:				-5.5			
Maximum:		1000.0	10000.0	1000.0			

Mass	Calc. Mass	mDa	PPM	DBE	i-FIT	i-FIT (Norm)	Formula
305.1290	305.1290	0.0	0.0	12.5	2103.0	0.0	C19 H17 N2 O2

Figure S54. HRMS spectrum of the **IBC1**

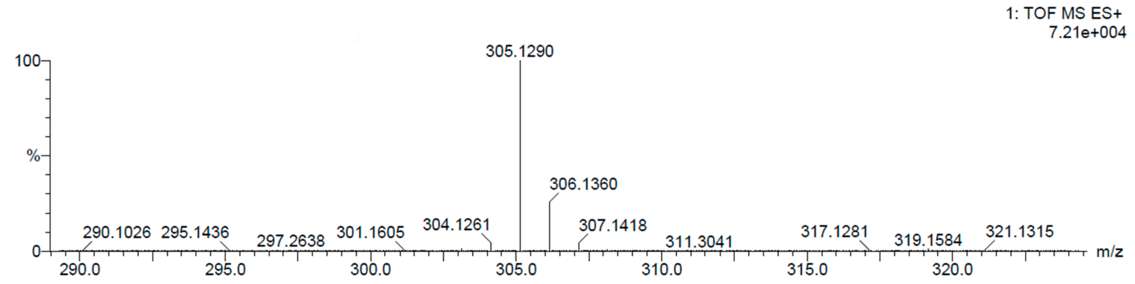

Minimum:				-5.5			
Maximum:		1000.0	10000.0	1000.0			

Mass	Calc. Mass	mDa	PPM	DBE	i-FIT	i-FIT (Norm)	Formula
305.1290	305.1290	0.0	0.0	12.5	1207.9	0.0	C19 H17 N2 O2

Figure S55. HRMS spectrum of the **IBC2**

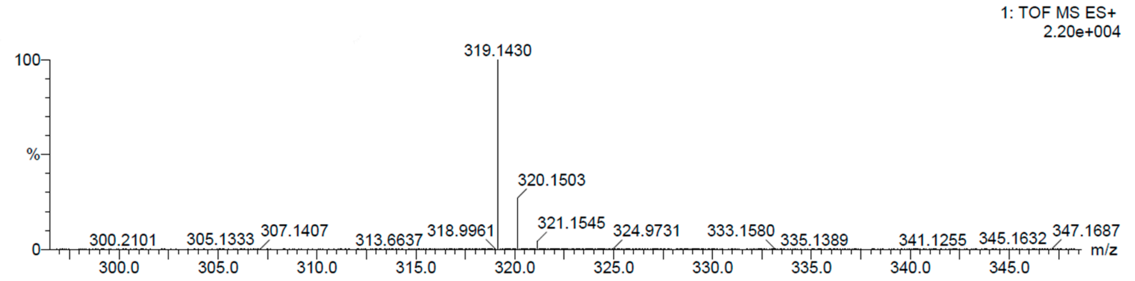

Minimum:				-5.5			
Maximum:		1000.0	10000.0	1000.0			

Mass	Calc. Mass	mDa	PPM	DBE	i-FIT	i-FIT (Norm)	Formula
319.1430	319.1447	-1.7	-5.3	12.5	800.5	0.0	C20 H19 N2 O2

Figure S56. HRMS spectrum of the **IBC3**

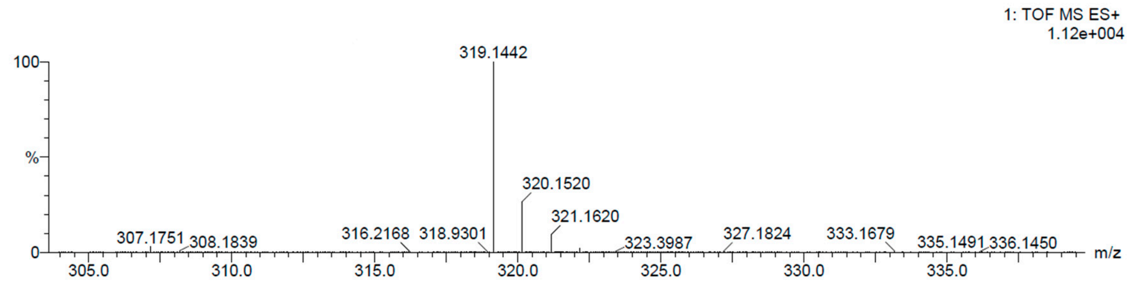

Minimum:				-5.5			
Maximum:		1000.0	10000.0	1000.0			
Mass	Calc. Mass	mDa	PPM	DBE	i-FIT	i-FIT (Norm)	Formula
319.1442	319.1447	-0.5	-1.6	12.5	627.5	0.0	C20 H19 N2 O2

Figure S57. HRMS spectrum of the **IBC4**

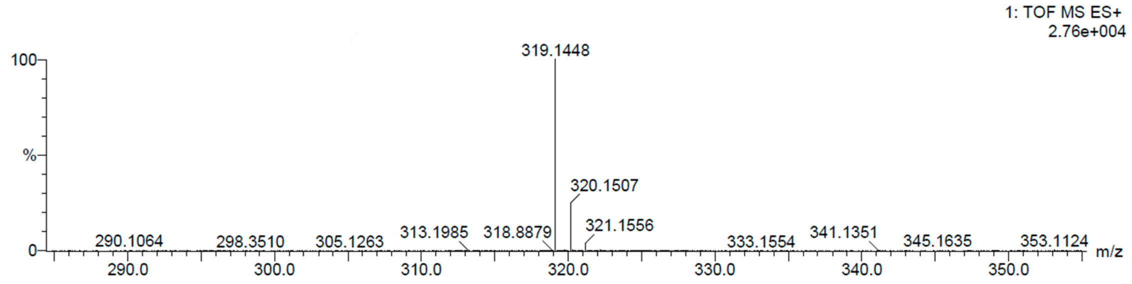

Minimum:				-5.5			
Maximum:		1000.0	10000.0	1000.0			
Mass	Calc. Mass	mDa	PPM	DBE	i-FIT	i-FIT (Norm)	Formula
319.1448	319.1447	0.1	0.3	12.5	747.2	0.0	C20 H19 N2 O2

Figure S58. HRMS spectrum of the **IBC5**

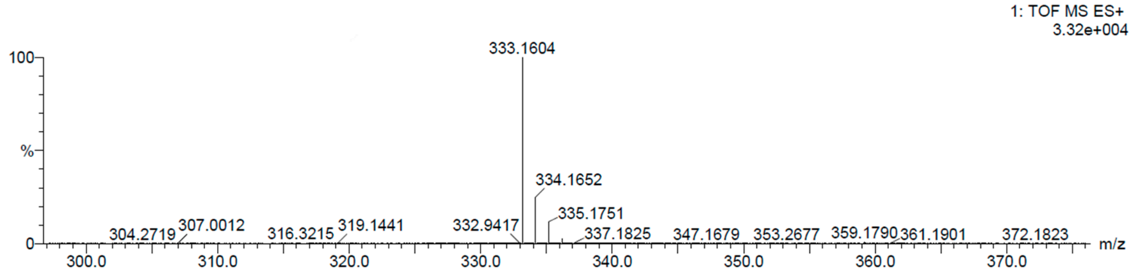

Minimum:				-5.5			
Maximum:		1000.0	10000.0	1000.0			
Mass	Calc. Mass	mDa	PPM	DBE	i-FIT	i-FIT (Norm)	Formula
333.1604	333.1603	0.1	0.3	12.5	949.6	0.0	C21 H21 N2 O2

Figure S59. HRMS spectrum of the **IBC6**

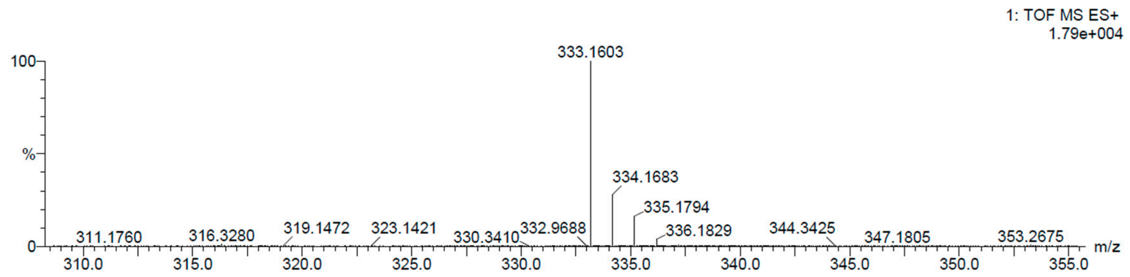

Mass	Calc. Mass	mDa	PPM	DBE	i-FIT	i-FIT (Norm)	Formula
Minimum:				-5.5			
Maximum:		1000.0	10000.0	1000.0			
333.1603	333.1603	0.0	0.0	12.5	833.7	0.0	C21 H21 N2 O2

Figure S60. HRMS spectrum of the **IBC7**

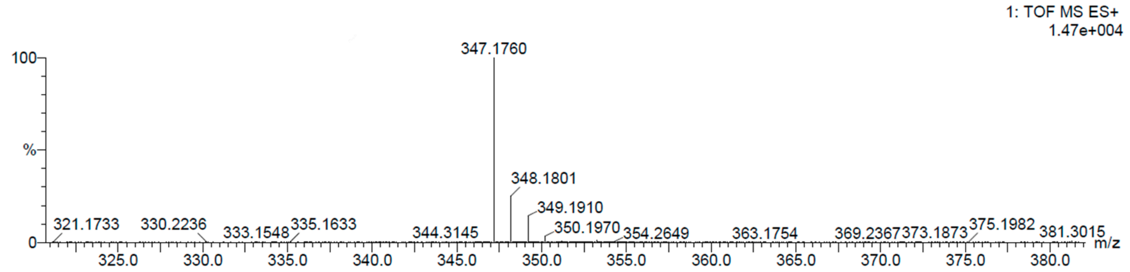

Mass	Calc. Mass	mDa	PPM	DBE	i-FIT	i-FIT (Norm)	Formula
Minimum:				-5.5			
Maximum:		1000.0	10000.0	1000.0			
347.1760	347.1760	0.0	0.0	12.5	761.7	0.0	C22 H23 N2 O2

Figure S61. HRMS spectrum of the **IBC8**

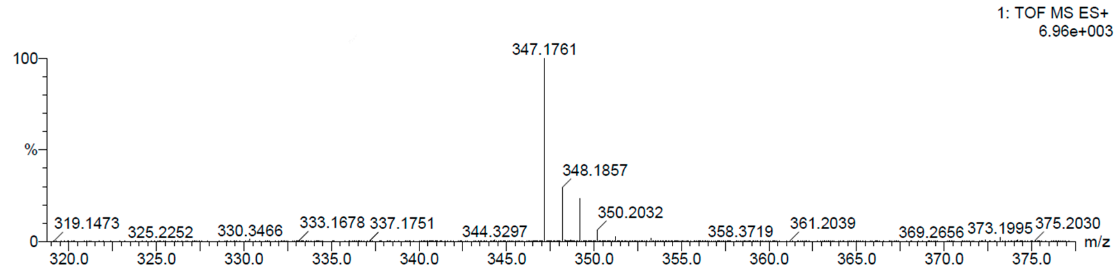

Mass	Calc. Mass	mDa	PPM	DBE	i-FIT	i-FIT (Norm)	Formula
Minimum:				-5.5			
Maximum:		1000.0	10000.0	1000.0			
347.1761	347.1760	0.1	0.3	12.5	562.3	0.0	C22 H23 N2 O2

Figure S62. HRMS spectrum of the **IBC9**

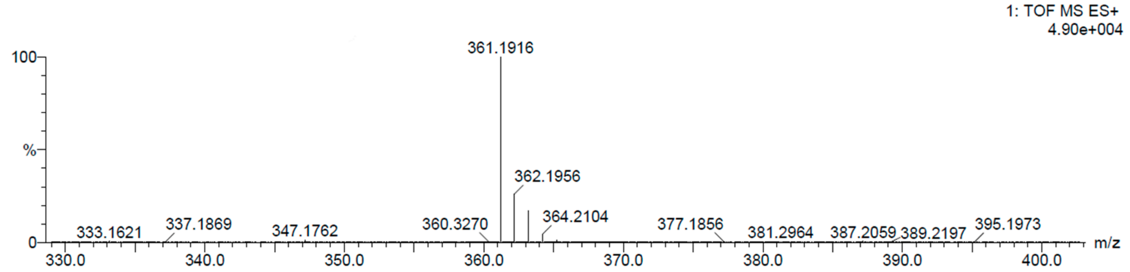

Mass	Calc. Mass	mDa	PPM	DBE	i-FIT	i-FIT (Norm)	Formula
Minimum:				-5.5			
Maximum:		1000.0	10000.0	1000.0			
361.1916	361.1916	0.0	0.0	12.5	1152.1	0.0	C23 H25 N2 O2

Figure S63. HRMS spectrum of the **IBC10**

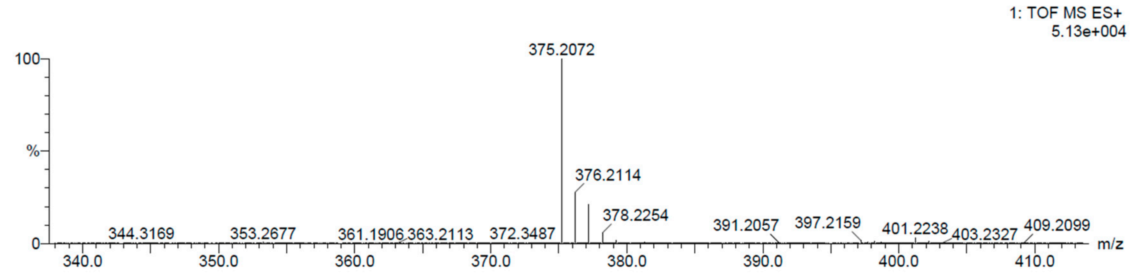

Minimum:				-5.5			
Maximum:		1000.0	10000.0	1000.0			
Mass	Calc. Mass	mDa	PPM	DBE	i-FIT	i-FIT (Norm)	Formula
375.2072	375.2073	-0.1	-0.3	12.5	1190.6	0.0	C24 H27 N2 O2

Figure S64. HRMS spectrum of the **IBC11**

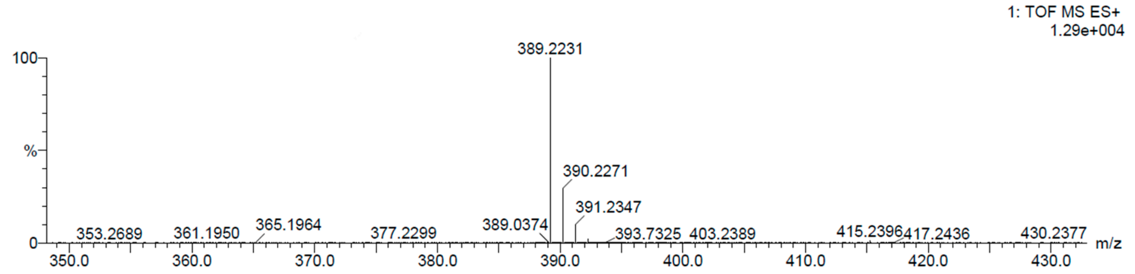

Minimum:				-5.5			
Maximum:		1000.0	10000.0	1000.0			
Mass	Calc. Mass	mDa	PPM	DBE	i-FIT	i-FIT (Norm)	Formula
389.2231	389.2229	0.2	0.5	12.5	665.6	0.0	C25 H29 N2 O2

Figure S65. HRMS spectrum of the **IBC12**

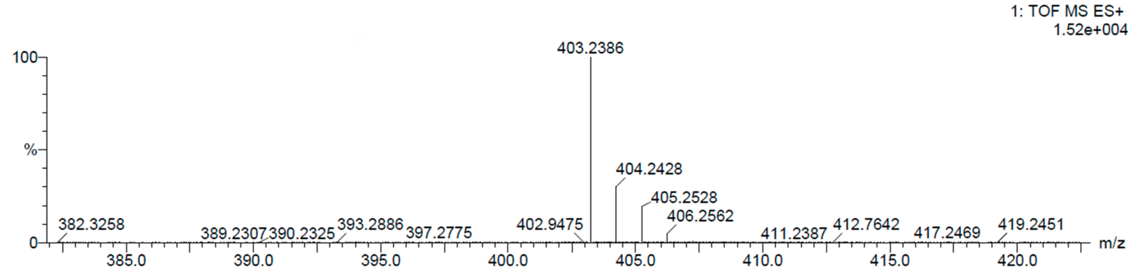

Minimum:				-5.5			
Maximum:		1000.0	10000.0	1000.0			
Mass	Calc. Mass	mDa	PPM	DBE	i-FIT	i-FIT (Norm)	Formula
403.2386	403.2386	0.0	0.0	12.5	780.7	0.0	C26 H31 N2 O2

Figure S66. HRMS spectrum of the **IBC13**

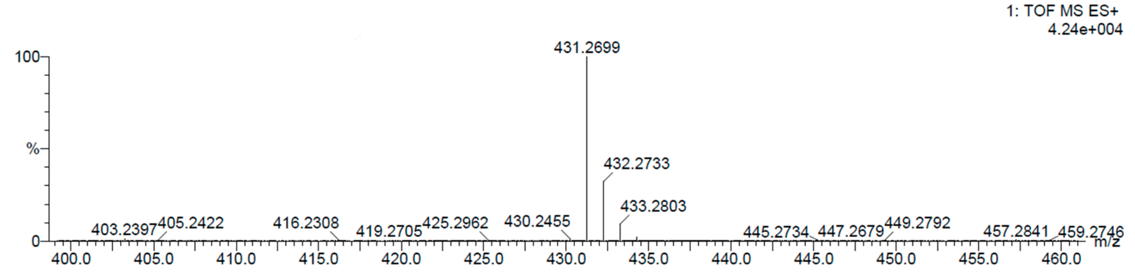

Minimum:				-5.5			
Maximum:		1000.0	10000.0	1000.0			
Mass	Calc. Mass	mDa	PPM	DBE	i-FIT	i-FIT (Norm)	Formula
431.2699	431.2699	0.0	0.0	12.5	1128.4	0.0	C28 H35 N2 O2

Figure S67. HRMS spectrum of the **IBC14**

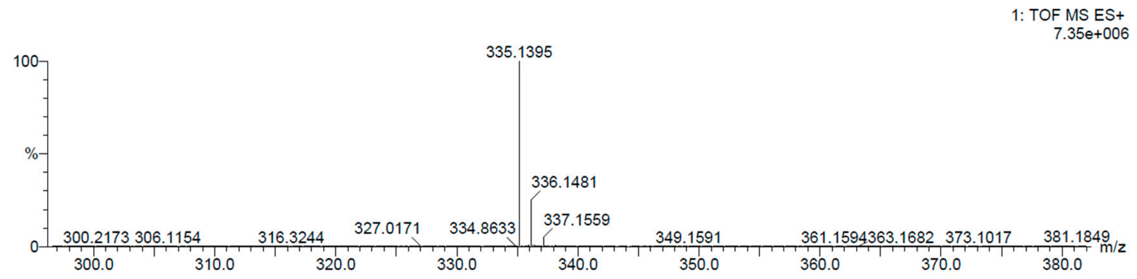

Minimum:				-5.5			
Maximum:		1000.0	1000.0	1000.0			
Mass	Calc. Mass	mDa	PPM	DBE	i-FIT	i-FIT (Norm)	Formula
335.1395	335.1396	-0.1	-0.3	12.5	1741.3	0.0	C20 H19 N2 O3

Figure S68. HRMS spectrum of the **IBC15**

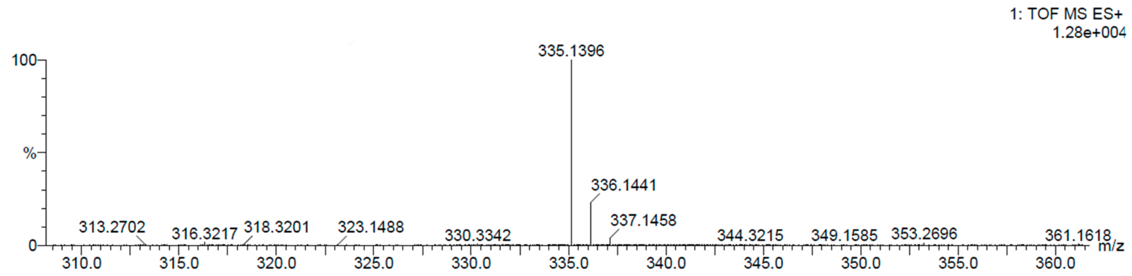

Minimum:				-5.5			
Maximum:		1000.0	10000.0	1000.0			
Mass	Calc. Mass	mDa	PPM	DBE	i-FIT	i-FIT (Norm)	Formula
335.1396	335.1396	0.0	0.0	12.5	601.3	0.0	C20 H19 N2 O3

Figure S69. HRMS spectrum of the **IBC16**

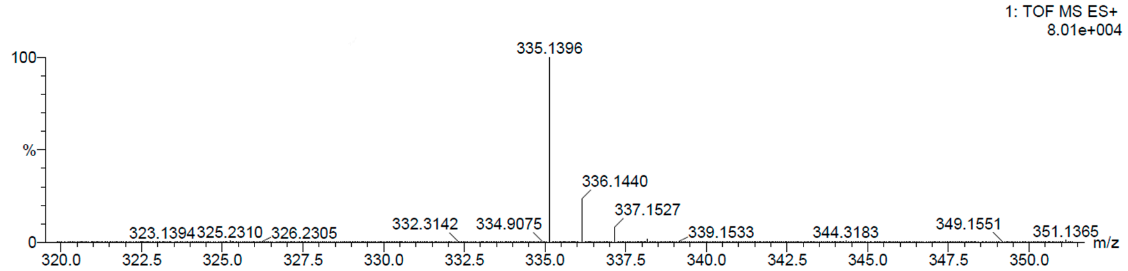

Minimum:				-5.5			
Maximum:		1000.0	10000.0	1000.0			
Mass	Calc. Mass	mDa	PPM	DBE	i-FIT	i-FIT (Norm)	Formula
335.1396	335.1396	0.0	0.0	12.5	1295.7	0.0	C20 H19 N2 O3

Figure S70. HRMS spectrum of the **IBC17**

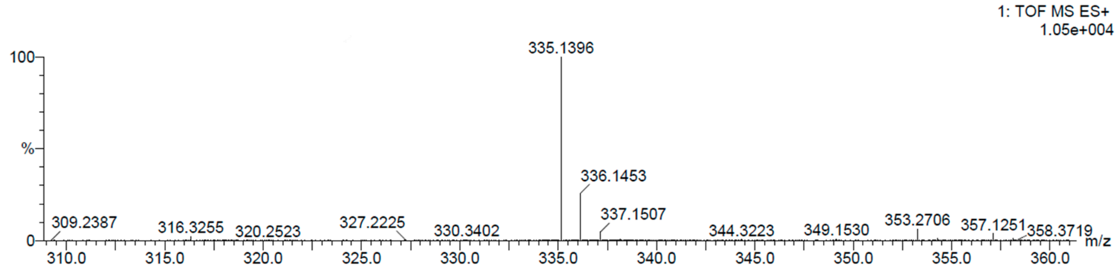

Minimum:				-5.5			
Maximum:		1000.0	1000.0	1000.0			
Mass	Calc. Mass	mDa	PPM	DBE	i-FIT	i-FIT (Norm)	Formula
335.1396	335.1396	0.0	0.0	12.5	616.7	0.0	C20 H19 N2 O3

Figure S71. HRMS spectrum of the **IBC18**

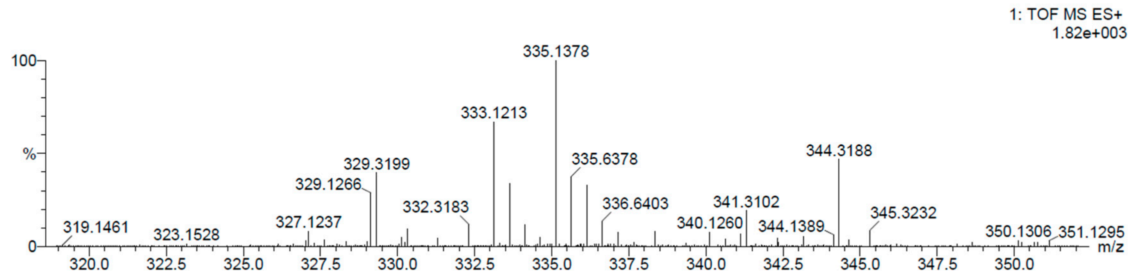

Minimum:				-5.5			
Maximum:		1000.0	1000.0	1000.0			
Mass	Calc. Mass	mDa	PPM	DBE	i-FIT	i-FIT (Norm)	Formula
335.1378	335.1396	-1.8	-5.4	12.5	502.2	0.0	C20 H19 N2 O3

Figure S72. HRMS spectrum of the **IBC19**

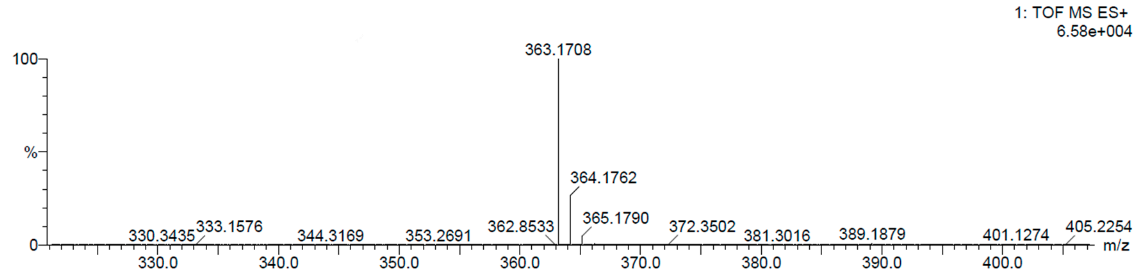

Minimum:				-5.5			
Maximum:		1000.0	10000.0	1000.0			
Mass	Calc. Mass	mDa	PPM	DBE	i-FIT	i-FIT (Norm)	Formula
363.1708	363.1709	-0.1	-0.3	12.5	1198.0	0.0	C22 H23 N2 O3

Figure S73. HRMS spectrum of the **IBC20**

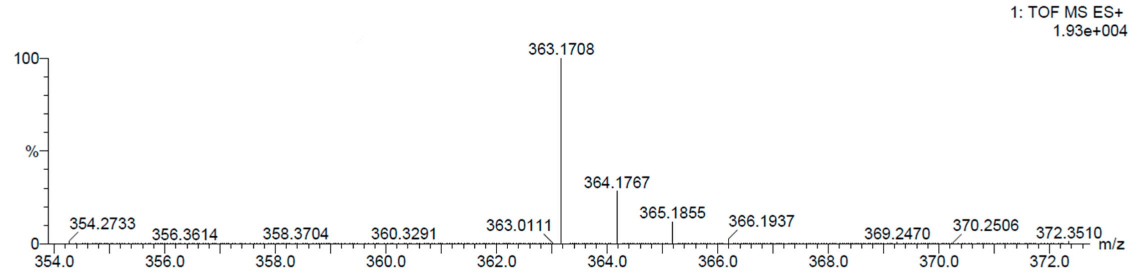

Minimum:				-5.5			
Maximum:		1000.0	10000.0	1000.0			
Mass	Calc. Mass	mDa	PPM	DBE	i-FIT	i-FIT (Norm)	Formula
363.1708	363.1709	-0.1	-0.3	12.5	838.0	0.0	C22 H23 N2 O3

Figure S74. HRMS spectrum of the **IBC21**

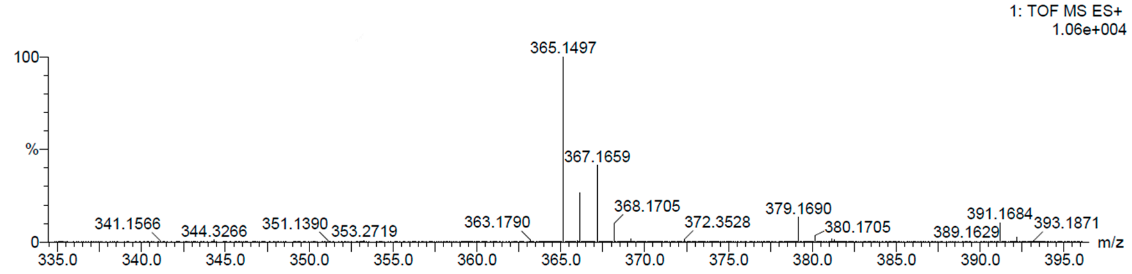

Minimum:				-5.5			
Maximum:		1000.0	10000.0	1000.0			
Mass	Calc. Mass	mDa	PPM	DBE	i-FIT	i-FIT (Norm)	Formula
365.1497	365.1501	-0.4	-1.1	12.5	818.7	0.0	C21 H21 N2 O4

Figure S75. HRMS spectrum of the **IBC22**

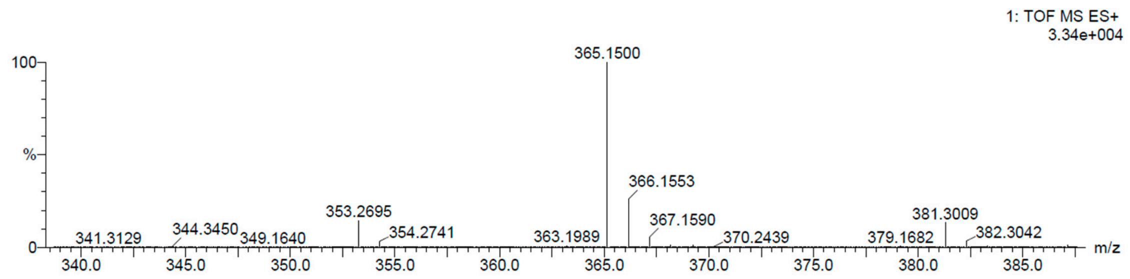

Minimum:				-5.5			
Maximum:		1000.0	1000.0	1000.0			
Mass	Calc. Mass	mDa	PPM	DBE	i-FIT	i-FIT (Norm)	Formula
365.1500	365.1501	-0.1	-0.3	12.5	1038.7	0.0	C21 H21 N2 O4

Figure S76. HRMS spectrum of the **IBC23**

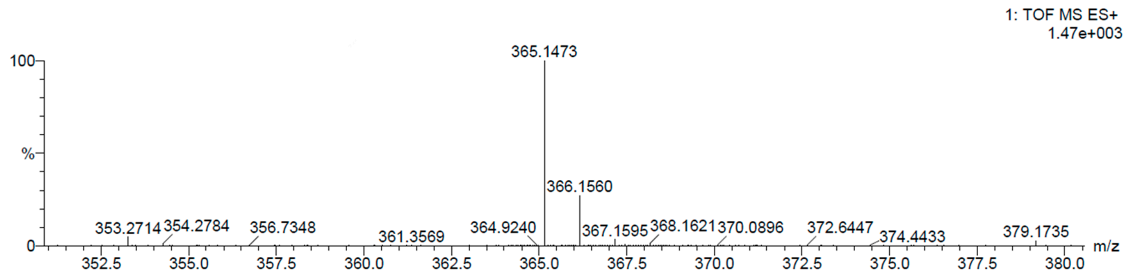

Minimum:				-5.5			
Maximum:		1000.0	1000.0	1000.0			
Mass	Calc. Mass	mDa	PPM	DBE	i-FIT	i-FIT (Norm)	Formula
365.1473	365.1501	-2.8	-7.7	12.5	177.5	0.0	C21 H21 N2 O4

Figure S77. HRMS spectrum of the **IBC24**

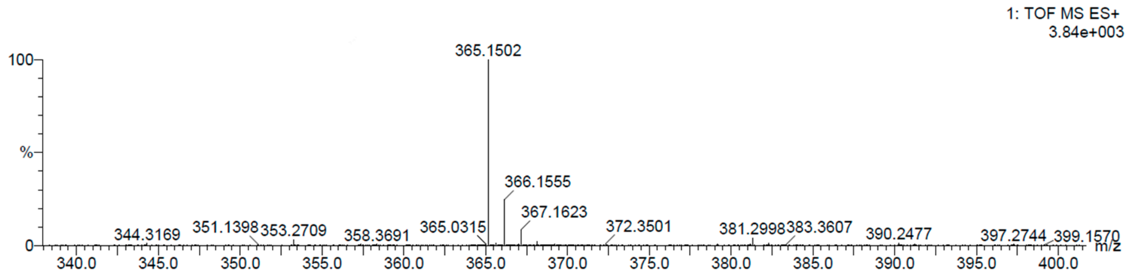

Minimum:				-5.5			
Maximum:		1000.0	1000.0	1000.0			
Mass	Calc. Mass	mDa	PPM	DBE	i-FIT	i-FIT (Norm)	Formula
365.1502	365.1501	0.1	0.3	12.5	425.5	0.0	C21 H21 N2 O4

Figure S78. HRMS spectrum of the **IBC25**

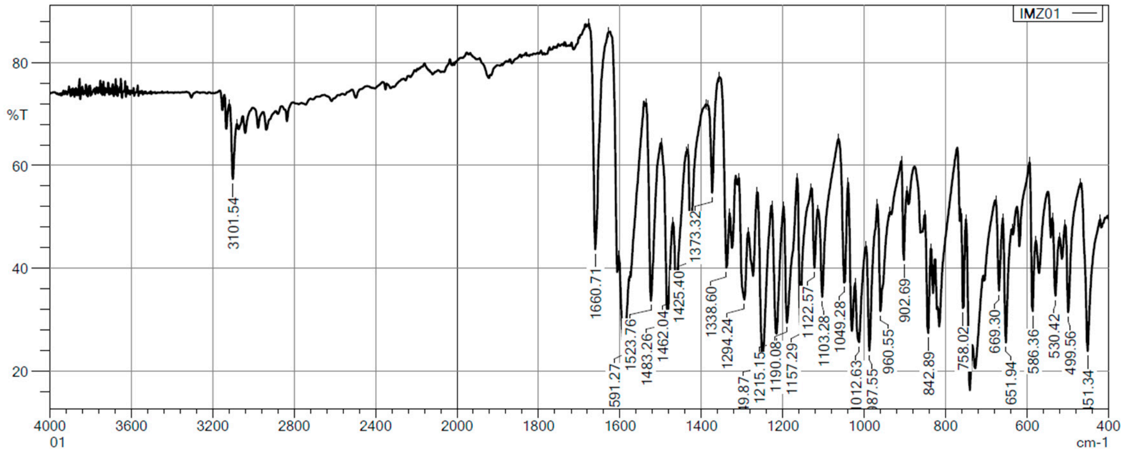

Figure S79. FTIR spectrum of the **IBC1**

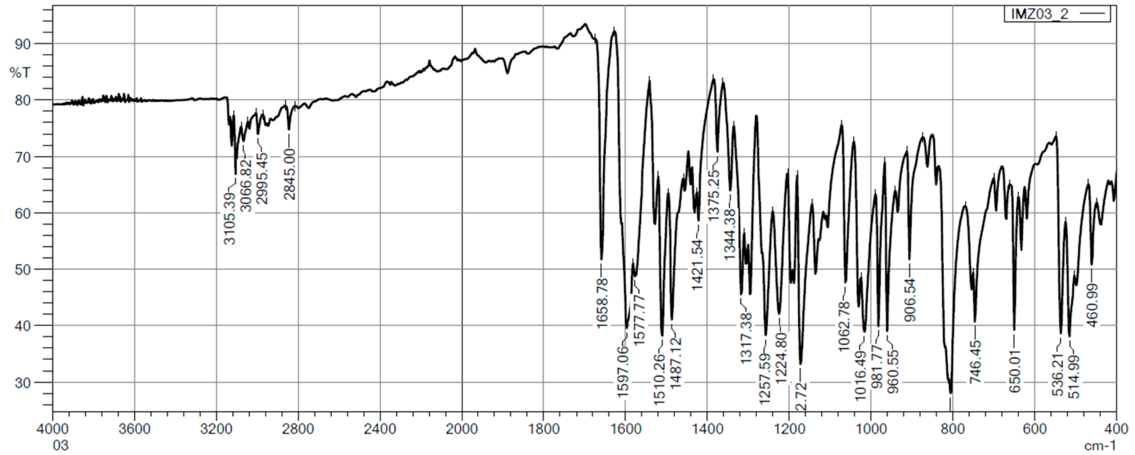

Figure S80. FTIR spectrum of the **IBC2**

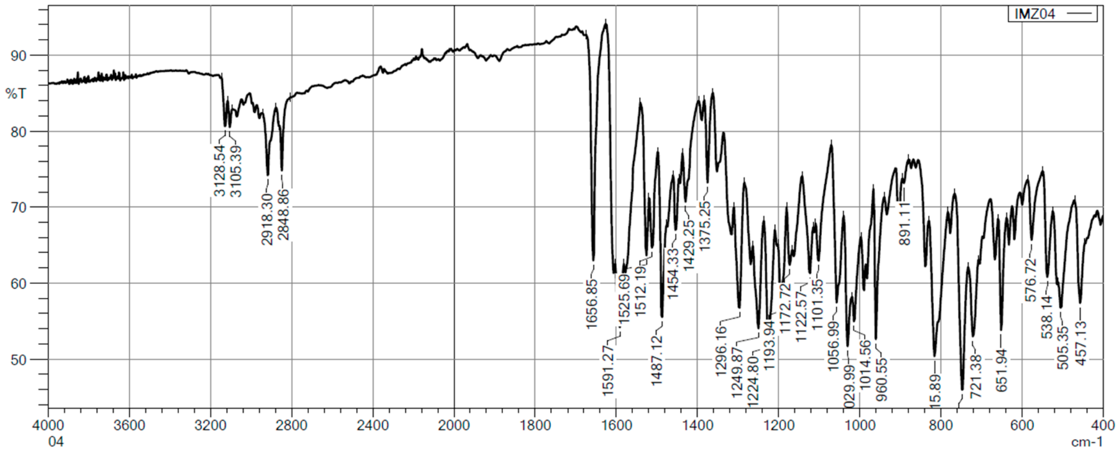

Figure S81. FTIR spectrum of the **IBC3**

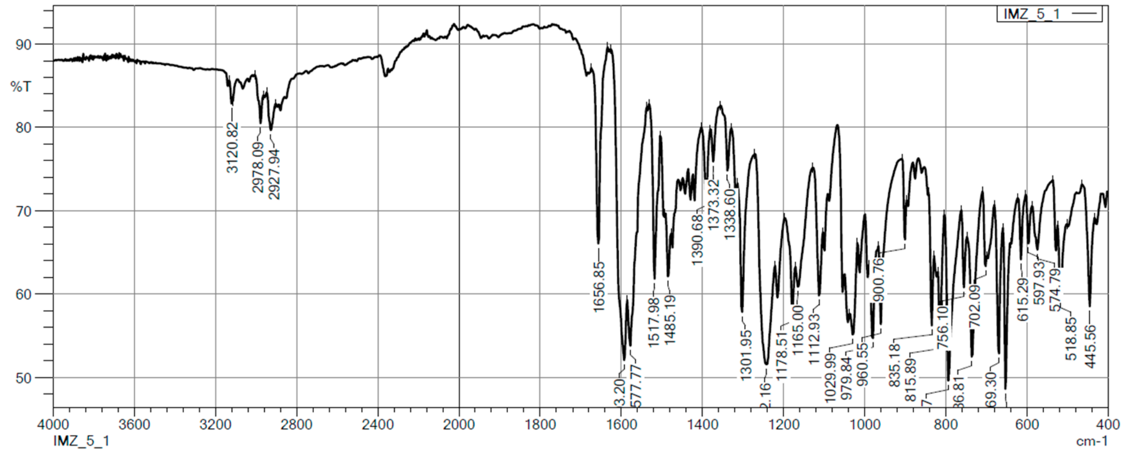

Figure S82. FTIR spectrum of the **IBC4**

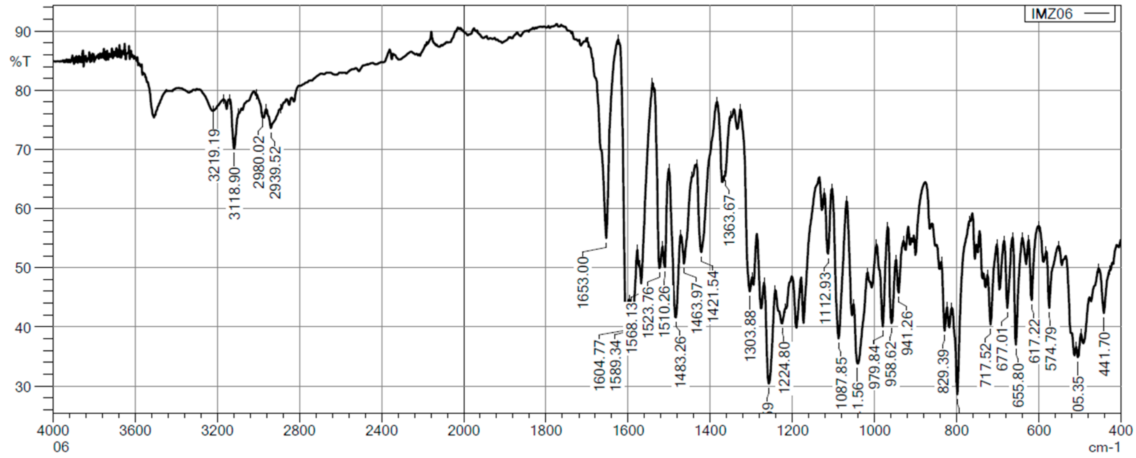

Figure S83. FTIR spectrum of the **IBC5**

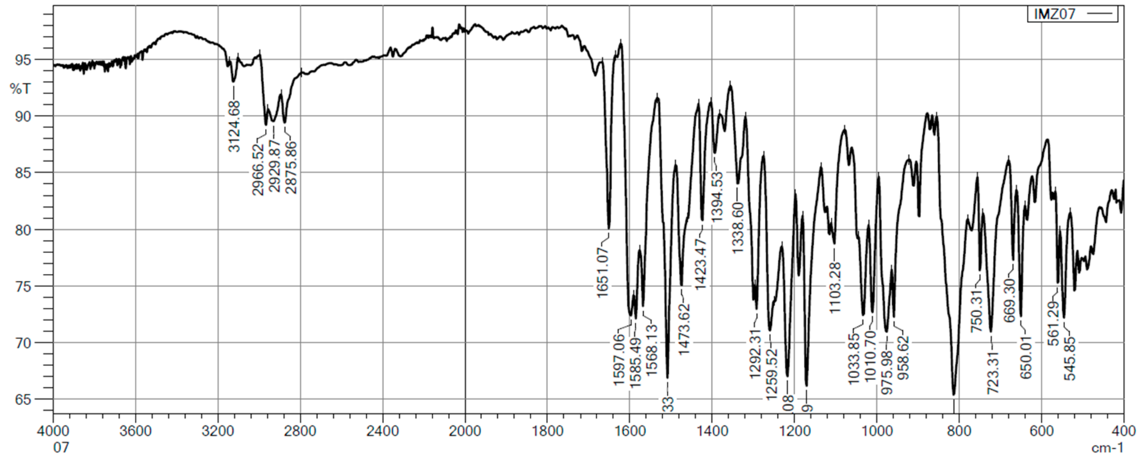

Figure S84. FTIR spectrum of the **IBC6**

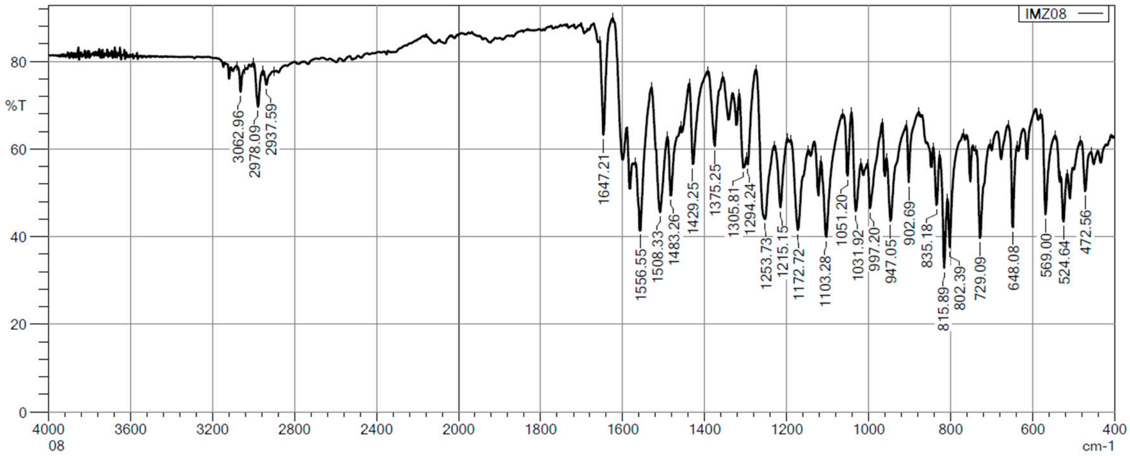

Figure S85. FTIR spectrum of the **IBC7**

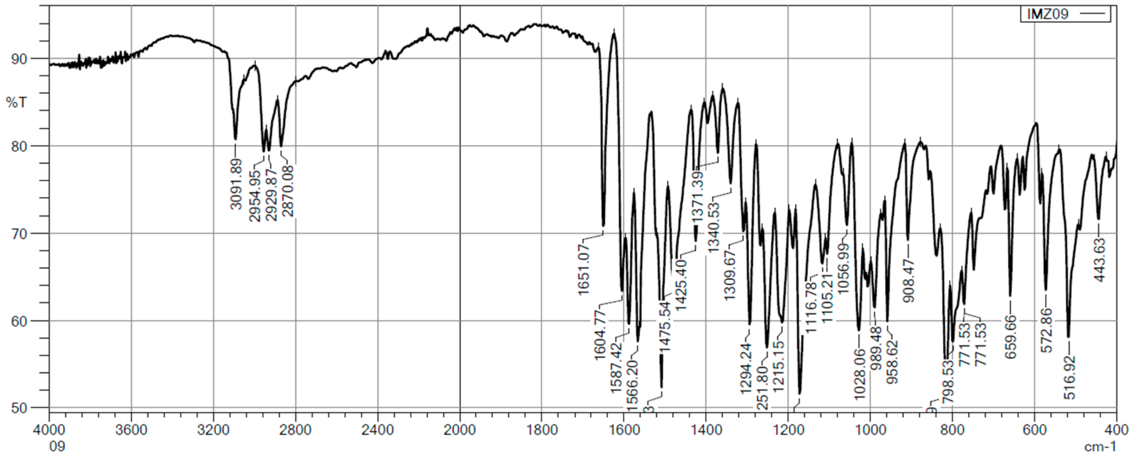

Figure S86. FTIR spectrum of the **IBC8**

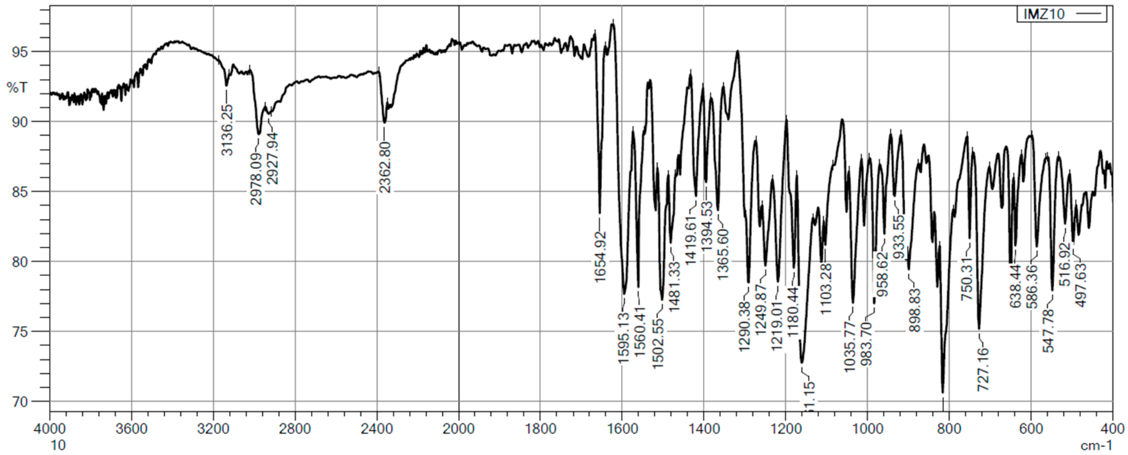

Figure S87. FTIR spectrum of the **IBC9**

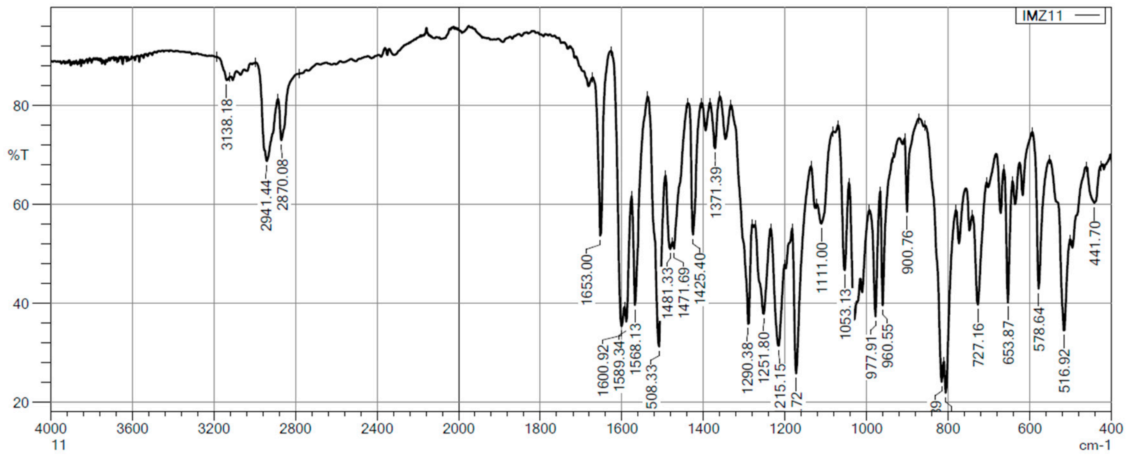

Figure S88. FTIR spectrum of the **IBC10**

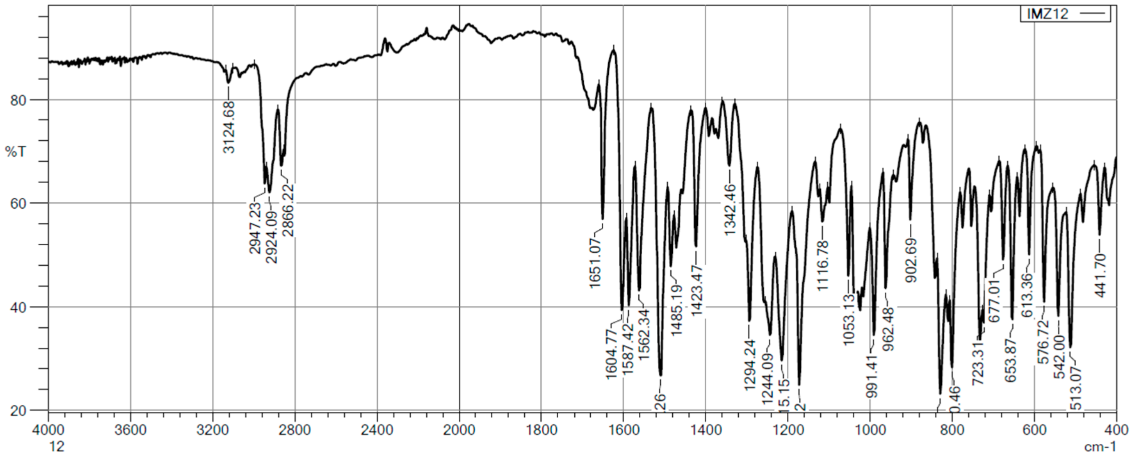

Figure S89. FTIR spectrum of the **IBC11**

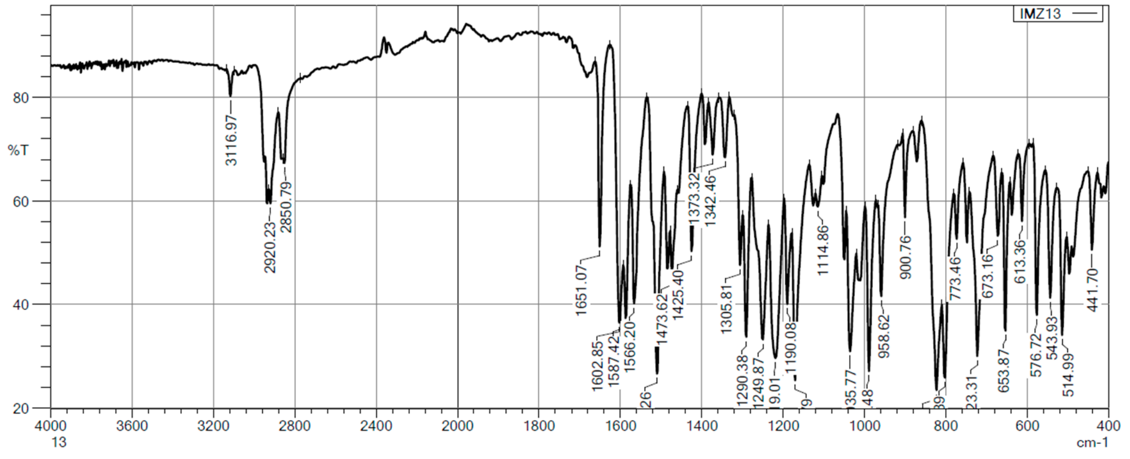

Figure S90. FTIR spectrum of the **IBC12**

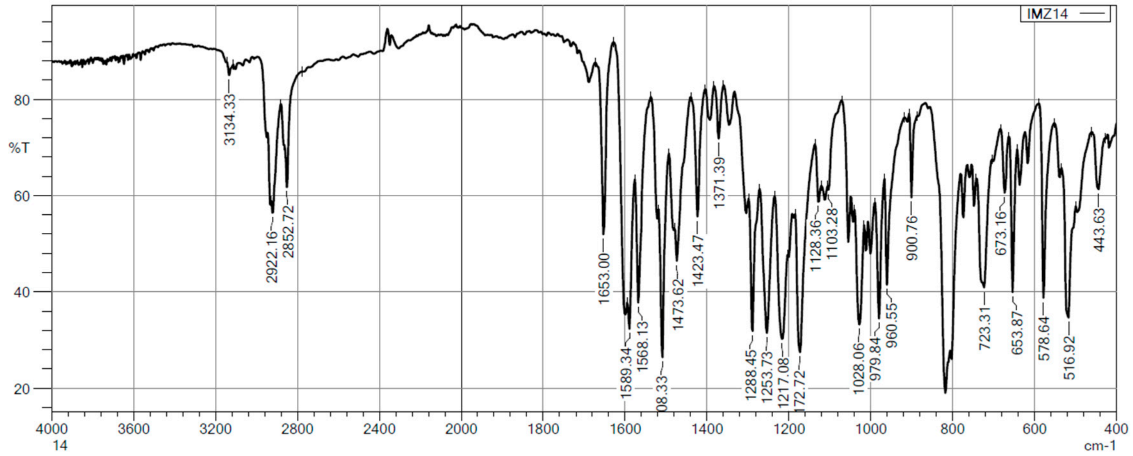

Figure S91. FTIR spectrum of the **IBC13**

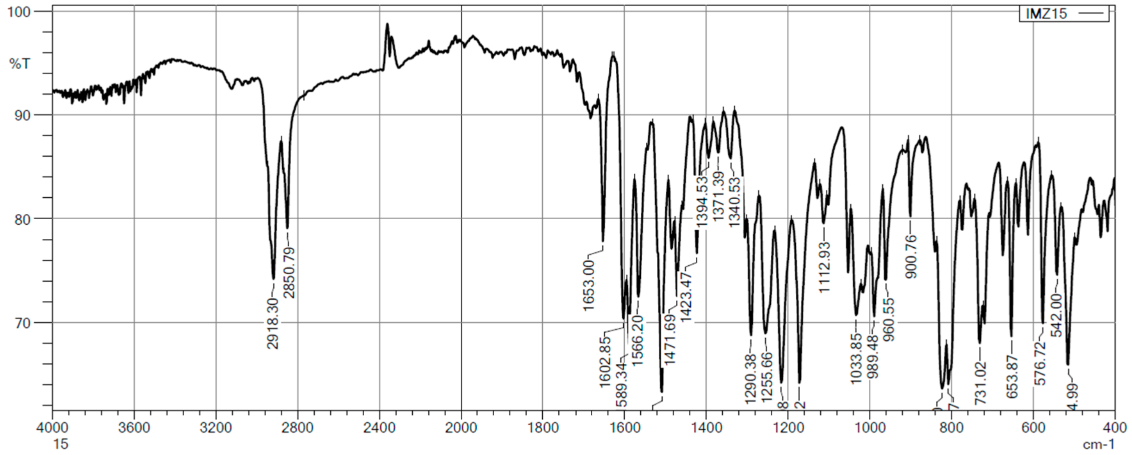

Figure S92. FTIR spectrum of the **IBC14**

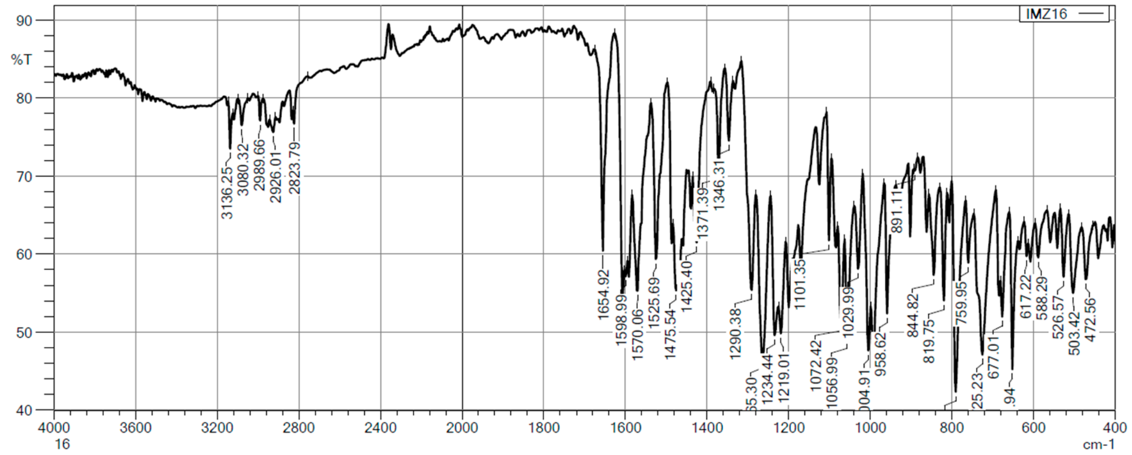

Figure S93. FTIR spectrum of the **IBC15**

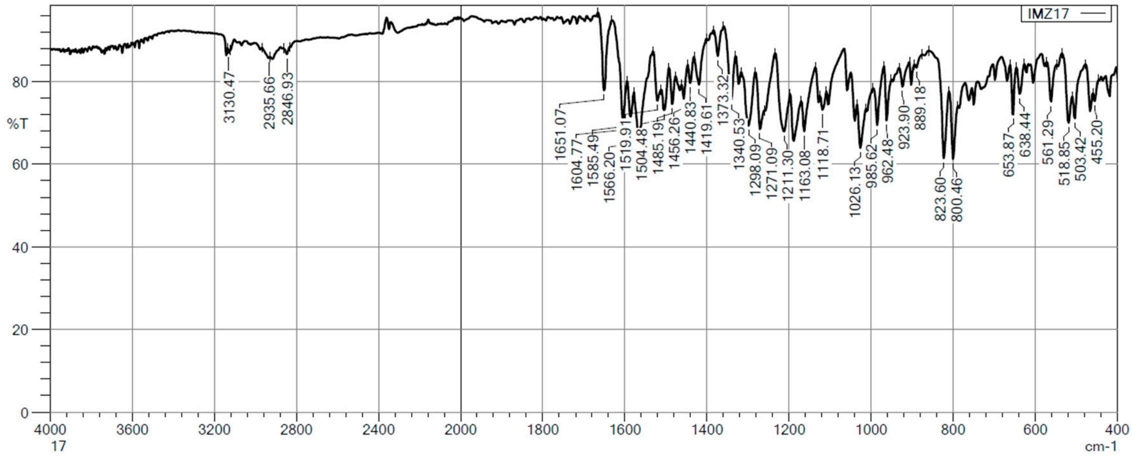

Figure S94. FTIR spectrum of the **IBC16**

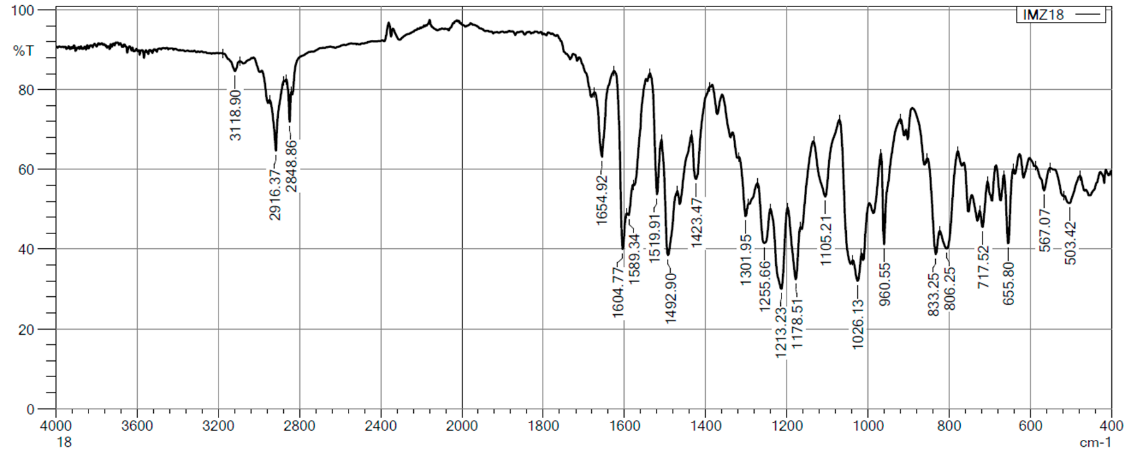

Figure S95. FTIR spectrum of the **IBC17**

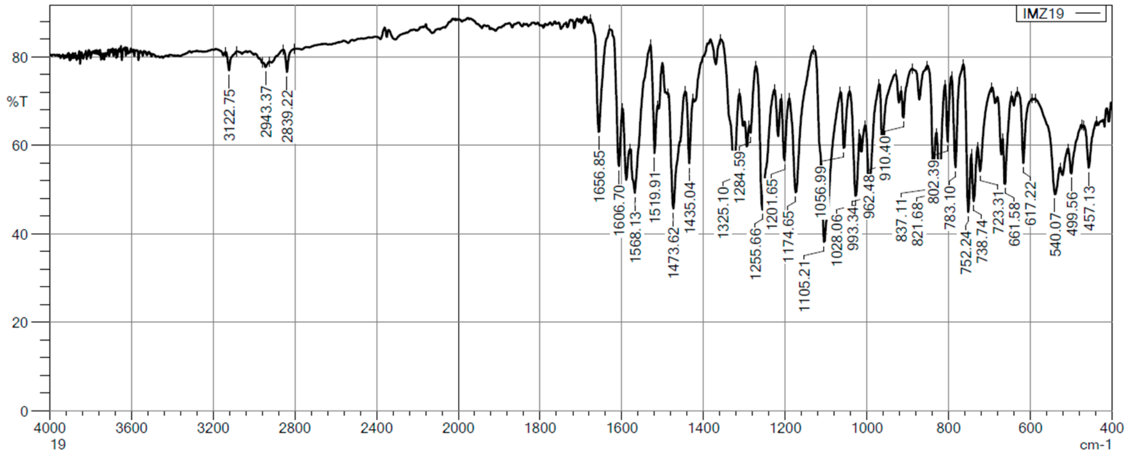

Figure S96. FTIR spectrum of the **IBC18**

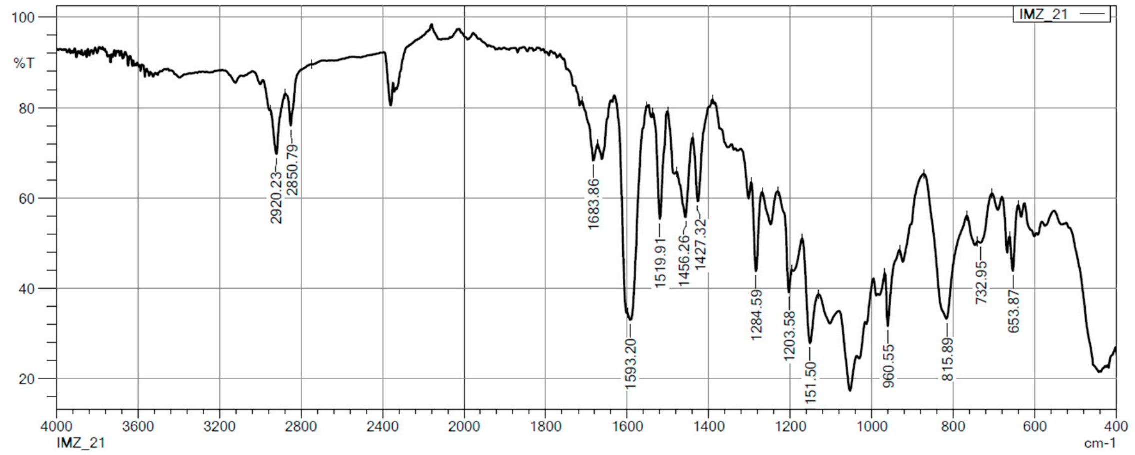

Figure S97. FTIR spectrum of the **IBC19**

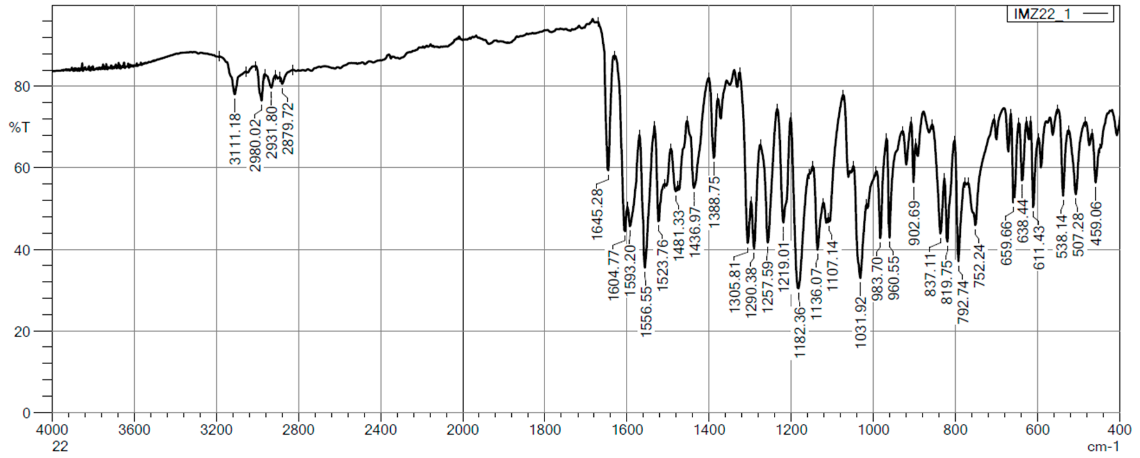

Figure S98. FTIR spectrum of the **IBC20**

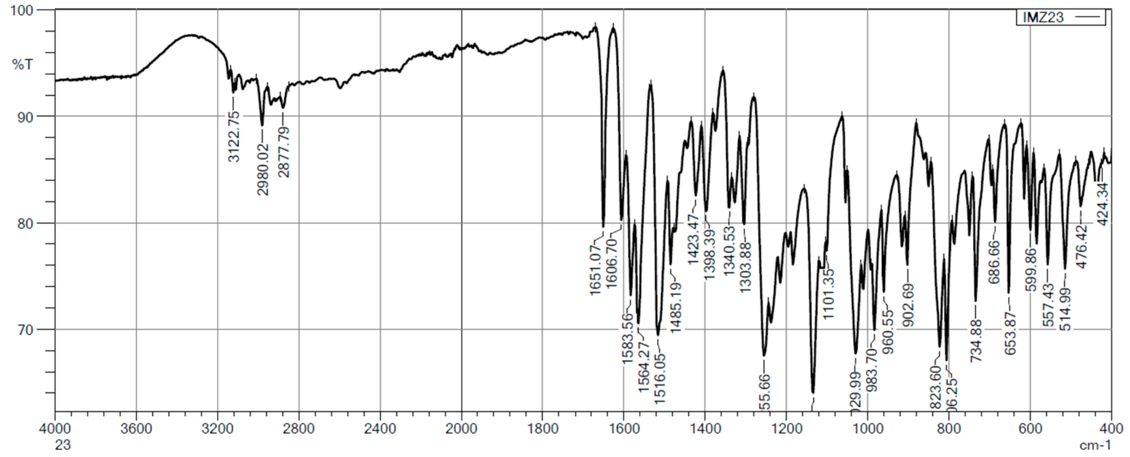

Figure S99. FTIR spectrum of the **IBC21**

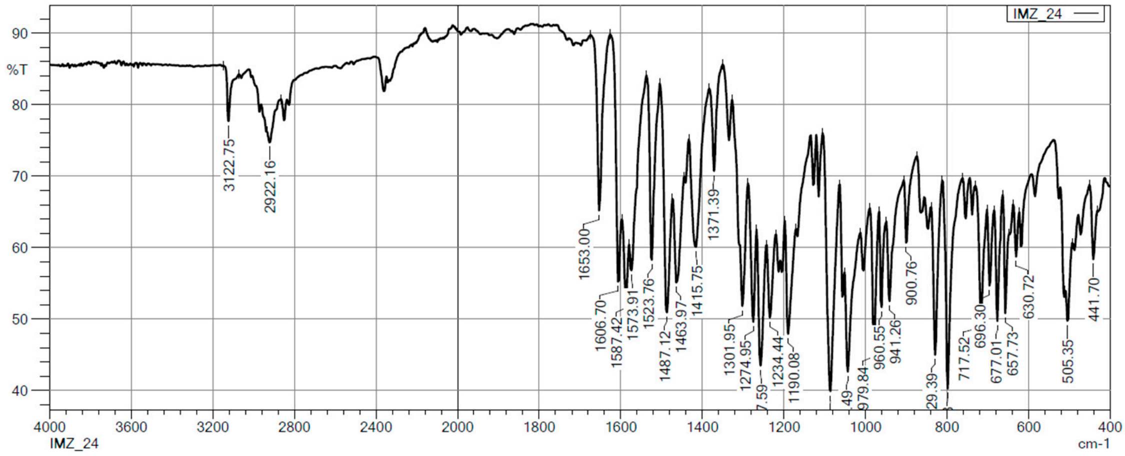

Figure S100. FTIR spectrum of the **IBC22**

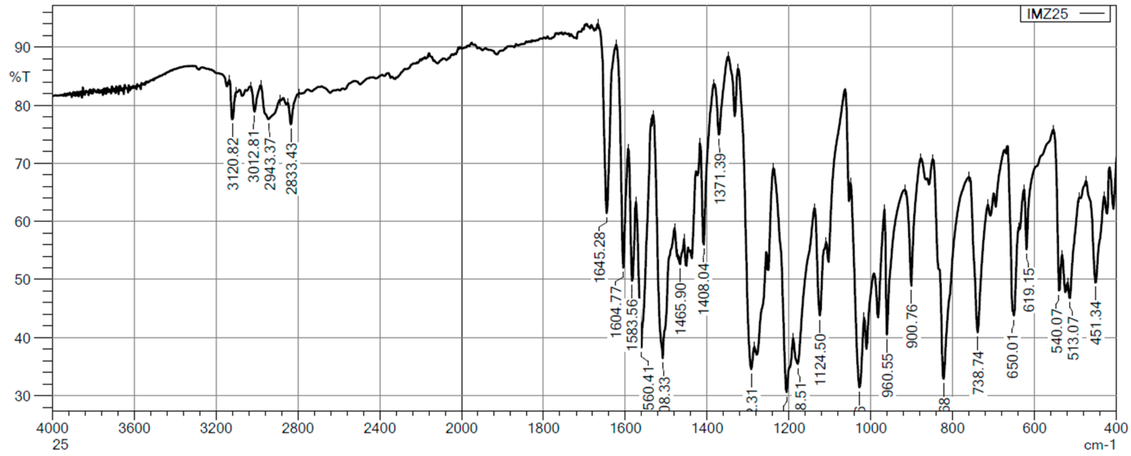

Figure S101. FTIR spectrum of the **IBC23**

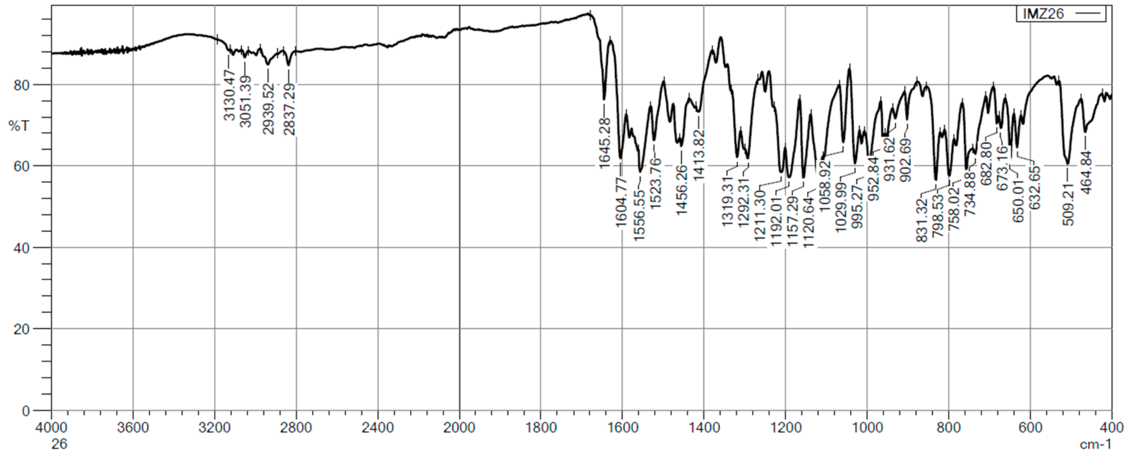

Figure S102. FTIR spectrum of the **IBC24**

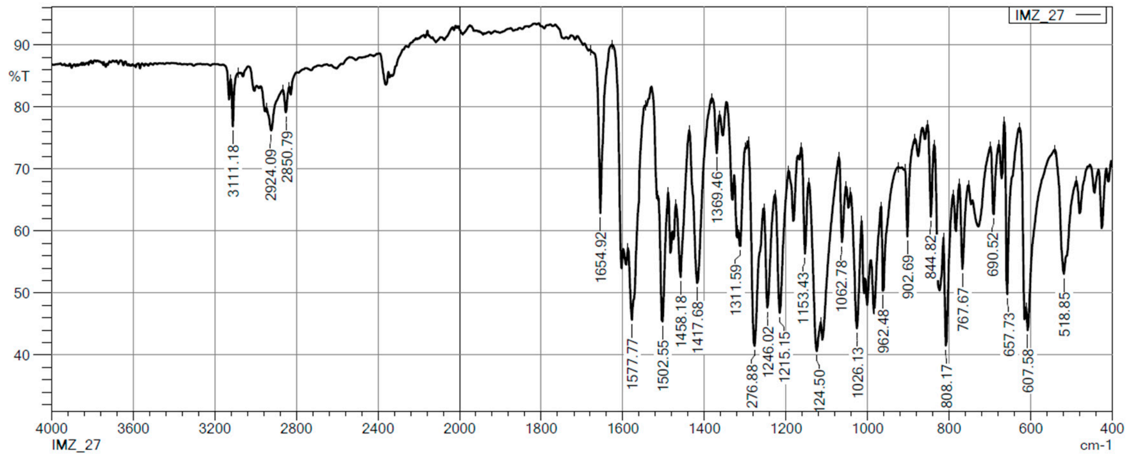

Figure S103. FTIR spectrum of the **IBC25**
